# Physics-based tissue simulator to model multicellular systems: A study of liver regeneration and hepatocellular carcinoma recurrence

**DOI:** 10.1371/journal.pcbi.1010920

**Published:** 2023-03-06

**Authors:** Luciana Melina Luque, Carlos Manuel Carlevaro, Camilo Julio Llamoza Torres, Enrique Lomba

**Affiliations:** 1 Instituto de Física de Líquidos y Sistemas Biológicos - CONICET. La Plata, Argentina; 2 Departamento de Ingeniería Mecánica, Universidad Tecnológica Nacional, Facultad Regional La Plata, La Plata, Argentina; 3 Hospital Clínico Universitario Virgen de la Arrixaca. Murcia, España; 4 Instituto de Química Física Rocasolano - CSIC. Madrid, España; Leiden University Faculty of Science: Universiteit Leiden Faculteit der Wiskunde en Natuurwetenschappen, NETHERLANDS

## Abstract

We present a multiagent-based model that captures the interactions between different types of cells with their microenvironment, and enables the analysis of the emergent global behavior during tissue regeneration and tumor development. Using this model, we are able to reproduce the temporal dynamics of regular healthy cells and cancer cells, as well as the evolution of their three-dimensional spatial distributions. By tuning the system with the characteristics of the individual patients, our model reproduces a variety of spatial patterns of tissue regeneration and tumor growth, resembling those found in clinical imaging or biopsies. In order to calibrate and validate our model we study the process of liver regeneration after surgical hepatectomy in different degrees. In the clinical context, our model is able to predict the recurrence of a hepatocellular carcinoma after a 70% partial hepatectomy. The outcomes of our simulations are in agreement with experimental and clinical observations. By fitting the model parameters to specific patient factors, it might well become a useful platform for hypotheses testing in treatments protocols.

## Author summary

We introduce an off-lattice agent-based model to simulate tissue-scale features that emerge from basic biological and biophysical cell processes. In order to calibrate and validate our model, we have considered the liver regeneration response after a 30% partial hepatectomy in which the liver recovers its original volume due to the hypertrophy of the hepatocytes. Subsequently, we have modeled the same process but after a 70% partial hepatectomy, in which the liver recovers its original volume due to the hypertrophy and the proliferation of the hepatocytes. Unfortunately, the precise mechanisms of initiating, promoting and terminating regenerative responses remain unknown. As a consequence, we have proposed a modeling approach in which such processes are regulated by a hypothetical substrate that diffuses in the cell microenvironment. As a further test, we have, in one hand, implemented our model to predict the liver response after a 50% partial hepatectomy and, on the other hand, explored our model’s ability to account for the recurrence of a hepatocellular carcinoma. The outcomes of our simulations agree with experimental data and clinical observations, which comes to underline the significant descriptive and predictive power of this computational approach. Even though our model needs to be further extended to incorporate patient specific clinical data, these results are a promising step in the direction of a personalized estimation of tissue dynamics from a limited number of measurements carried out at diagnosis.

## 1 Introduction

Many significant multicellular system problems such as tissue engineering, evolution in bacterial colonies, and tumor metastasis can only be understood by studying how individual cells grow, divide, die, move, and interact. Tissue-scale dynamics emerges as cells are influenced by biochemical and biophysical signals in their microenvironment, bearing in mind that cells themselves continually remodel their own microenvironment. Thus, the ideal scenario to study a multicellular system’s biology must simultaneously address: tissue microenvironments with multiple diffusing chemical signals (e.g., oxygen, drugs, and signalling factors), and the dynamics of many mechanically and biochemically interacting cells.

To that aim, mechanistic dynamical systems models described by ordinary differential equations have been developed [1–10] (cf. those reviewed in [11] and [12]). Even though these models are very useful, they lack the spatial resolution that would enable the examination of intratumoral heterogeneity and its correlation with treatment efficacy. This is a relevant feature since intratumoral heterogeneity has become a central element for understanding important aspects of cancer treatment such as drug resistance and biomarkers [13, 14].

A widely used modeling paradigm in the study of complex biological systems is the *agent-based model* (ABM) [15, 16]. ABMs are implemented mainly to simulate the actions, behaviors and interactions of autonomous individual or collective entities, with the aim of exploring the impact of an agent or a type of behavior in the system. Agents are independent units trying to accomplish a set of goals. The purpose of an ABM is to explore variations in the system behavior due to agent characteristics or rules. These attempt to emulate the general behavior of the system and predict the patterns of complex phenomena. Agents behave independently, but they react to the environment, modify system properties, and incorporate new agents. They also have the ability to “learn”, that is, to avoid previously unsuccessful decisions and favor successful ones, as well as to “adapt”, i.e. change their behavior in response to variations of the properties of the system. Their basic advantage is that they provide predictive power on a large scale [17].

In many cases the system being modelled is usually comprised of millions of components. In those cases a great level of abstraction/simplification must be applied to the model to render it useful [18] (we refer the reader to [19] for as extensive discussion on the accuracy of computational models). For that reason, once a preliminary model has been constructed it must be subjected to verification, calibration and validation. Verification is the process of determining how accurately prior knowledge and underlying assumptions have been translated into mathematical form. Calibration is the process by which parameters in a model are adjusted so as to match model performance to experimental data. Finally, model validation is the process of evaluating model performance against the primary design goal. In the case of biological models, this usually aims at achieving a close match between model and experiment [20].

In a medical context, ABMs should allow simulating clinical trials in sufficient detail to study the subject’s response to changes in therapy in simulations, rather than in patients. ABM have been used to study many different aspects of cancer, including tumor growth, cancer cell migration, metabolism, evolutionary dynamics, metastasis, angiogenesis and the role of cancer stem cells [21–34]. The reader is referred the extensive review by Norton and coworkers [35] for a more detailed presentation of the potential of ABMs in the context of cancer modeling.

Here, we will present a mechanistic off-lattice agent-based model to simulate tissue-scale features that emerge from basic biological and biophysical cell processes. This ABM will be validated and put to test for modeling the unusual ability of the liver to regenerate [36]. Even when 70% of its mass is surgically removed, the remnant portion expands to compensate for the lost tissue and functions [37]. The multilobular structure of the liver allows the surgical resection of a lobe of choice to achieve different degrees by partial hepatectomy (PH). Because the resection of lobes does not induce damage to the remaining tissue, PH is a clean model. Therefore, liver regeneration after PH has long been an excellent experimental model for tissue regeneration. Furthermore, although the liver consists of various types of cells, hepatocytes account for about 80% of liver weight and about 70% of all liver cells [38]. Thus, hepatocytes provide an ideal starting point to study the relation of organ size with number and size of cells.

It has been generally accepted that liver regeneration depends mainly on the proliferation of hepatocytes [37, 39, 40]. However, there are several reports showing hypertrophy of hepatocytes in the regenerated liver [41–43]. Miyaoka et al. [44, 45] performed a series of experiments and found that although a number of studies indicated that almost all hepatocytes proliferate after 70% PH, cellular hypertrophy significantly contributes to liver regeneration as well. They showed that hepatocytes undergo cell division only about 0.7 times on average in the regeneration from 70% PH, and that at early stages, the regeneration totally depends on the hypertrophy of hepatocytes. In contrast, liver regeneration after 30% PH is solely due to hypertrophy.

Therefore, liver regeneration process is a perfect scenario to test and calibrate our model. On the other hand, post-hepatectomy liver failure is a serious complication after liver resection and its incidence varies from 1.2 to 32% [46–49]. It is defined as functional deterioration of the liver associated with an increased international normalized ratio (INR)—a measurement of how long it takes blood to form a clot-, and hyperbilirubinemia typically starting after the fifth postoperative day [46]. There are recommendations that post-hepatectomy liver failure could be prevented if the remnant liver size is above 20% of its original size in patients with normal liver function, and 30 − 40% in patients with steatohepatitis or cirrhosis [50, 51]. Nevertheless, even with adequate preoperative assessments, post-hepatectomy liver failure is a major contributor to mortality rates of up to 5% after liver resection [52, 53]. Various patient-related factors (age, comorbidities such previous chemotherapy, cirrhosis, fibrosis, cholestasis, and steatosis), and surgery-related factors (extent of resection, blood loss, and ischemia reperfusion injury) affect the regenerative capacity of the remnant liver [54, 55]. Given all these numerous factors, estimating the adequate extent of the hepatectomy, and individual regenerative capacity, remain significant challenges for clinicians and scientists. It is to be stressed here that the regeneration process is controlled by different transcriptomic signatures depending on the intensity, duration and etiology of liver injury [56]. Consequently, different transcripts can modulate the results of the regeneration processes.

In any case, according to the Barcelona Clinic Liver Cancer (BCLC) staging system [57], hepatic resection (partial hepatectomy) can be considered as a curative treatment for patients with stage 0-A uninodular hepatocellular carcinoma (HCC) who maintain preserved liver function and without portal hypertension. The prognosis of HCC patients has improved because of advances in radiologic assessment, patient selection, operative techniques, and perioperative care [58, 59]. On the other hand, long-term prognosis of patients with HCC after liver resection is often affected by high tumor recurrence rates that reach 40 − 70% within 5 years [60]. This is an issue that must urgently be addressed, and where we believe that a well calibrated computational model that succeeds on modeling the liver regeneration processes and HCC evolution, would complement the clinical trials, once fed-in with specific patient data. Moreover, liver regeneration is the basic element for the maintenance of liver function and size during homeostasis and liver injury (acute and chronic). Understanding the mechanisms of hepatic regeneration, from its cellular origins and signaling mechanisms, is essential to design specific regeneration models. Making these models available in a more “accessible” way that allows for a quicker evaluation of the influence of certain factors on regeneration processes, unlike the “classical” models, would then facilitate the optimization the different diagnostic strategies (time intervals for hepatocarcinoma screening) and treatments (recurrence rate compared to ablative therapies, immunotherapy, response to sequential therapies). The approach here presented might well constitute a potential tool to evaluate biomarkers, such as circulating tumor cells (determined by liquid biopsy) and their correlation with the rate of tumor growth.

The manuscript is organized as follows: After a brief description of the biological model, our agent-based model and the tumor growth model in section 2, we calibrate and validate our model against literature data [44, 45, 61–64], for two cases of liver regeneration: 30% PH and 70% PH (Sections 3.1.1 and 3.1.2 respectively). Then we test our model for a 50% PH in section 3.1.3. Finally, we have simulated the recurrence of a hepatocellular carcinoma after a PH and present our most significant results in section 3.2. Therein, all our results are commented and analyzed in the light of experimental (data obtained on mice) and clinical data. Discussion and future prospects are presented in section 4.

## 2 Materials and methods

### 2.1 Biological model

**Fig 1:**
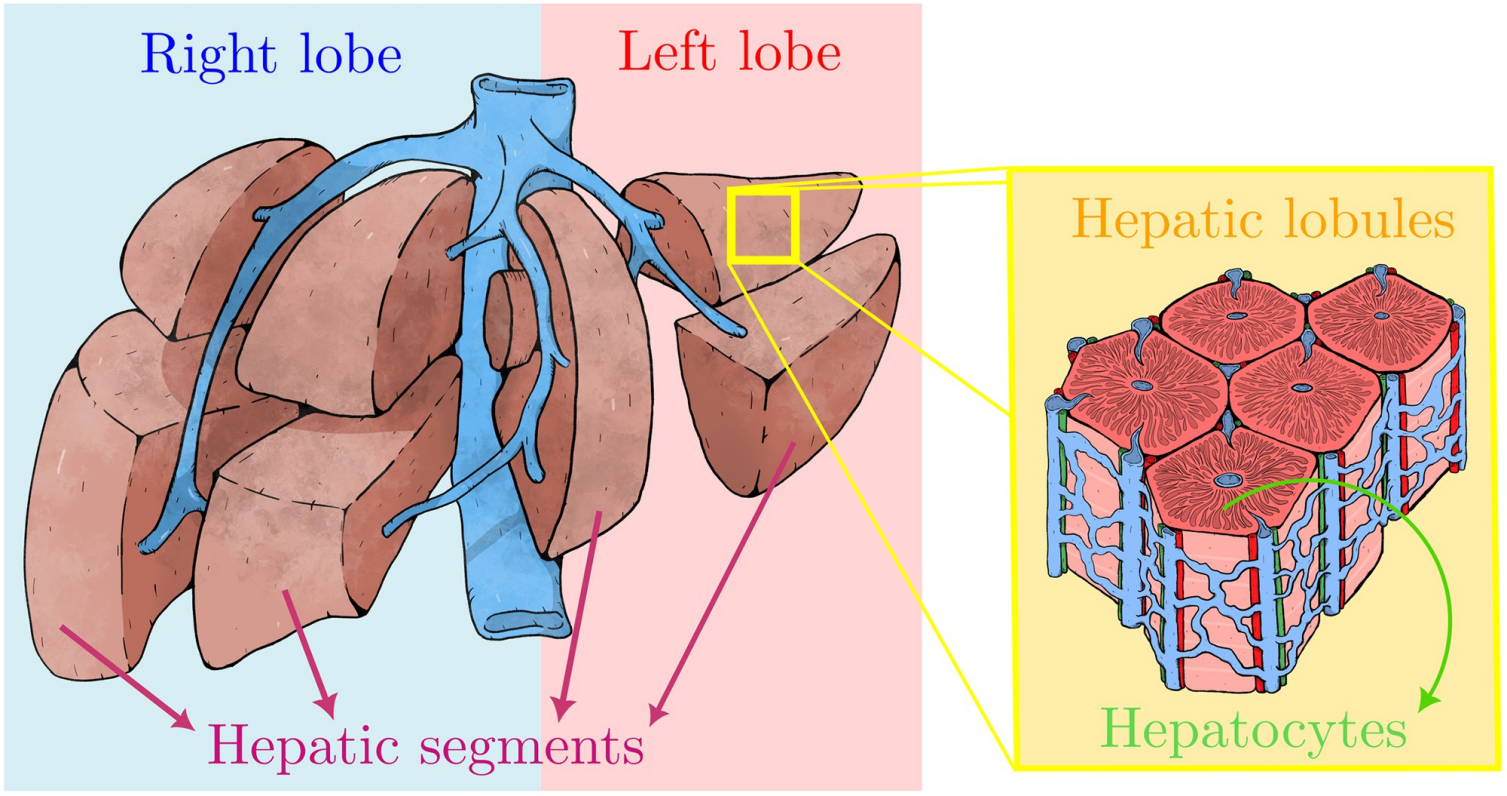
Schematic representation of the multi-scale liver architecture. The human liver is divided grossly into four parts or lobes. The four lobes are the right lobe, the left lobe, the caudate lobe, and the quadrate lobe. Seen from the front the liver is divided into two lobes: the right lobe and the left lobe. It is further divided in eight functionally independent segments based in the the Couinaud classification of liver anatomy. At the microscopic (histological) scale, the liver is organized in repetitive functional units called liver lobules, which take the shape of polygonal prisms (typically hexagonal in cross section). Each lobule is mainly constituted by hepatocytes and it is centered on a branch of the hepatic vein called the central vein which is interconnected with the interlobular portal triads: the hepatic artery (red), the portal vein (blue), bile duct (green).

The liver is a highly complex organ, which removes drugs and toxins from the blood. It is characterized by its multi-scale architecture (Fig 1) which consists of four lobes: the right lobe, the left lobe, the caudate lobe, and the quadrate lobe, which are further divided into eight segments based in the Couinaud system, also known as hepatic segments [65]. Each segment has its own vascular inflow, outflow and biliary drainage. The division of the liver into independent units means that segments can be resected without damaging the remaining segments. Hepatic parenchyma is organized in repetitive functional units called liver lobules. The lobules are roughly hexagonal prisms, and consist of plates of hepatocytes, and sinusoids radiating from a central vein towards an imaginary perimeter of interlobular portal triads. The portal triad is a distinctive component of a lobule, which can be found running along each of the lobule’s corners. It consists of the hepatic artery, the portal vein and the common bile duct. The central vein joins the hepatic artery and the portal vein to carry blood out from the liver [66]. A recent work reported that this particular structures remain during the liver regeneration process [67]. Even though hepatocytes (liver parenchymal cells) account for about 80% of liver weight and about 70% of all liver cells, the liver also has other type of cells named nonparenchymal cells: endothelial cells, Kupffer cells (macrophages resident in the liver), and biliary-duct cells [38].

One of the main characteristics of the liver is its high regenerative capacity after injury. Even when 70% of its mass is surgically removed, the remnant portion expands to compensate for the lost tissue and functions [37, 68]. Liver resection is the most common liver surgery and consists of removal of liver tissue due to focal lesions, most often malignant tumors and living liver donation [69]. The multilobular structure of the liver not only allows the surgical resection of a lobe of choice to achieve different degrees by partial hepatectomy but also the resection of lobes does not induce damage to the remaining tissue. The extent of resection is determined by the size and location of the focal lesion and the estimated function of the future liver remnant [70]. Prior to liver resection, surgeons have to assess the patient’s individual risk for postoperative liver dysfunction. In case of malignant tumors, surgeons have to identify the surgical strategy best suited to allow radical oncological removal in order to avoid recurrence, without putting the patient at risk of postoperative liver failure due to excessive removal of liver mass [71–73].

The liver regenerates in a highly organized fashion after surgery [68]. The human body responds to partial hepatectomy not by regenerating lost segments but by inducing hyperplasia in the remnant liver [36, 74, 75]. The anatomical structures of a liver that has undergone partial hepatectomy are therefore distinctly different from those of the original liver. The process of restoration of liver volume is initiated by the replication of various types of intrahepatic cells, followed by an increase in cell size. Nonparenchymal cells replicate in a delayed fashion. After replication has been completed, growth consisting of an increase in cell size occurs over several additional days. The initiation and synchronization of replication in different types of hepatic cells depend on the extent of the resection, tissue damage, or both. Low-grade tissue damage (*e.g.*, toxic or ischemic injury) or a relatively small resection (removal of less than 30% of the liver) substantially reduces the replication rate, which also appears to be less synchronized than after a large resection (removal of 70% of the liver) [36, 68, 75].

Since all our results are compared with experimental data obtained from rodent models, it is important to mention that the process of hepatic regeneration in rodents and humans is similar, and the results obtained from rodents could be applicable to the human liver [76]. Moreover, the rat liver architecture can be compared with the human liver segmentation defined by Couinaud [77].

### 2.2 Computational model

Our model is implemented resorting to an object oriented programming model, and to that aim we have used C++11 language. Simulation CPU time depends on model parameters such as domain (lattice) size, cell number and simulation length (in time); a typical simulation run takes approximately 6 h on a single core of an Intel i7–10510U CPU. Model visualization is performed with Ovito [78], Paraview [79] and Matplotlib [80].

In order to reduce the computational burden of the simulations, an abstraction process was necessary to go from the biological to the computational model. First, we disregard the explicit liver geometry, instead we have chosen a reduced spherical model. This simplification is possible because, as it was mentioned in section 2.1, after a PH the liver does not regenerate the lost segments, *i.e.* does not recover the original shape. Instead it just recovers its original volume by hyperplasia of the remaining lobes. Subsequently, we rather focus our attention on the liver parenchyma instead of liver lobes. In our computational model, hepatic lobules are hexagonal prisms delimited by an imaginary perimeter of interlobular portal triads. The central vein that carries the blood out from the liver as well as the liver sinusoids are not explicitly modeled as the portal triads are. We rather model this behavior by tuning the effective oxygen difussion and decay constants. A discussion on the implications of these simplifications on the results will be presented in section 4.

**Fig 2:**
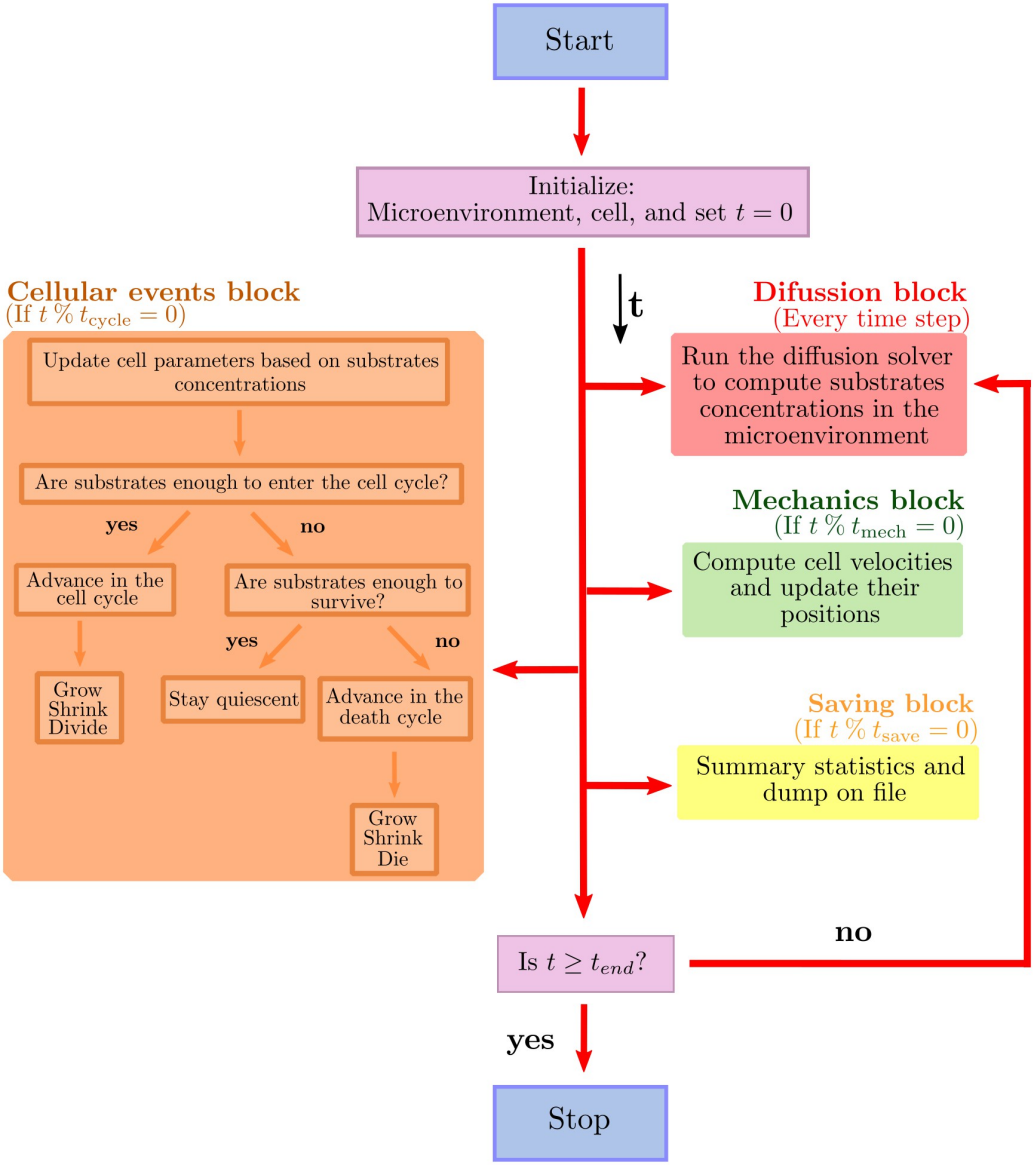
Main loop flow diagram. Blue box represents the start of the program. Red box represent the diffusion processes. Green box and orange box describe the cell mechanics and cycling processes respectively. Finally, yellow boxes represent the data saving process. After initializing the microenvironment, the cells, and the current simulation time *t* = 0, our model tracks (internally) *t*_mech_ (the next time at which cell mechanics methods are run), *t*_cycle_ (the next time at which cell processes are run), and *t*_save_ (the simulation data output time), with output frequency Δ*t*_save_. % represents the modulo operation.

In the following subsections we will describe the methods implemented to model diffusion and cellular mechanics, as well as the mathematical models to predict tissue growth kinetics. For further details, we refer the reader to the supplementary material S1 Text. A schematic representation of the inner workings of our model is depicted in Fig 2. The key elements of the model are described in what follows.

#### 2.2.1 Diffusion solver

We model the diffusion of chemical substrates in the tumor microenvironment as a vector of reaction-diffusion partial differential equations for a vector of chemical substrates, *ρ*. It is discretized over a Cartesian mesh for computational convenience, in such a way that each voxel (volumetric pixel) stores a vector of chemical substrates. Each substrate diffuses and decays, and can be secreted or uptaken by individual cells at their specific positions.

We use a first order, implicit (and stable) operator splitting, allowing us to create separate, optimized solvers for the diffusion-decay, cell-based source/sinks [81]. The diffusion-decay terms are solved using the finite volume method [82], further accelerated by an additional first-order spatial splitting in the *x*−, *y*− and *z*−directions via the locally one-dimensional method (LOD) [81, 83]. For each dimension, we solve the resulting tridiagonal linear systems with the Thomas algorithm [84].

We also implement the so-called Dirichlet nodes, so that substrate values at any voxel within the simulation domain can be overwritten to turn the voxel into a continuous source of substrates. This is particularly useful to model the effect of blood vessels, or when applying Dirichlet boundary conditions.

For computational efficiency we use thread parallelization to relevant loops, e.g. in many instances of the Thomas solver when solving *x*−diffusion across multiple strips of the solution domain. This methods were already implemented and successfully tested by Ghaffarizade et al. [85], therefore, we have validated the numerical accuracy of our solver by comparing our results with those found in Reference [85]. For further details, please refer to the supplementary material S1 Text.

#### 2.2.2 Cell agents

Since we are implementing an agent-based model programmed in the context of an object oriented approach, each cell is an agent implemented as a software object that acts independently. Like most classes in software it has *attributes*, *i.e.* its own internal variables that each specific agent is allowed to manipulate on its own. It also has *methods*, which are functions that act upon the attributes. In the context of cell biology, relevant attributes might be: position, size, cell state regarding the cell cycle, etc. The cell cycle is an object with the aforementioned attributes. The cell class have methods that represent cellular processes such as, death, growth and volume change, and are coordinated by the cell cycle object.

One of the main features of our model is that cells are *off-lattice*. Consequently, they are not confined to a particular lattice or spatial arrangement, they move in a continuous fashion through all space positions, and therefore underlying possible artifacts associated with the chosen lattice structure and spacing are removed.

Based on previous cell-based models [33, 86, 87], we have modeled the cell behavior as follows:

**Cell cycle:** We model the cell cycle as a directed graph, in which the nodes represent the phases and the edges the transition rates between them. These transition rates can take stochastic or constant values. Moreover, any of the cell cycle time scales can be adjusted at the beginning of the simulation to match different types of growth and they can also be adjusted at any time on an individual cell in order to reflect its microenvironment influences.

Our model allows to implement different types of cell cycles based on different parameters. Following Miyaoka and coworkers [44, 45], we can base our cell cycle on a tracking of the expression of protein Ki-67, involved in cell proliferation. This tracking is performed thanks to a nuclear stain of Ki-67. We note in passing that Ki-67 protein expresses in the *S*, *G*2, and *M* phases [88], and to a lesser extent in the *G*1 phase [89]. It is seen in post-mitotic daughter cells [90], but it is not produced in these cells [88]. Instead, any remaining Ki-67 protein in post-mitotic cells is degraded quickly, with a half-life of 60 − 90 minutes [88]. We can base our cell cycle by setting the phase “Ki-67+ pre-mitotic” to be the combined duration of *S*, *G*2, and *M* phases, which are relatively fixed compared to the duration of *G*0/*G*1 [91, 92]. We can set the second phase “Ki-67+ post-mitotic” at the *G*1/*G*0 phase, to be on the order of two Ki-67 half-lives. Then, the remaining *G*1/*G*0 phase will be the “Ki-67-” phase. For further details, please refer to the supplementary material S1 Text.

Finally, since at cell scale death is not an instantaneous event but a process, we model death cycles such as Necrosis and Apoptosis as we did with cell cycles, by using directed graphs, death is part of the cell cycle after all. The entry to the death cycles depends on the resources, for example oxygen, drugs, therapies, etc.

**Cell volume:** To model cell volume variation, each cell tracks *V* (total volume), *V*_*F*_ (total fluid volume), *V*_*S*_ (total solid volume), *V*_*NS*_ (nuclear solid volume), *V*_*CS*_ (cytoplasmic solid volume), *V*_*N*_ (total nuclear volume), and *V*_*C*_ (total cytoplasmic volume). Key parameters include nuclear solid, cytoplasmic solid, and fluid rate change parameters (*r*_*N*_, *r*_*C*_, and *r*_*F*_), the cell’s “target” fluid fraction *f*_*F*_, target solid volume $V_{NS}^{*}$, and target cytoplasmic to nuclear volume ratio *f*_*CN*_. For each cell, these volumes are modeled with a system of ordinary differential equations that allow cells to grow or shrink towards a target volume. These parameters are updated as the cell progresses through its current cycle or death cycle.

**Cell mechanics:** In our model, as in [33], we use the piecewise polynomial force which is constructed as the sum of a positive adhesive and a negative repulsive polynomial force contributions. It is important to note that repulsive forces are really an elastic resistance to deformation. To compute these forces we use adhesive and repulsive interaction potentials functions.

We assume that three types of forces act upon each cell. First we have a drag force, which represents dissipative drag-like forces such as fluid drag and cell-extra cellular matrix adhesion. We then have neighboring cell mechanical forces. In the simplest case these involve repulsive forces due to limited cell compressibility, but they usually also include cell-cell adhesion. We use interaction potentials that depend upon each cell’s size, maximum adhesion distance, adhesion and repulsion parameters and distance to other cells. Finally, the third force acting on the cells is the cell-basement membrane interaction.

Since the cell microenvironment has a very low Reynolds number (the Reynolds number describes the ratio of inertial to viscous forces) [93], inertial effects such as acceleration are neglected. This is commonly known as the inertialess assumption, and implies that forces equilibrate at relatively fast time scales in contrast to the processes involved in cell cycling, death cycling, and volume variation.

We finally use the Adams-Bashforth method [94] to solve the mechanics equation to enhance computational efficiency.

We refer the reader to the supplementary material S1 Text for further details on the implementation of the piecewise polynomial force model.

**Cell secretion and uptake:** This is one of the most important data structures of the cell because it links the cell with its microenvironment. We solve a vector of partial differential equations, which reduce to the addition of a cellular secretion/uptake term to the diffusion equation described in section 2.2.1.

This is very important since most of the cellular processes depend on the substrates that diffuse in the microenvironment. For example, it is well accepted that after a partial hepatectomy, the liver undergoes to cytokine- and growth factor- mediated regeneration processes [95]. However, most of the mechanisms of initiating and promoting regenerative responses as well as the termination of liver regeneration remain unknown [44, 45]. In this work cellular proliferation is assumed to be controlled by a growth factor that diffuses through the microenvironment. This growth factor is only considered as an abstract parameter which encompasses all the underlying molecular mechanisms involved in the liver regeneration process. The cell cycle entry rate is proportional to this factor in the following way:

$$\begin{array}{c} r=\frac{1}{t_{K^{-}}}\text{max}\{(\frac{GF-GF_{\text{prol}}}{GF^{*}-GF_{\text{prol}}}),0\} \end{array}$$ (1)

where $t_{K^{-}}$ is the mean time each cell spends in the non-proliferative phase (see section 2.2.2), which can be experimentally monitored using the Ki-67 cellular marker. *GF* is the current growth factor concentration value in the cell’s voxel, *GF*_prol_ is the proliferation threshold, *i.e.* the growth factor value below which the proliferation ceases and GF* is the proliferation saturation value, above which the proliferation rate is maximized. Therefore, based on the growth factor concentration, the hepatocyte will enter either the hypertrophy phase or the proliferation phase. A similar approach can be done based on the oxygen concentration, however, instead of influence on the decision about whether or not to proliferate, oxygen will accelerate the phase entry. Please refer to the supplementary material S1 Text for further information about how cell cycles and transition rates will be modified based on chemical substrates concentrations.

### 2.3 Growth estimations

To complement our model in the prediction of tumor growth and/or tissue regeneration we use a mathematical model known as the *Gompertz model* [96–99]. This model assumes initial exponential growth, but as the tumor grows the volume-doubling time increases due to lack of nutrients and subsequent cell death, by which the growth rate shifts towards linear regime, finally reaching a plateau [96]. This is given by:

$$\begin{array}{c} V\left(t\right)=K\;\text{exp}[\text{log}(\frac{V_{0}}{K})\text{exp}(-\alpha t)] \end{array}$$ (2)

where the parameter *K* is the carrying capacity of the tissue, which is the highest possible volume, *V*_0_ is the initial volume of the tissue and *α* is the specific growth rate [100, 101] which is defined by

$$\begin{array}{c} \alpha =\frac{\text{log}(\frac{V_{2}}{V_{1}})}{t_{2}-t_{1}}. \end{array}$$ (3)

Here *V*_1_ and *V*_2_ are tumor volumes at the measure times *t*_1_ and *t*_2_ respectively. This parameter *α* determines how fast the tumor reaches the carrying capacity *K* and its measured in (%/days).

## 3 Results

**Fig 3:**
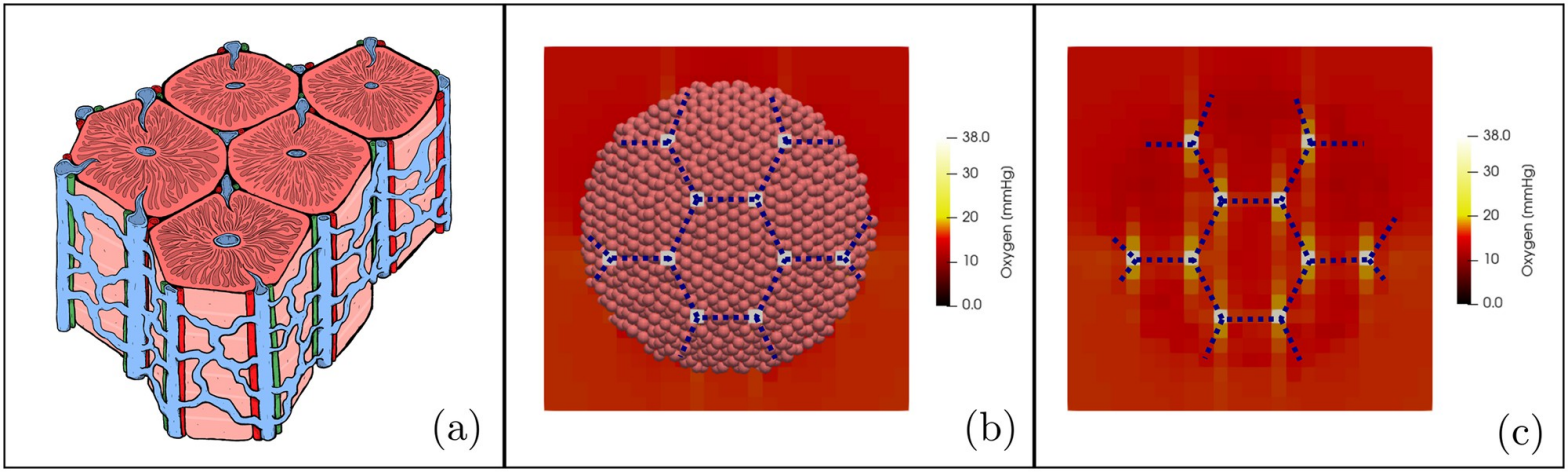
Liver blood vessels architecture. **(a)** Schematic representation of the hepatic lobules. They consist of plates of hepatocytes, and sinusoids radiating from a central vein interconnected with the interlobular portal triads: the hepatic artery (red), the portal vein (blue), and the common bile duct (green). **(b)** computational model of the liver parenchyma in which we can observe the blood vessel architecture. **(c)** Heat map of the oxygen diffusion in the liver microenvironment. Blue dotted lines were drawn just as a guide to the eye.

We first attempted to define a baseline scenario of the liver physiology. As it was mentioned before, we focus our attention in the liver parenchyma which, in our model, is made up of hepatic lobules which are hexagonal prisms delimited by an imaginary perimeter of interlobular portal triads. We idealized this dynamic vasculature architecture (Fig 3a) by using the Dirichlet nodes as shown in Fig 3b and 3c, and defining the distance between triads on micrograph analysis [66]. Fig 3b shows a transversal cut of our simulated liver in which we can observe the hepatic lobules architecture. Blue dotted lines were drawn just as a guide to the eye. Pink spheres represent the hepatocytes and the white squares represent the portal triads that oxygenate the tissue. Fig 3c shows the heat map of the oxygen diffusion in the liver micoenvironment. The oxygen diffuses from the portal triads (Dirichlet nodes), there is no diffusion from the boundaries of the simulation box.

The grid we used to simulate liver regeneration is 1 mm^3^ in total size, orders of magnitude smaller than an actual liver. While the model does not place limitations on the liver size, the implementation is obviously constrained by the size of the computer memory. However, this limitation can be to some extent circumvented by considering the sample region as representative of a significant sub-region of the liver. Obviously size effects must be investigated in further detail to the extent that new algorithmic and hardware improvements become available (e.g. by exploiting massively parallel CUDA [102] cores and/or Tensor cores in new GPU/TPU architectures).

All the parameters values that were used in the following examples, and the corresponding sources are listed in section 4 of the supplementary material S1 Text.

### 3.1 Liver regeneration

**Fig 4:**
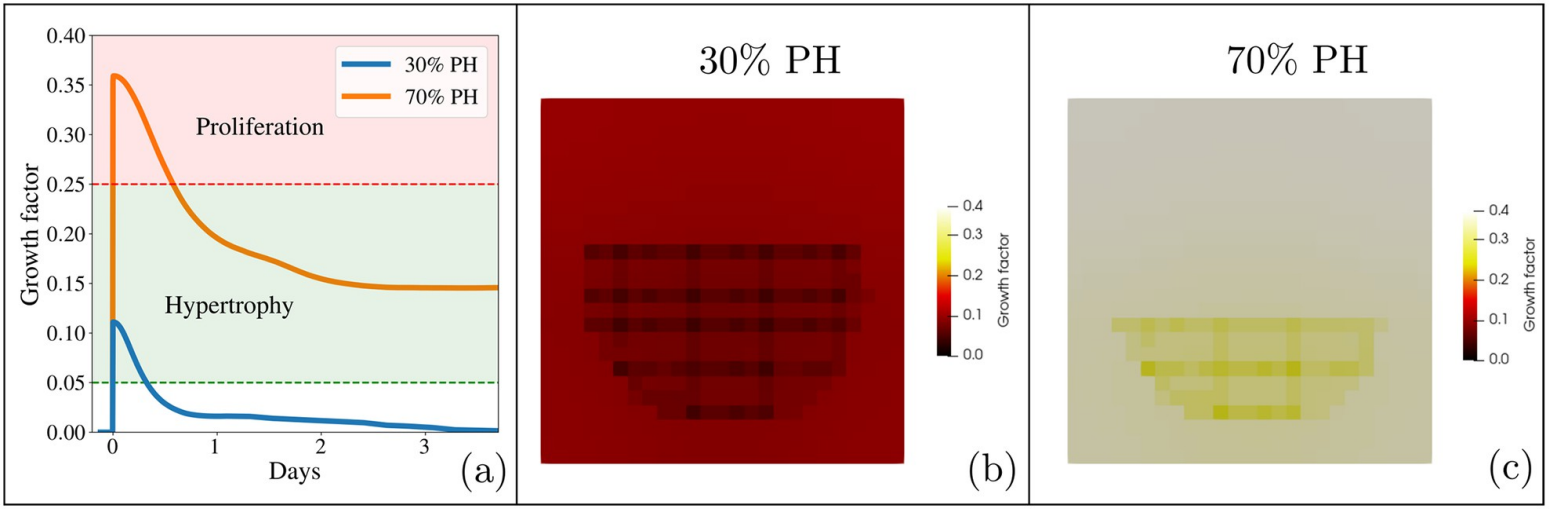
Growth factor. (a) Growth factor concentration in the liver microenvironment after a 30% PH (blue line) and a 70% PH (orange line), and the response it will cause in the hepatocytes. (b) and (c) Heat map of the growth factor concentration after 30% PH and a 70% PH respectively. The concentration is greater in the 70% PH.

Our first experiments aim at assessing the ability of our model to describe the dynamics of the liver regeneration process. We performed the *in silico* version of the experiments of Miyaoka et al. [44, 45], with the novelty that hepatocytes secrete and are sensitive to a growth factor that diffuses through the microenvironment creating a gradient. We assume that when the liver is intact, the microenvironment is in homeostasis and, although they are metabolically active, hepatocytes remain dormant in the cell cycle. When a partial hepatectomy occurs, each cell secretes a constant amount of growth factor depending on the extent of the injury. They will undergo either hypertrophy or proliferation as regulated by the concentration of the growth factor. This is illustrated in Fig 4. In panel (a), we can see a peak of growth factor when the partial hepatectomy occurs. A 30% PH does not generate enough growth factor to make hepatocytes proliferate (Fig 4b). On the contrary, a 70% PH not only produces enough growth factor to make the hepatocytes proliferate, they also undergo an hypertrophy process (Fig 4c). Finally, as the liver regenerates, this growth factor decreases.

#### 3.1.1 30% partial hepatectomy

**Fig 5:**
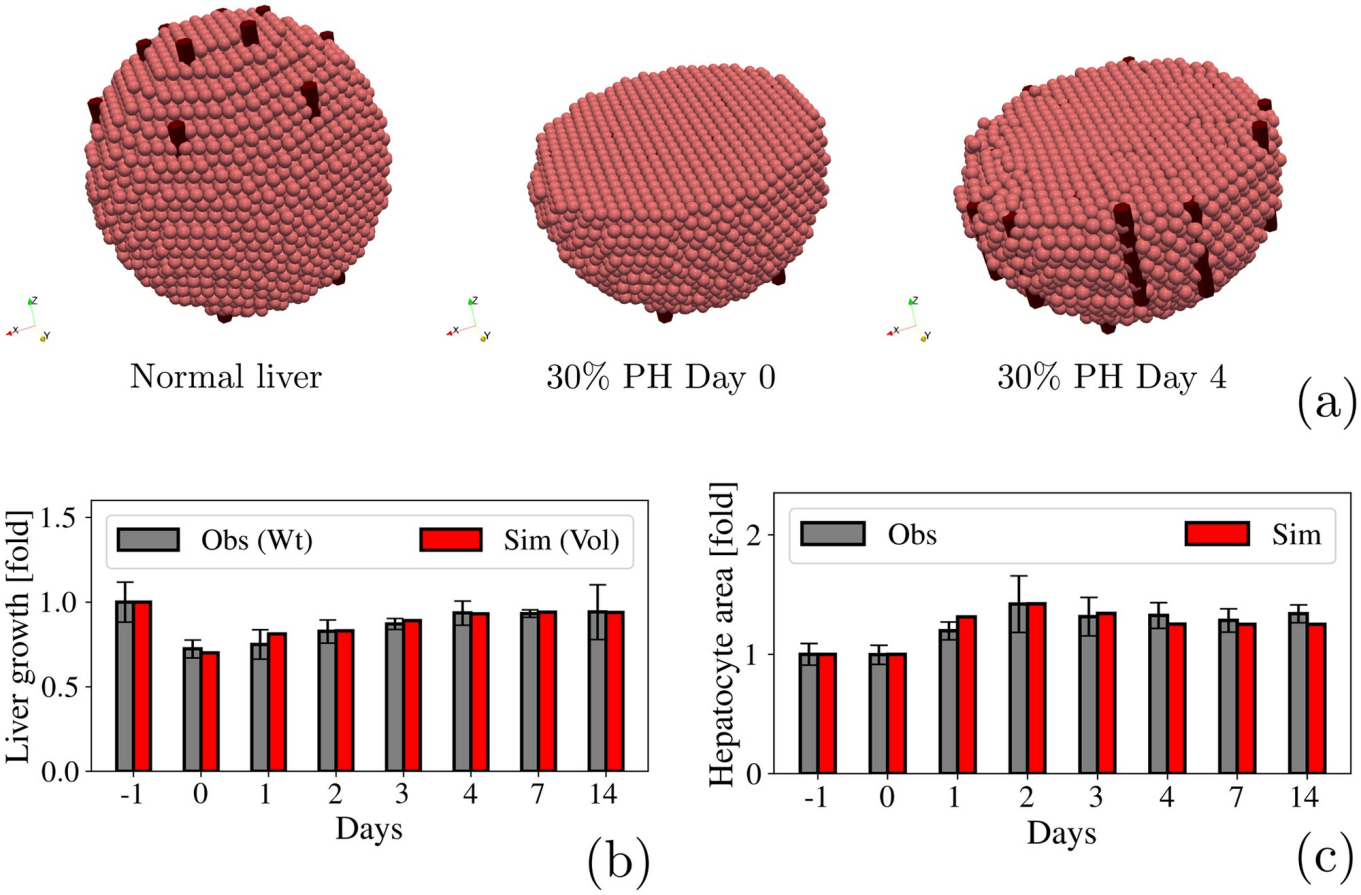
30% PH. **(a)** Qualitative representation of the liver regeneration process after a 30% PH. **(b)** Fold-increase in the liver size. Observational data is shown in gray and represents the weight of the liver measured by Miyaoka et. al [44, 45]. Simulated data is shown in red and represents the liver volume. **(c)** Quantification of the hepatocytes area during liver regeneration.

We first analyzed liver regeneration after a 30% PH. We have set the hepatocytes cell cycle time 33.6 hr as stated in ref. [103] but as mentioned in section 2.2.2, this could vary depending on the oxygenation of the tissue and the growth factor concentration. Fig 5a and 5b illustrate the regeneration process in a qualitative and a quantitative manner respectively. We found that liver volume increased from 1 day after the PH and reached a plateau of 0.93-fold of the liver initial volume, by 4 days. Quantification of the hepatocytes reveals that there was no proliferation but hypertrophy. As shown in Fig 5c, hepatocytes reached the largest size 2 days after PH, increasing by a factor 1.6, and then gradually decrease their size only by a 1.4 factor, thus remaining larger than before the PH. This amounts to a 1.5-factor increase in volume, suggesting that cellular hypertrophy alone compensates the lost tissue.

Fig 5b and 5c show our results (red bars) in agreement with those collected from the experiments of Miyaoka et al. [44, 45] (gray bars). It is important to mention that since our model does not account for explicit changes is liver’s weight, we have analyzed its volume instead.

An animation of the liver regeneration process after a 30% partial hepatectomy can be seen in the Supplementary Material S1 Video.

#### 3.1.2 70% partial hepatectomy

**Fig 6:**
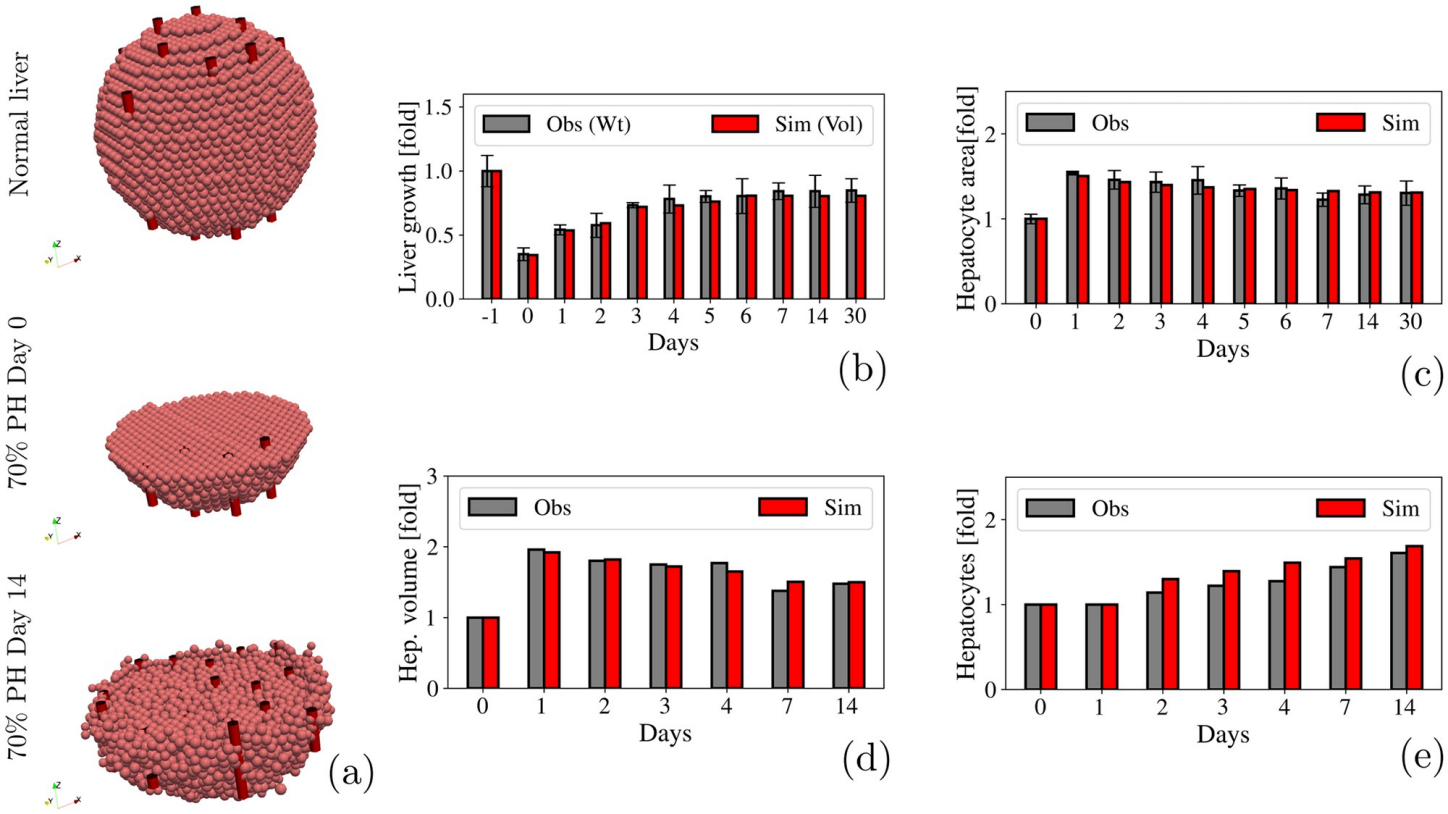
70% PH. **(a)** Qualitative representation of the liver regeneration process after a 70% PH. **(b)** Fold-increase in the liver size. Observational data is shown in gray and represents the weight of the liver measured by Miyaoka et. al [44, 45]. Simulated data is shown in red and represents the liver volume. **(c)** Quantification of the hepatocytes area during liver regeneration. **(d)** Quantification of the hepatocytes volume during liver regeneration. **(e)** Fold-increase in the number of hepatocytes during liver regeneration.

After a 70% PH, we found that liver volume increased from day 1, reaching a plateau with a total increase of 0.72-fold of the liver initial volume in day 7. Fig 6a and 6b illustrate this process in a qualitative and a quantitative fashion respectively. Hepatocytes entered the cell cycle 2 days after the PH, as shown in Fig 6e. Although there were a few active proliferating hepatocytes on day 1, liver volume had increased considerably by that time. Then we measured the area of the hepatocytes (Fig 6c) and found that hepatocytes increased their volume by 2.0-fold the first day and gradually decrease by 1.5-fold the 14^*th*^ day after the PH (Fig 6d). These results are in agreement with those obtained by Miyaoka et al. [44, 45], and indicate that proliferation of hepatocytes alone could not account for the recovery of liver mass after a 70% PH.

The animation of the liver regeneration process after a 70% partial hepatectomy, can be seen in the S2 Video.

Although the exact mechanisms underlying liver regeneration have not yet been fully characterized, studies have shown that after 70% PH many of the upregulated growth factors in a regenerating liver are known for their angiogenic properties in vivo. For instance, vascular endothelial growth factor (VEGF) is upregulated after PH [104–106]. It is a major pro-angiogenic factor [107] and is thought to improve sinusoid reconstruction during the liver regeneration process [108]. In the processes of angiogenesis associated with tissue regeneration, two phases are described; an induction phase and another proliferative angiogenesis phase. In post-hepatectomy liver regeneration models, the first is calculated during the first 4 days and the second between days 4 − 12 [109].

**Fig 7:**
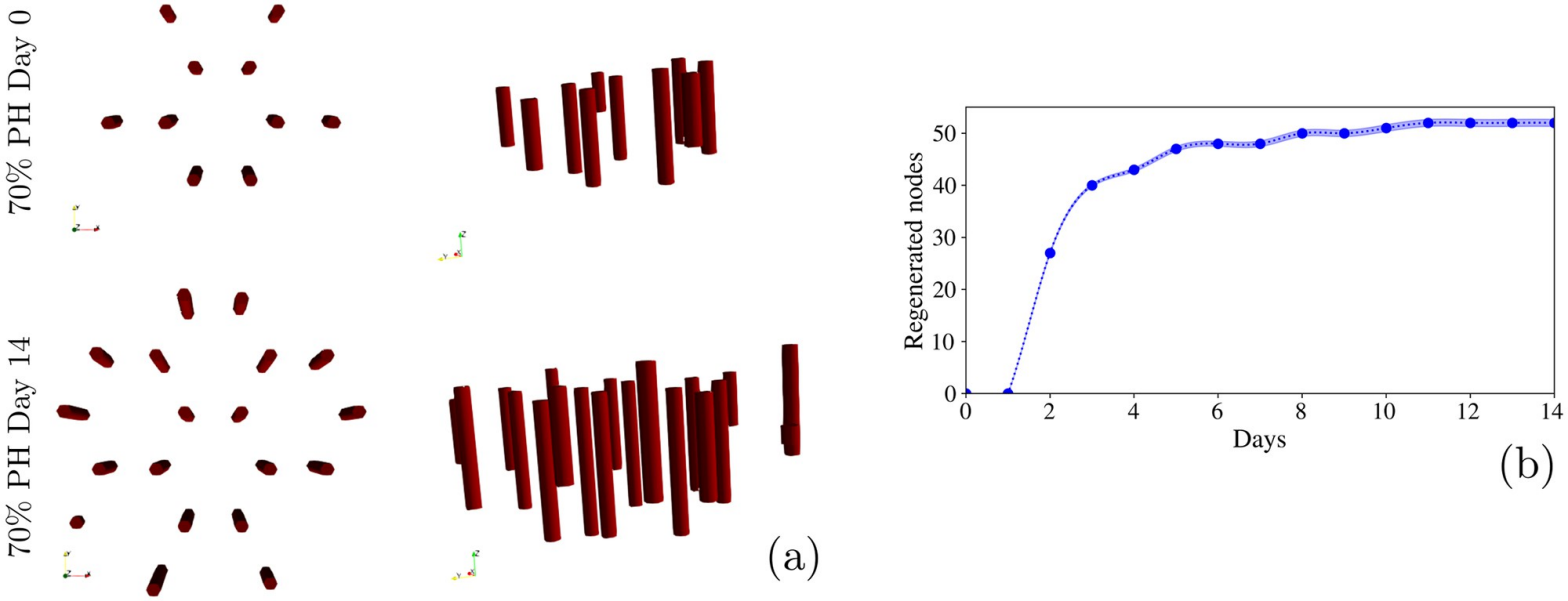
Blood vessels regeneration. **(a)** Schematic representation of blood vessels regeneration after a 70% PH. Left panels show an upper view while right panels show a side view. Blood vessels regenerate by keeping the hepatic lobules structure. **(b)** Quantification of the blood vessels regeneration by counting the Dirichlet nodes added during the regeneration process. It shows a significant increase during the first 3 days until it finally reaches a plateau. Shaded region represent the standard deviation of 40 simulations.

We have quantified blood vessel formation by counting the Dirichlet nodes added during the liver regeneration process. Fig 7a and 7b represent this process in a qualitative and a quantitative way, respectively. On one hand we found that during the regeneration process, blood vessels keep the hepatic lobules architecture (Fig 7a). To model this behavior, we have labelled each voxel according to its potential to become a blood vessel, inspired by the hepatic lobule architecture. During the simulation, if the voxel is tagged as a potential blood vessel and contains five or more cells, it turns into a Dirichlet node, that will provide oxygen to the cell microenvironment. As shown in Fig 7b, we found that the number of Dirichlet nodes increased significantly during the first 3 days following PH until they finally reach a plateau. These results are in agreement with ref. [110], in which it was found that the micro-vasculature density increased significantly during the first 3 days in mice subjected to PH. This proves that our model has the ability to simulate cell population behavior, and subsequently it also can predict blood vessel formation.

#### 3.1.3 Test of a 50% partial hepatectomy

Although 70% PH is the most studied instance of liver regeneration [36, 111], resection of approximately half volume of the donor liver is more common in the case of living donor liver transplantation setting [68]. Moreover, as it was mentioned before, resection must be more conservative in the presence of underlying liver diseases or in elderly patients (e.g., ≥ 70 years of age) [68]. Major (> 50%) hepatectomy in the presence of cirrhosis or steatosis significantly increased morbidity [112]. With the presence of liver steatosis, 30% or more of the remnant liver should remain in order to maintain viability. Furthermore, studies have revealed an increased benefit of ischemic preconditioning in patients with hepatic steatosis who had lower resected liver volume (< 50%) [113], and extensive resections are generally not recommended for patients with cirrhosis [114]. Hemihepatectomy (*i.e.* 50% PH) has now been successfully and frequently used for surgical removal of liver associate tumors and cancers [115, 116]. However, to the best of our knowledge, a study of liver regeneration dynamics after a 50% PH, such as the one presented in [44, 45] for 30% and 70% PH, has not been reported yet. Using the calibration of the previously studied PH, we have applied our model to study the liver regeneration process after a 50% PH.

**Fig 8:**
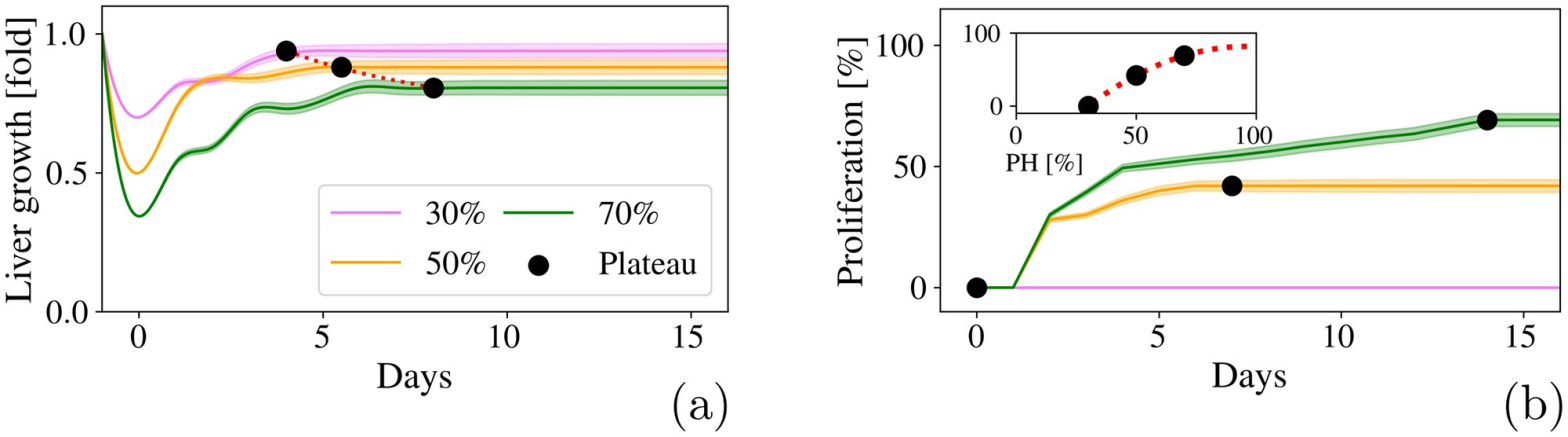
Predictions of a 50% PH compared to the outcomes of 30% and 70% PH. **(a)** Fold-increase in the liver volume after 30% (violet), 50% (orange) and 70% (green) PH. Black dots represent the time at which the volume increase reaches a plateau and the red dotted line is a polynomial fitting. **(b)** Percentage of hepatocytes that proliferates during liver regeneration. Black dots represent the time at which the proliferation increase reaches a plateau. **Inset:** Proliferation percentage in terms of the PH. The red dotted line represents a polynomial fitting. Shaded regions represent the standard deviations of 40 simulations.

We found that the liver volume increased from day 1 until it reaches a plateau with a total increase of 0.86-fold of the liver initial volume, within 5.5 days. Yoshioka et al. [117] reported that 3 days after a 50% PH, the remnant liver weight reached 0.72-fold ±0.05-fold of its original calculated weight, which is similar to the 0.78-fold that our model predicted for the third day after the PH. As shown in Fig 8a, we can apply a polynomial fitting that matches our simulations and estimates the outcomes of the hepatectomies. Fig 8b shows that, similar to the 70% PH, the hepatocytes not only undergo hypertrophy, they also proliferate. We found that hepatocytes undergo cell division only about 0.42 times on average and reaches a plateau within 7 days. If we plot the percentage of hepatocytes that proliferates in terms of the the amount of liver removed, as shown in the inset of Fig 8b, we can we can apply a polynomial fitting to estimate the outcomes of different PH.

It is important to mention that the model is calibrated based on an normal hepatic parenchyma, i.e. without liver cirrhosis, with preserved liver function and no signs of portal hypertension, therefore its predictions correspond to that specific case. However it could be adjusted to model the presence of underlying liver diseases.

### 3.2 Recurrence of hepatocellular carcinoma

With the scenario of the liver regeneration working properly, we are now interested in study tumor recurrence. The need for extended liver resection is increasing due to the growing incidence of liver tumors in aging societies [71], however, the resected volume not only depends on the tumor volume itself but also on the patient’s liver overall health. Prior the resection, surgeons not only have to assess tumor resection to avoid recurrence but also the patient’s individual risk for postoperative liver dysfunction. It is well known that planning for a safe resection of a liver tumor with a large future liver remnant reduces the risk for postoperative liver failure but increases the risk of tumor recurrence. In contrast, planning for an oncologic radical surgery requires to remove a safety margin. Extending the safety margin in case of a centrally located tumor leads to a substantially extended resection leaving a rather small future liver remnant behind, which increases the risk of postoperative liver failure [71]. In a patient with uninodular hepatocarcinoma or up to 3 nodules smaller than 3 cm each without macrovascular infiltration, without distant metastasis (BCLC Stage 0-A), with preserved liver function and without portal hypertension, a liver resection of up to 60 − 70% is a feasible scenario considered in the BCLC classification [57], as long as it presents a normal hepatic parenchyma. Preexisting liver disease such as steatosis increases the risk for postoperative liver failure and might therefore call for a smaller PH compared to livers without preexisting diseases, however, the study of liver and hepatocellular carcinoma dynamics with preexisting diseases exceeds the scope of this paper.

Following the previous case in which we have considered a normal hepatic parenchyma, we have seeded a remaining tumor clone in order to model a recurrence of a hepatocellular carcinoma after a extended resection, *i.e.* a 70% PH. As proof of concept, we have assumed that preserved liver function without portal hypertension could approximate the behavior of a liver with preserved or normal parenchyma.

**Fig 9:**
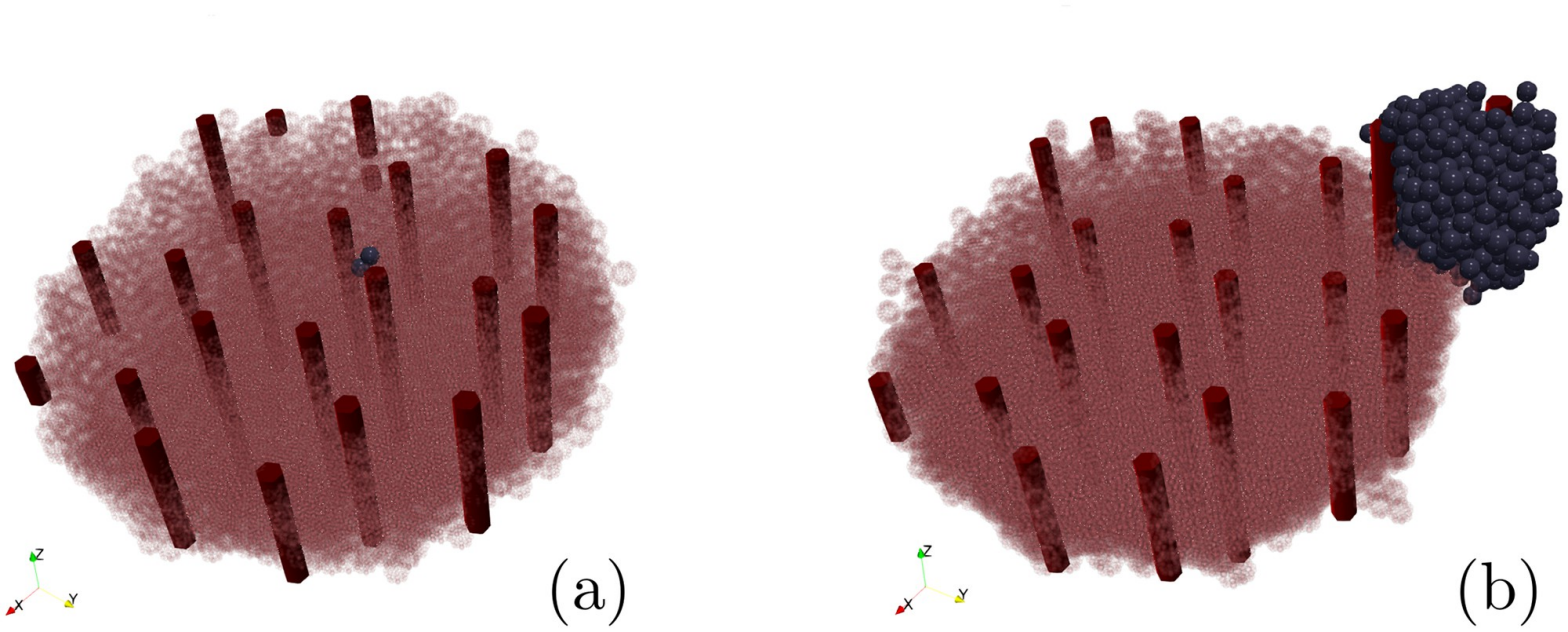
Random initial position. Qualitative representation of the liver regeneration with HCC recurrence, 30 days after the PH. Hepatocytes are represented by transparent pink cells to have a better view of the blood vessels (red tubes) and the tumor (blue cells) growth. **(a)** The slowest tumor growth happens when the residual tumor clone was seeded in the center of the liver surface. **(b)** The fastest tumor growth happens when the residual tumor clone was seeded in the periphery of the liver.

Cancer cell cycle has a duration of 38.6 hr [103] but the rate of cycle entry scales proportionally to oxygen concentration. As a first step, we have randomly seeded the tumor clone over the liver surface in order to test if its initial location would change the simulation outcomes. We have performed 40 simulations and obtained a 59% standard deviation of the tumor final size. Fig 9 shows the smallest (a) and the biggest (b) tumors, 30 days after the PH. Hepatocytes are drawn transparent to have a better view of the blood vessels (red tubes) and the tumor (blue cells) growth. We observe that the tumor volume vary depending on the location of the residual tumor clone, whether it is in the periphery or the center of the liver surface. This might be due to the process of blood vessels generation implemented in our model (sec. 3.1.2). When the tumor clone is located in the center of the liver surrounded by hepatocytes, it will have to compete for oxygen with the surrounding cells (*i.e.* share the preexisting blood vessels), reducing the cell cycle entry. On the contrary, when the tumor clone is located in the periphery, it will grow outwards. That means that the tumor cell not only has more oxygen for itself due to the liver blood vessels, but as the tumor gets bigger, it generates its own blood vessels, as shown in Fig 9b, which increases the cell cycle entry. In clinical practice, among the factors that have been reported as being associated with early or late recurrence, there are characteristics such as tumor size and number of tumors before PH, micro and macrovascular invasion, degree of tumor differentiation, alpha-fetoprotein levels [118–124] but how tumor location in the liver will influence the HCC recurrence has been poorly described and with heterogeneous results [125–128].

**Fig 10:**
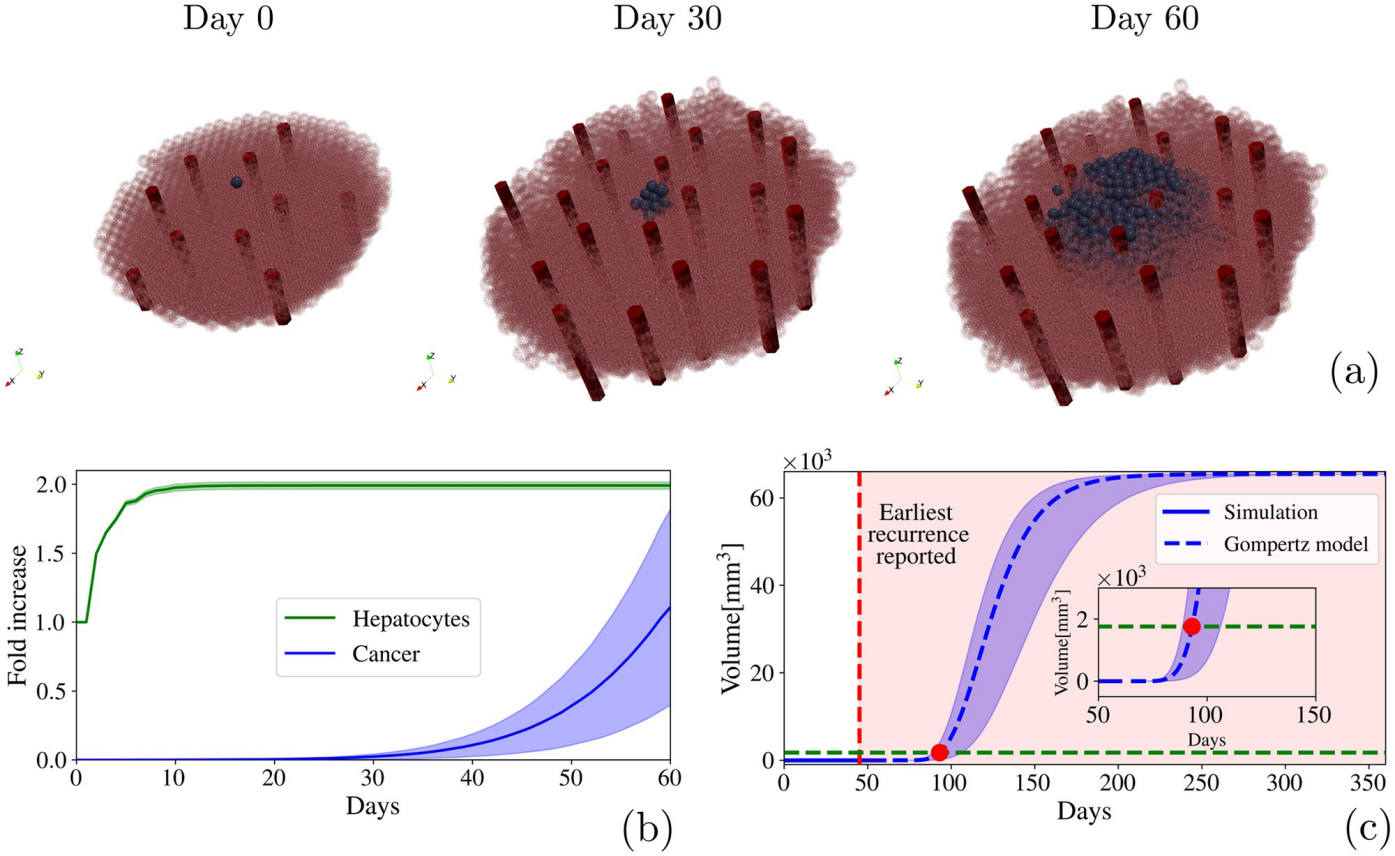
Hepatocellular carcinoma. **(a)** Schematic representation of the recurrence of a hepatocellular carcinoma after a 70% PH. Hepatocytes are represented by transparent pink cells to have a better view of the blood vessels (red tubes) and the tumor (blue cells) growth **(b)** Fold-increase in liver regeneration and hepatocellular carcinoma recurrence. **(c)** Time based volume estimation of the hepatocellular carcinoma by using simulation data (blue line) to adjust a Gompertz model growth curve (blue dotted line). Red dotted line represents the earliest recurrence reported in clinical observations and the red point along with the green dotted line,represents the minimum volume that the tumor must reach to be detected (5 mm) by imaging diagnose.

We have considered the mean value of our simulations and analyze the recurrence dynamics. We observe that, despite the initial location, there is a delay in the cancer cell reactivation in comparison to the liver regeneration process. Fig 10a shows the process in a qualitative fashion. Cancer cells start growing after the liver finishes its regeneration process. In this case, they grow inwards, following the increase of oxygen concentration, and consequently they proliferate towards the blood vessels (the animation of the HCC recurrence can be seen at the S3 Video). The growth of the tumor cells showed in Fig 10b, allowed us to compute the specific growth rate of the tumor (Eq 3), which give us *α* = 0.053%/day. By using the Gompertz Model (Eq 2), we can predict the tumor growth kinetics. We have considered the carrying constant *K* as a 50 mm tumor, based on the biggest tumor size in the *Milan criteria* [129]. The initial volume would be the tumor size at the end of the simulation which was *V*_0_ = 0.0028 mm^3^, and the constant *α* is the specific growth rate. The prediction of Gompertz model is shown in Fig 10c as a blue dotted line. If we consider a detection size of 5 mm, the recurrence of the modeled tumor could be detected around the 95^*th*^ day (as shown in the inset of Fig 10c). In Fig 10b, shaded regions represent the standard deviations of 40 simulations for liver regeneration and for tumor growth, while in Fig 10c shaded regions represent the Gompertz model calculated based on the standard deviation values. This is in qualitative agreement with the clinical observations presented in Refs. [130–132], in which the earliest time from surgery to first recurrence was 45 days. It is considered an early recurrence.

**Fig 11:**
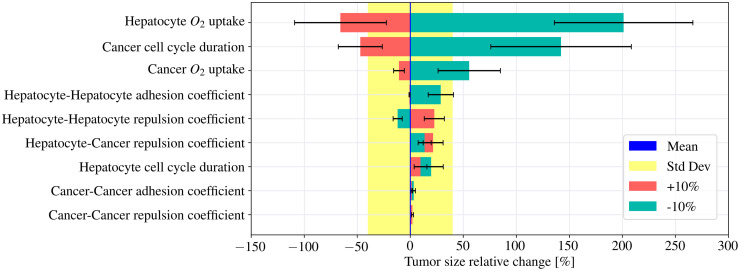
Sensitivity study. Relative change of the tumor final size, based on the variation of the parameters that feed our model. The blue line represents the mean tumor size, the yellow shaded region represents the standard deviation of the mean value based on the stochasticity of the model. Red bars and blue bars represent the mean value of the tumor growth when the parameter original value is increased and decreased by 10% respectively and the error bars represent the standard deviation. The three parameters most likely to make an impact on the tumor growth are: the hepatocyte oxygen uptake constant, cancer cells cycle duration and cancer cells oxygen uptake constant.

Our model has thus proven its ability to estimate the growth kinetics of the tumor based on its early stage growth. It could well turn into a useful tool to determine the optimal follow-up interval after the PH. Currently, the American Association for the Study of Liver Diseases, the European Association for the Study of Liver, and the Asian Pacific Association for the Study of the Liver recommend that HCC screening must be conducted at 6-month intervals [133]. But a consensus interval for recurrence after surgical resection has not been established. Moreover, this recommendation is based on the assumption that the HCC growth rate is similar in every patient. However, tumor growth in general is strongly affected by the microenvironment [134], and HCC growth rate is likely affected by host factors such as age, sex, preexisting diseases, etc., as well as by tumor factors, such as initial average HCC diameter, tumor multiplicity, etc. [135]. Therefore, we used our model to establish a proof of concept of how knowing different parameters that determine tumor development can predict such behavior. We have performed an exploratory sensitivity analysis by varying ±10% the input variables that feed our model: oxygen uptake of tumor cells and hepatocytes, hepatocytes and cancer cell cycles duration, repulsion and adhesion coefficients between cancer cells and hepatocytes. Fig 11 shows the tumor size relative change based on the variation of those parameters. The blue line represents the mean tumor size, the yellow shaded region represents the standard deviation of the mean value based on the stochasticity of the model. Red bars and blue bars represent tumor growth when the parameter original value is increased and decreased by 10% respectively. The parameters whose bars fall into the yellow zone do not modify the tumor growth, hence, according to our model only three parameters mostly control tumor growth. The largest influence is exerted by the hepatocyte oxygen uptake constant. When we increase this constant by 10%, the hepatocytes need more oxygen to keep metabolically active. That causes a reduction in the oxygen concentration in the microenvironment which leads to a reduction in the cancer cell cycle entry. It shrinks the tumor final size by 66%. On the other hand, when the hepatocyte oxygen uptake constant is decreased by 10%, there is more oxygen available in the microenvironment, and the final tumor size will be increased by 200%. Reducing the same constant in cancer cells, also impacts in the final tumor size, however, not as significantly as in the previous instance. The second most influential parameter is the cancer cell cycle duration. A 10% reduction leads to an increase of the tumor final size by 140%. Conversely, increasing this parameter by the same amount will shrink the tumor size a 47%.

In clinical practice, those parameters can be associated to patient specific factors. It has been reported that cell division rates decrease with age [136, 137], so that younger individuals have a higher fraction of dividing cells than older individuals. In fact, although the removal of up to 75% of the total liver volume is feasible in a young patient (≤40 years of age) with normal hepatic parenchyma, it is suggested that resection must be more conservative in elderly patients (e.g., ≥70 years of age) [68]. Other studies show that regenerating liver consumed an increased amount of oxygen, especially during DNA synthesis after hepatectomy [117, 138], that is why dynamic assessment of preoperative exercise capacity may be a useful predictor of short- and long-term postoperative prognosis [139]. Even though at its current stage, our model can qualitatively and quantitatively capture characteristics of the processes of liver regeneration and hepatocellular carcinoma recurrence, the data from markers that assess the patient’s liver overall health could be used to feed the algorithm with specific patient factors, and in this way the model would easily transform into a workbench for hypotheses testing.

## 4 Discussion

In this study, we developed a 3-D off-lattice agent-based model to simulate large multi-cellular systems with the ability to test tissue-scale effects resulting from cell-scale interactions. One of the main characteristics of this type of models is their high predictive power. Obviously, in this context, a pre-requisite is a careful calibration and validation of the ABM. This means, one should first tune the model with real data until the known tissue behavior is reproduced. Since the liver has a remarkable capacity to regenerate, liver regeneration after partial hepatectomy has long been an excellent testing ground for modeling tissue regeneration. Moreover, although the liver consists of various types of cells, hepatocytes account for about 80% of liver weight and about 70% of all liver cells, which simplifies some of physiological aspects the model has to address. One can focus only on hepatocytes in order to study the relation of organ size with number and size of cells, and this makes them the ideal candidates to be the agents of our model. On the other hand, taking into consideration that the liver has a double supply of oxygen through the portal vein and hepatic artery, intrahepatic blood flow is highly regulated both by the disposition of its anatomical unit, which is the lobule, as well as by the interaction of its components with the extracellular matrix. Dirichlet nodes turn out to be the perfect computational construct to represent these blood vessels’ architecture and function, and thus add further realism to the oxygen diffusion within the model organ.

It is to be stressed that the precise mechanisms of initiating, promoting and terminating regenerative responses remain to some extent unknown. Here we have proposed a computational regulatory mechanism based on a substrate that diffuses in the cell microenvironment, substrate here denoted as growth factor. We assume that when the liver is intact, the microenvironment is in homeostasis and, although they are metabolically active, hepatocytes remain dormant in the cell cycle. When a partial hepatectomy takes place, each cell secretes a constant amount of growth factor and, depending on its concentration, they will undergo either hypertrophy or proliferation, as observed by Miyaoka et al. With all that information, we have performed two experiments to calibrate and validate our model. First we studied the liver regeneration process after a 30% PH on an normal hepatic parenchyma. In this context, the liver recovers its volume due to the hypertrophy of the hepatocytes, no proliferation is observed. Secondly, we studied the liver regeneration process after a 70% PH. Here hepatocytes enlarge their volume but also proliferate. The outcomes of our simulations, which are shown in section 3.1, are in agreement with the observations reported by Miyaoka et al.

We consider important to stress the fact that despite the major simplifications that our ABM presents, its outcomes show that the overall functionality of the model is preserved. Since the liver does not recover its original shape, the reduced spherical model served properly to reproduce the liver regeneration dynamics. Moreover, since the grid we used to simulate the liver is orders of magnitude smaller than an actual liver, the region could be considered as representative of sub-regions of the liver, avoiding architectural issues. On the other hand, our results show that by tuning the effective oxygen diffusion and decay constants, we can reproduce the effect of sinusoids in the liver lobules. Although our model does not place limitations on the liver size and shape, and allow us to introduce some inhomogeneities in the microenvironment in order to simulate the sinusoids, we believe that it will increase the computational burden of the simulations and it will not make much of a difference to the results. The comparison between *in silico* and *in vitro* systems shows that even a model based on simple rules governing cell cycle, intercellular bonding and basic physical relationships between neighboring cells can successfully reproduce the behavior of a real biological system.

Once the model is calibrated, it can be used to study the emergent behavior of the tissue in different scenarios. First, we implemented our model to test another degree of PH. Since 50% PH has now been successfully and frequently used for surgical removal of liver associate tumors, we have performed an *in silico* 50% PH. Here, similar to the 70% PH, hepatocytes enlarge their volume and also proliferate but with a reduced replication rate. We adjusted the results of liver regeneration time and percentage of proliferation based on the degree of PH of our simulated PH, to get an approximation of the liver behavior under other resection degrees.

Second, since the PH is the first line of treatment for patients with hepatocellular carcinoma in stage 0- A of BCLC staging system without clinically significant portal hypertension, we used our model to predict the potential recurrence of the tumor in the remnant liver after a 70% PH. The outcome of our simulations is in accordance with clinical observations, which comes to reinforce our confidence in the applicability of this approach in other scenarios. In that context and based on the fact that HCC growth rate is likely affected by host factors, we have performed an exploratory sensitivity analysis by varying ±10% the input variables that feed our model. We found that there are three parameters that according to our model most likely make an impact on the tumor growth: the hepatocyte oxygen uptake constant, cancer cells cycle duration and cancer cells oxygen uptake constant. We take this as a proof of concept of how knowing different parameters that determine tumor development predict such behavior in this 3-D off-lattice agent-based model. We have also studied the tumor growth rate based on the initial position of the the residual tumor clone. We observed that tumor volume vary depending on the location of the residual tumor clone. It grows faster when is located at the periphery of the liver. This result suggest that, although the geometry of the liver does not influence when study liver regeneration, it might make an impact on the HCC recurrence.

It is important to mention that even though at its current stage, our model can qualitatively and quantitatively capture characteristics of the processes of liver regeneration and hepatocellular carcinoma recurrence, it is not calibrated to any particular type of patient specific parameters (age, sex, pre-existing conditions, …). This is obviously a handicap for the model’s direct application in clinical practice. However, the algorithm can be fed with specific patient factors, and in this way the model would easily transform into a workbench for hypotheses testing. The data required for model calibration should include tissue biopsies, as well as data from markers that assess the patient’s liver overall health. Such data set can be used to both quantify different cell types, and record their spatial arrangement. Half the images can be considered training data and be used to calibrate the ABM, while the other half can serve as testing data reserved to check if the model predicts tissue behavior with reasonable accuracy. In a way, this procedure resembles the training of a neural network.

Some limitations should be stressed in our current model. Firstly, the sample sizes are considerable smaller than those expected in real situations. As mentioned, in order to accommodate the simulations to temporal and spatial scales accessible to regular computer facilities, the lattice we used to model liver regeneration is 1 mm^3^ in volume, far too small when compared with actual human liver size. Even if the region could be considered as representative of sub-regions of the liver, is obvious that important size effects might be left out. This challenge can be tackled resorting to new programming methodologies based on massively parallel computational approaches for multi-core and multi-tensor core devices such as the modern general purposes graphic processing units. Research along these lines is currently in progress.

A second limitation at the cellular-tissue scale is that some important cell types found in the liver, such as, biliary epithelial cells, stellate cells and Kupffer cells, are not included in the current model. Even though hepatocytes are responsible for most of the metabolic and synthetic functions of the liver, a future improvement could be the implementation of the interaction between different cell types and explore how it affects liver regeneration processes. A substantial amount of physiological research would be required as a prior step to the implementation of this model upgrade.

Finally, a few words concerning our model’s implementation. We have been particularly careful to construct a software platform in a modular and extensible fashion. The aforementioned modules can be replaced with ones with more fine-grained versions as discussed, so that more specific details can be incorporated (as properties) and new processes as well (as methods) with different degrees of detail. Even though our model is not a 1 : 1 *in silico* copy of the liver and, therefore, it can not accurately describe in full detail the complex biology of liver regeneration and HCC recurrence, it could serve as a tool to test different hypotheses, as well as for testing and analyzing possible outcomes using multiple plausible parameter combinations. We are confident that once the goal of implementing patient specific factors is reached and the model undergoes a rigorous calibration and validation, it could be used as a platform for *in silico* conducting virtual clinical trials.

## Supporting information

S1 Text Supplementary information. Extensive supplementary information including full mathematical model details, supporting literature and reference parameter values for main examples.
(PDF)

S1 Video Liver regeneration after 30% PHx. 3D simulation of the liver regeneration after a 30% partial hepatectomy. Video available at https://youtu.be/7URQweHJ6Tk.
(AVI)

S2 Video Liver regeneration after 70% PHx. 3D simulation of the liver regeneration after a 70% partial hepatectomy. Video available at https://youtu.be/xFtj6ZjrsKk.
(AVI)

S3 Video Hepatocellular carcinoma recurrence after a 70% PHx. 3D simulation of the hepatocellular carcinoma recurrence after a 70% partial hepatectomy. Video available at https://youtu.be/znVfIxt_Gno.
(AVI)

## References

[1] Al-Tameemi M, Chaplain M, d’Onofrio A. Evasion of tumours from the control of the immune system: consequences of brief encounters. Biology direct. 2012;7(1):1–22. doi: 10.1186/1745-6150-7-31 23009638 PMC3582466

[2] Kirschner D, Panetta JC. Modeling immunotherapy of the tumor–immune interaction. Journal of mathematical biology. 1998;37(3):235–252. doi: 10.1007/s002850050127 9785481

[3] Li X, Xu JX. A mathematical prognosis model for pancreatic cancer patients receiving immunotherapy. Journal of theoretical biology. 2016;406:42–51. doi: 10.1016/j.jtbi.2016.06.021 27338302

[4] Mahasa KJ, Ouifki R, Eladdadi A, de Pillis L. Mathematical model of tumor–immune surveillance. Journal of theoretical biology. 2016;404:312–330. doi: 10.1016/j.jtbi.2016.06.012 27317864

[5] Mamat M, Subiyanto KA, Kartono A. Mathematical model of cancer treatments using immunotherapy, chemotherapy and biochemotherapy. Appl Math Sci. 2013;7(5):247–261.

[6] Palsson S, Hickling TP, Bradshaw-Pierce EL, Zager M, Jooss K, O’Brien PJ, et al. The development of a fully-integrated immune response model (FIRM) simulator of the immune response through integration of multiple subset models. BMC systems biology. 2013;7(1):1–19. doi: 10.1186/1752-0509-7-95 24074340 PMC3853972

[7] de Pillis LG, Radunskaya A. A mathematical model of immune response to tumor invasion. In: Computational fluid and solid mechanics 2003. Elsevier; 2003. p. 1661–1668.

[8] de Pillis LG, Radunskaya AE, Wiseman CL. A validated mathematical model of cell-mediated immune response to tumor growth. Cancer research. 2005;65(17):7950–7958. doi: 10.1158/0008-5472.CAN-05-0564 16140967

[9] Serre R, Benzekry S, Padovani L, Meille C, André N, Ciccolini J, et al. Mathematical modeling of cancer immunotherapy and its synergy with radiotherapy. Cancer research. 2016;76(17):4931–4940. doi: 10.1158/0008-5472.CAN-15-3567 27302167

[10] Soto-Ortiz L, Finley SD. A cancer treatment based on synergy between anti-angiogenic and immune cell therapies. Journal of theoretical biology. 2016;394:197–211. doi: 10.1016/j.jtbi.2016.01.026 26826488 PMC7467856

[11] Agur Z, Halevi-Tobias K, Kogan Y, Shlagman O. Employing dynamical computational models for personalizing cancer immunotherapy. Expert opinion on biological therapy. 2016;16(11):1373–1385. doi: 10.1080/14712598.2016.1223622 27564141

[12] DePillis L, Eladdadi A, Radunskaya A. Modeling cancer-immune responses to therapy. Journal of pharmacokinetics and pharmacodynamics. 2014;41(5):461–478. doi: 10.1007/s10928-014-9386-9 25281420

[13] Alizadeh AA, Aranda V, Bardelli A, Blanpain C, Bock C, Borowski C, et al. Toward understanding and exploiting tumor heterogeneity. Nature medicine. 2015;21(8):846–853. doi: 10.1038/nm.3915 26248267 PMC4785013

[14] Beca F, Polyak K. Intratumor heterogeneity in breast cancer. Novel biomarkers in the continuum of breast cancer. 2016; p. 169–189. doi: 10.1007/978-3-319-22909-6_7 26987535

[15] F RS, Volker G. Agent-Based and Individual-Based Modeling: A Practical Introduction, Second Edition. Princeton University Press; 2019.

[16] Abar S, Theodoropoulos GK, Lemarinier P, O’Hare GMP. Agent Based Modelling and Simulation tools: A review of the state-of-art software. Computer Science Review. 2017;24:13–33. doi: 10.1016/j.cosrev.2017.03.001

[17] Macklin P, Edgerton ME, Thompson AM, Cristini V. Patient-calibrated agent-based modelling of ductal carcinoma in situ (DCIS): from microscopic measurements to macroscopic predictions of clinical progression. Journal of theoretical biology. 2012;301:122–140. doi: 10.1016/j.jtbi.2012.02.002 22342935 PMC3322268

[18] Salgado M, Gilbert N. Agent based modelling. In: Handbook of quantitative methods for educational research. Brill; 2013. p. 247–265.

[19] Enderling H, Wolkenhauer O. Are all models wrong? Computational and systems oncology. 2020;1(1). doi: 10.1002/cso2.1008 33585835 PMC7880041

[20] Aldridge BB, Burke JM, Lauffenburger DA, Sorger PK. Physicochemical modelling of cell signalling pathways. Nature cell biology. 2006;8(11):1195–1203. doi: 10.1038/ncb1497 17060902

[21] Agur Z, Kogan Y, Levi L, Harrison H, Lamb R, Kirnasovsky OU, et al. Disruption of a Quorum Sensing mechanism triggers tumorigenesis: a simple discrete model corroborated by experiments in mammary cancer stem cells. Biology direct. 2010;5(1):1–11. doi: 10.1186/1745-6150-5-20 20406437 PMC2874533

[22] Anderson AR, Chaplain MAJ. Continuous and discrete mathematical models of tumor-induced angiogenesis. Bulletin of mathematical biology. 1998;60(5):857–899. doi: 10.1006/bulm.1998.0042 9739618

[23] Enderling H, Anderson AR, Chaplain MA, Beheshti A, Hlatky L, Hahnfeldt P. Paradoxical dependencies of tumor dormancy and progression on basic cell kinetics. Cancer research. 2009;69(22):8814–8821. doi: 10.1158/0008-5472.CAN-09-2115 19887613

[24] Enderling H, Hlatky L, Hahnfeldt P. Migration rules: tumours are conglomerates of self-metastases. British journal of cancer. 2009;100(12):1917–1925. doi: 10.1038/sj.bjc.6605071 19455139 PMC2714240

[25] Enderling H, Hlatky L, Hahnfeldt P. The promoting role of a tumour-secreted chemorepellent in self-metastatic tumour progression. Mathematical medicine and biology: a journal of the IMA. 2012;29(1):21–29. doi: 10.1093/imammb/dqq015 20929852

[26] Gerlee P, Anderson AR. A hybrid cellular automaton model of clonal evolution in cancer: the emergence of the glycolytic phenotype. Journal of theoretical biology. 2008;250(4):705–722. doi: 10.1016/j.jtbi.2007.10.038 18068192 PMC2846650

[27] Gerlee P, Anderson A. Evolution of cell motility in an individual-based model of tumour growth. Journal of theoretical biology. 2009;259(1):67–83. doi: 10.1016/j.jtbi.2009.03.005 19285513 PMC2706369

[28] Norton KA, Popel AS. An agent-based model of cancer stem cell initiated avascular tumour growth and metastasis: the effect of seeding frequency and location. Journal of The Royal Society Interface. 2014;11(100):20140640. doi: 10.1098/rsif.2014.0640 25185580 PMC4191089

[29] Norton KA, Popel AS. Effects of endothelial cell proliferation and migration rates in a computational model of sprouting angiogenesis. Scientific reports. 2016;6(1):1–10. doi: 10.1038/srep36992 27841344 PMC5107954

[30] Smallbone K, Gatenby RA, Gillies RJ, Maini PK, Gavaghan DJ. Metabolic changes during carcinogenesis: potential impact on invasiveness. Journal of theoretical biology. 2007;244(4):703–713. doi: 10.1016/j.jtbi.2006.09.010 17055536

[31] Drasdo D, Höhme S. A single-cell-based model of tumor growth in vitro: monolayers and spheroids. Physical biology. 2005;2(3):133. doi: 10.1088/1478-3975/2/3/001 16224119

[32] Norton KA, Wininger M, Bhanot G, Ganesan S, Barnard N, Shinbrot T. A 2D mechanistic model of breast ductal carcinoma in situ (DCIS) morphology and progression. Journal of theoretical biology. 2010;263(4):393–406. doi: 10.1016/j.jtbi.2009.11.024 20006623 PMC2839055

[33] Ghaffarizadeh A, Heiland R, Friedman SH, Mumenthaler SM, Macklin P. PhysiCell: An open source physics-based cell simulator for 3-D multicellular systems. PLOS Computational Biology. 2018;14(2):1–31. doi: 10.1371/journal.pcbi.1005991 29474446 PMC5841829

[34] Gong C, Milberg O, Wang B, Vicini P, Narwal R, Roskos L, et al. A computational multiscale agent-based model for simulating spatio-temporal tumour immune response to PD1 and PDL1 inhibition. Journal of The Royal Society Interface. 2017;14(134):20170320. doi: 10.1098/rsif.2017.0320 28931635 PMC5636269

[35] Norton KA, Gong C, Jamalian S, Popel AS. Multiscale agent-based and hybrid modeling of the tumor immune microenvironment. Processes. 2019;7(1):37. doi: 10.3390/pr7010037 30701168 PMC6349239

[36] Taub R. Liver regeneration: from myth to mechanism. Nature reviews Molecular cell biology. 2004;5(10):836–847. doi: 10.1038/nrm1489 15459664

[37] Michalopoulos GK. Liver regeneration. Journal of cellular physiology. 2007;213(2):286–300. doi: 10.1002/jcp.21172 17559071 PMC2701258

[38] Si-Tayeb K, Lemaigre FP, Duncan SA. Organogenesis and development of the liver. Developmental cell. 2010;18(2):175–189. doi: 10.1016/j.devcel.2010.01.011 20159590

[39] Alison M, Islam S, Lim S. Stem cells in liver regeneration, fibrosis and cancer: the good, the bad and the ugly. The Journal of Pathology: A Journal of the Pathological Society of Great Britain and Ireland. 2009;217(2):282–298. doi: 10.1002/path.2453 18991329

[40] Palmes D, Spiegel HU. Animal models of liver regeneration. Biomaterials. 2004;25(9):1601–1611. doi: 10.1016/S0142-9612(03)00508-8 14697862

[41] Haga S, Ogawa W, Inoue H, Terui K, Ogino T, Igarashi R, et al. Compensatory recovery of liver mass by Akt-mediated hepatocellular hypertrophy in liver-specific STAT3-deficient mice. Journal of hepatology. 2005;43(5):799–807. doi: 10.1016/j.jhep.2005.03.027 16083985

[42] Haga S, Ozaki M, Inoue H, Okamoto Y, Ogawa W, Takeda K, et al. The survival pathways phosphatidylinositol-3 kinase (PI3-K)/phosphoinositide-dependent protein kinase 1 (PDK1)/Akt modulate liver regeneration through hepatocyte size rather than proliferation. Hepatology. 2009;49(1):204–214. doi: 10.1002/hep.22583 19065678

[43] Minamishima YA, Nakayama K, Nakayama KI. Recovery of liver mass without proliferation of hepatocytes after partial hepatectomy in Skp2-deficient mice. Cancer Research. 2002;62(4):995–999. 11861371

[44] Miyaoka Y, Miyajima A. To divide or not to divide: revisiting liver regeneration. Cell division. 2013;8(1):1–12. doi: 10.1186/1747-1028-8-8 23786799 PMC3695844

[45] Miyaoka Y, Ebato K, Kato H, Arakawa S, Shimizu S, Miyajima A. Hypertrophy and Unconventional Cell Division of Hepatocytes Underlie Liver Regeneration. Current Biology. 2012;22(13):1166–1175. doi: 10.1016/j.cub.2012.05.016 22658593

[46] Rahbari NN, Garden OJ, Padbury R, Brooke-Smith M, Crawford M, Adam R, et al. Posthepatectomy liver failure: a definition and grading by the International Study Group of Liver Surgery (ISGLS). Surgery. 2011;149(5):713–724. doi: 10.1016/j.surg.2010.10.001 21236455

[47] Fukushima K, Fukumoto T, Kuramitsu K, Kido M, Takebe A, Tanaka M, et al. Assessment of ISGLS definition of posthepatectomy liver failure and its effect on outcome in patients with hepatocellular carcinoma. Journal of Gastrointestinal Surgery. 2014;18(4):729–736. doi: 10.1007/s11605-013-2423-y 24297653

[48] Kuramitsu K, Fukumoto T, Kobayashi T, Fukushima K, Okimoto S, Iwasaki T, et al. The incidence of posthepatectomy liver failure defined by the international study group of liver surgery among living donors. Journal of Gastrointestinal Surgery. 2016;20(4):757–764. doi: 10.1007/s11605-016-3080-8 26791388

[49] Narita M, Oussoultzoglou E, Bachellier P, Jaeck D, Uemoto S. Post-hepatectomy liver failure in patients with colorectal liver metastases. Surgery today. 2015;45(10):1218–1226. doi: 10.1007/s00595-015-1113-7 25628126

[50] Zorzi D, Laurent A, Pawlik T, Lauwers G, Vauthey J, Abdalla E. Chemotherapy-associated hepatotoxicity and surgery for colorectal liver metastases. Journal of British Surgery. 2007;94(3):274–286. doi: 10.1002/bjs.5719 17315288

[51] Ribero D, Chun YS, Vauthey JN. Standardized liver volumetry for portal vein embolization. In: Seminars in interventional radiology. vol. 25. Thieme Medical Publishers; 2008. p. 104–109. 21326551 10.1055/s-2008-1076681 PMC3036478

[52] Riediger C, Mueller MW, Geismann F, Lehmann A, Schuster T, Michalski CW, et al. Comparative analysis of different transection techniques in minor and major hepatic resections: a prospective cohort study. International Journal of Surgery. 2013;11(9):826–833. doi: 10.1016/j.ijsu.2013.08.010 23994002

[53] Jin S, Fu Q, Wuyun G, Wuyun T. Management of post-hepatectomy complications. World journal of gastroenterology: WJG. 2013;19(44):7983. doi: 10.3748/wjg.v19.i44.7983 24307791 PMC3848145

[54] Kauffmann R, Fong Y. Post-hepatectomy liver failure. Hepatobiliary surgery and nutrition. 2014;3(5):238. doi: 10.3978/j.issn.2304-3881.2014.09.01 25392835 PMC4207837

[55] D’Onofrio M, De Robertis R, Demozzi E, Crosara S, Canestrini S, Mucelli RP. Liver volumetry: Is imaging reliable? Personal experience and review of the literature. World journal of radiology. 2014;6(4):62. doi: 10.4329/wjr.v6.i4.62 24778768 PMC4000610

[56] Llovet J, Kelley R, Villanueva A, Singal A, Pikarsky E, Roayaie S, et al. Hepatocellular carcinoma. Nature Reviews Disease Primers. 2021;7(1):6. doi: 10.1038/s41572-020-00240-3 33479224 PMC13537433

[57] Reig M, Forner A, Rimola J, Ferrer-Fábrega J, Burrel M, Garcia-Criado Á, et al. BCLC strategy for prognosis prediction and treatment recommendation: The 2022 update. Journal of Hepatology. 2021. doi: 10.1016/j.jhep.2021.11.018 34801630 PMC8866082

[58] Tobe T, Uchino J, Endo Y, Oto M, Okamoto E, Kojiro M, et al. Predictive factors for long term prognosis after partial hepatectomy for patients with hepatocellular carcinoma in Japan. The Liver Cancer Study Group of Japan. Cancer. 1994;74(10):2772–2780. doi: 10.1002/1097-0142(19941115)74:10<2772::AID-CNCR2820741006>3.0.CO;2-V 7954236

[59] Ikai I, Arii S, Kojiro M, Ichida T, Makuuchi M, Matsuyama Y, et al. Reevaluation of prognostic factors for survival after liver resection in patients with hepatocellular carcinoma in a Japanese nationwide survey. Cancer. 2004;101(4):796–802. doi: 10.1002/cncr.20426 15305412

[60] Tampaki M, Papatheodoridis GV, Cholongitas E. Intrahepatic recurrence of hepatocellular carcinoma after resection: an update. Clinical Journal of Gastroenterology. 2021;14(3):699–713. doi: 10.1007/s12328-021-01394-7 33774785

[61] Huang R, Zhang X, Gracia-Sancho J, Xie WF. Liver regeneration: cellular origin and molecular mechanisms. Liver International. 2022. doi: 10.1111/liv.15174 35107210

[62] Michalopoulos GK, Bhushan B. Liver regeneration: biological and pathological mechanisms and implications. Nature reviews Gastroenterology & hepatology. 2021;18(1):40–55. doi: 10.1038/s41575-020-0342-4 32764740

[63] Yagi S, Hirata M, Miyachi Y, Uemoto S. Liver regeneration after hepatectomy and partial liver transplantation. International Journal of Molecular Sciences. 2020;21(21):8414. doi: 10.3390/ijms21218414 33182515 PMC7665117

[64] Campana L, Esser H, Huch M, Forbes S. Liver regeneration and inflammation: From fundamental science to clinical applications. Nature Reviews Molecular Cell Biology. 2021;22(9):608–624. doi: 10.1038/s41580-021-00373-7 34079104

[65] Boyer TD, Lindor KD. Zakim and Boyer’s hepatology: A textbook of liver disease e-book. Elsevier Health Sciences; 2016.

[66] Pawlina W, Ross MH. Histology: a text and atlas: with correlated cell and molecular biology. Lippincott Williams & Wilkins; 2018.

[67] Tsomaia K, Patarashvili L, Karumidze N, Bebiashvili I, Azmaipharashvili E, Modebadze I, et al. Liver structural transformation after partial hepatectomy and repeated partial hepatectomy in rats: A renewed view on liver regeneration. World Journal of Gastroenterology. 2020;26(27):3899. doi: 10.3748/wjg.v26.i27.3899 32774065 PMC7385567

[68] Clavien PA, Petrowsky H, DeOliveira ML, Graf R. Strategies for safer liver surgery and partial liver transplantation. New England Journal of Medicine. 2007;356(15):1545–1559. doi: 10.1056/NEJMra065156 17429086

[69] Abdeldayem H. General Introduction: Advances in Hepatic Surgery. London: InTech. 2013.

[70] Nilsson J, Eriksson S, Blind PJ, Rissler P, Sturesson C. Microcirculation changes during liver resection—a clinical study. Microvascular research. 2014;94:47–51. doi: 10.1016/j.mvr.2014.05.002 24840670

[71] Christ B, Dahmen U, Herrmann KH, König M, Reichenbach JR, Ricken T, et al. Computational modeling in liver surgery. Frontiers in Physiology. 2017;8:906. doi: 10.3389/fphys.2017.00906 29249974 PMC5715340

[72] van Dam RM, Lodewick TM, van den Broek MA, de Jong MC, Greve JW, Jansen RL, et al. Outcomes of extended versus limited indications for patients undergoing a liver resection for colorectal cancer liver metastases. HPB. 2014;16(6):550–559. doi: 10.1111/hpb.12181 24246003 PMC4048077

[73] Kang KJ, Ahn KS. Anatomical resection of hepatocellular carcinoma: A critical review of the procedure and its benefits on survival. World journal of gastroenterology. 2017;23(7):1139. doi: 10.3748/wjg.v23.i7.1139 28275294 PMC5323439

[74] Michalopoulos GK, DeFrances M. Liver regeneration. Regenerative Medicine I. 2005; p. 101–134. doi: 10.1007/b99968 15791946

[75] Fausto N, Campbell JS, Riehle KJ. Liver regeneration. Hepatology. 2006;43(S1):S45–S53. doi: 10.1002/hep.20969 16447274

[76] Fausto N. Liver regeneration: from laboratory to clinic. Liver transplantation. 2001;7(10):835–844. doi: 10.1053/jlts.2001.27865 11679980

[77] Kogure K, Ishizaki M, Nemoto M, Kuwano H, Makuuchi M. A comparative study of the anatomy of rat and human livers. Journal of hepato-biliary-pancreatic surgery. 1999;6(2):171–175. doi: 10.1007/s005340050101 10398905

[78] Stukowski A. Visualization and analysis of atomistic simulation data with OVITO-the Open Visualization Tool. Modeling and simulation in materials science and engineering. 2010;18(1). doi: 10.1088/0965-0393/18/1/015012

[79] Ahrens J, Geveci B, Law C. Paraview: An end-user tool for large data visualization. The visualization handbook. 2005;717(8). doi: 10.1016/B978-012387582-2/50038-1

[80] Hunter JD. Matplotlib: A 2D graphics environment. Computing in Science & Engineering. 2007;9(3):90–95. doi: 10.1109/MCSE.2007.55

[81] Marchuk GI. Splitting and alternating direction methods. vol. 1 of Handbook of Numerical Analysis. Elsevier; 1990. p. 197–462.

[82] Eymard R, Gallouët T, Herbin R. Finite Volume Methods. In: Lions JL, Ciarlet P, editors. Solution of Equation in ℝn (Part 3), Techniques of Scientific Computing (Part 3). vol. 7 of Handbook of Numerical Analysis. Elsevier; 2000. p. 713–1020. Available from: https://hal.archives-ouvertes.fr/hal-02100732.

[83] Yanenko NN. In: Holt M, editor. Simple Schemes in Fractional Steps for the Integration of Parabolic Equations. Berlin, Heidelberg: Springer Berlin Heidelberg; 1971. p. 17–41.

[84] Thomas LH. Elliptic problems in linear difference equations over a network. Watson Sci Comput Lab Rept, Columbia University, New York. 1949;1:71.

[85] Ghaffarizadeh A, Friedman SH, Macklin P. BioFVM: an efficient, parallelized diffusive transport solver for 3-D biological simulations. Bioinformatics. 2015;32(8):1256–1258. doi: 10.1093/bioinformatics/btv730 26656933 PMC4824128

[86] Sütterlin T, Tsingos E, Bensaci J, Stamatas GN, Grabe N. A 3D self-organizing multicellular epidermis model of barrier formation and hydration with realistic cell morphology based on EPISIM. Scientific reports. 2017;7(1):1–11. doi: 10.1038/srep43472 28262741 PMC5338006

[87] Mirams GR, Arthurs CJ, Bernabeu MO, Bordas R, Cooper J, Corrias A, et al. Chaste: an open source C++ library for computational physiology and biology. PLoS computational biology. 2013;9(3):e1002970. doi: 10.1371/journal.pcbi.1002970 23516352 PMC3597547

[88] Bruno S, Darzynkiewicz Z. Cell cycle dependent expression and stability of the nuclear protein detected by Ki-67 antibody in HL-60 cells. Cell Proliferation. 1992;25(1):31–40. doi: 10.1111/j.1365-2184.1992.tb01435.x 1540682

[89] Urruticoechea A, Smith IE, Dowsett M. Proliferation marker Ki-67 in early breast cancer. Journal of clinical oncology. 2005;23(28):7212–7220. doi: 10.1200/JCO.2005.07.501 16192605

[90] Aoyagi M, Yamamoto M, Wakimoto H, Azuma H, Hirakawa K, Yamamoto K. Immunohistochemical detection of Ki-67 in replicative smooth muscle cells of rabbit carotid arteries after balloon denudation. Stroke. 1995;26(12):2328–2332. doi: 10.1161/01.STR.26.12.2328 7491659

[91] Larsson S, Ryden T, Holst U, Oredsson S, Johansson M. Estimating the variation in S phase duration from flow cytometric histograms. Mathematical Biosciences. 2008;213(1):40–49. doi: 10.1016/j.mbs.2008.01.009 18433802

[92] Lin W, Arthur G. Phospholipids are synthesized in the G2/M phase of the cell cycle. The International Journal of Biochemistry & Cell Biology. 2007;39(3):597–605. doi: 10.1016/j.biocel.2006.10.011 17113814

[93] Purcell EM. Life at low Reynolds number. American Journal of Physics. 2000;45:3–11. doi: 10.1119/1.10903

[94] Griffiths D, Higham DJ. Numerical Methods for Ordinary Differential Equations: Initial Value Problems. Springer Undergraduate Mathematics Series. Springer-Verlag; 2010.

[95] Hoffmann K, Nagel AJ, Tanabe K, Fuchs J, Dehlke K, Ghamarnejad O, et al. Markers of liver regeneration—the role of growth factors and cytokines: a systematic review. BMC surgery. 2020;20(1):1–15. doi: 10.1186/s12893-019-0664-8 32050952 PMC7017496

[96] Laird A, Tyler S, Barton A. Dynamics of tumor growth: comparison of growth rates and extrapolation of growth curve to one cell. Br J Cancer. 1965;19:278–291. doi: 10.1038/bjc.1965.32 14316202 PMC2071357

[97] Chignola R, Foroni RI. Estimating the growth kinetics of experimental tumors from as few as two determinations of tumor size: implications for clinical oncology. IEEE transactions on biomedical engineering. 2005;52(5):808–815. doi: 10.1109/TBME.2005.845219 15887530

[98] Nakasu S, Nakasu Y, Fukami T, Jito J, Nozaki K. Growth curve analysis of asymptomatic and symptomatic meningiomas. Journal of neuro-oncology. 2011;102(2):303–310. doi: 10.1007/s11060-010-0319-1 20686821

[99] Stensjøen AL, Solheim O, Kvistad KA, Håberg AK, Salvesen Ø, Berntsen EM. Growth dynamics of untreated glioblastomas in vivo. Neuro-oncology. 2015;17(10):1402–1411. doi: 10.1093/neuonc/nov029 25758748 PMC4578579

[100] Mehrara E, Forssell-Aronsson E, Ahlman H, Bernhardt P. Quantitative analysis of tumor growth rate and changes in tumor marker level: specific growth rate versus doubling time. Acta Oncologica. 2009;48(4):591–597. doi: 10.1080/02841860802616736 19330565

[101] He Ln, Zhang X, Li H, Chen T, Chen C, Zhou Y, et al. Pre-Treatment Tumor Growth Rate Predicts Clinical Outcomes of Patients With Advanced Non-Small Cell Lung Cancer Undergoing Anti-PD-1/PD-L1 Therapy. Frontiers in oncology. 2021; p. 3041. 10.3389/fonc.2020.621329 PMC7863973 33552993

[102] Nickolls J, Buck I, Garland M, Skadron K. Scalable parallel programming with cuda: Is cuda the parallel programming model that application developers have been waiting for? Queue. 2008;6(2):40–53. doi: 10.1145/1365490.1365500

[103] Rotstein J, Macdonald PDM, Rabes HM, Farber E. Cell Cycle Kinetics of Rat Hepatocytes in Early Putative Preneoplastic Lesions in Hepatocarcinogenesis. Cancer Research. 1984;44(7):2913–2917. 6144384

[104] Taniguchi E, Sakisaka S, Matsuo K, Tanikawa K, Sata M. Expression and role of vascular endothelial growth factor in liver regeneration after partial hepatectomy in rats. Journal of Histochemistry & Cytochemistry. 2001;49(1):121–129. doi: 10.1177/002215540104900112 11118484

[105] Sato T, El-Assal ON, Ono T, Yamanoi A, Dhar DK, Nagasue N. Sinusoidal endothelial cell proliferation and expression of angiopoietin/Tie family in regenerating rat liver. Journal of hepatology. 2001;34(5):690–698. doi: 10.1016/S0168-8278(00)00109-4 11434615

[106] Shimizu H, Miyazaki M, Wakabayashi Y, Mitsuhashi N, Kato A, Ito H, et al. Vascular endothelial growth factor secreted by replicating hepatocytes induces sinusoidal endothelial cell proliferation during regeneration after partial hepatectomy in rats. Journal of hepatology. 2001;34(5):683–689. doi: 10.1016/S0168-8278(00)00055-6 11434614

[107] Leung DW, Cachianes G, Kuang WJ, Goeddel DV, Ferrara N. Vascular endothelial growth factor is a secreted angiogenic mitogen. Science. 1989;246(4935):1306–1309. doi: 10.1126/science.2479986 2479986

[108] Mochida S, Ishikawa K, Toshima K, Inao M, Ikeda H, Matsui A, et al. The mechanisms of hepatic sinusoidal endothelial cell regeneration: a possible communication system associated with vascular endothelial growth factor in liver cells. Journal of gastroenterology and hepatology. 1998;13(S1):S1–S5. doi: 10.1111/jgh.1998.13.s1.1 28976691

[109] Rafii S, Butler JM, Ding BS. Angiocrine functions of organ-specific endothelial cells. Nature. 2016;529(7586):316–325. doi: 10.1038/nature17040 26791722 PMC4878406

[110] Drixler TA, Vogten MJ, Ritchie ED, van Vroonhoven TJ, Gebbink MF, Voest EE, et al. Liver regeneration is an angiogenesis-associated phenomenon. Annals of surgery. 2002;236(6):703. doi: 10.1097/00000658-200212000-00002 12454508 PMC1422636

[111] Fausto N. Liver regeneration. Journal of hepatology. 2000;32:19–31. doi: 10.1016/S0168-8278(00)80412-2 10728791

[112] McCormack L, Petrowsky H, Jochum W, Furrer K, Clavien PA. Hepatic steatosis is a risk factor for postoperative complications after major hepatectomy: a matched case-control study. Annals of surgery. 2007;245(6):923. doi: 10.1097/01.sla.0000251747.80025.b7 17522518 PMC1876953

[113] Clavien PA, Selzner M, Rüdiger HA, Graf R, Kadry Z, Rousson V, et al. A prospective randomized study in 100 consecutive patients undergoing major liver resection with versus without ischemic preconditioning. Annals of surgery. 2003;238(6):843. doi: 10.1097/01.sla.0000098620.27623.7d 14631221 PMC1356166

[114] Rayya F, Harms J, Bartels M, Uhlmann D, Hauss J, Fangmann J. Results of resection and transplantation for hepatocellular carcinoma in cirrhosis and noncirrhosis. In: Transplantation proceedings. vol. 40. Elsevier; 2008. p. 933–935. 18555082 10.1016/j.transproceed.2008.03.045

[115] Haglund UH, Norén A, Urdzik J, Duraj FF. Right hemihepatectomy. Journal of Gastrointestinal Surgery. 2008;12(7):1283–1287. doi: 10.1007/s11605-008-0493-z 18278534

[116] Cao H, Yu J, Xu W, Jia X, Yang J, Pan Q, et al. Proteomic analysis of regenerating mouse liver following 50% partial hepatectomy. Proteome science. 2009;7(1):1–14. doi: 10.1186/1477-5956-7-48 20040084 PMC2813229

[117] Yoshioka S, Miyazaki M, Shimizu H, Ito H, Nakagawa K, Ambiru S, et al. Hepatic venous hemoglobin oxygen saturation predicts regenerative status of remnant liver after partial hepatectomy in rats. Hepatology. 1998;27(5):1349–1353. doi: 10.1002/hep.510270522 9581690

[118] Jung SM, Kim JM, Choi GS, Kwon CHD, Yi NJ, Lee KW, et al. Characteristics of early recurrence after curative liver resection for solitary hepatocellular carcinoma. Journal of Gastrointestinal Surgery. 2019;23(2):304–311. doi: 10.1007/s11605-018-3927-2 30215196

[119] Liang By, Gu J, Xiong M, Zhang El, Zhang Zy, Chen Xp, et al. Tumor size may influence the prognosis of solitary hepatocellular carcinoma patients with cirrhosis and without macrovascular invasion after hepatectomy. Scientific reports. 2021;11(1):1–10. 34381132 10.1038/s41598-021-95835-5 PMC8357938

[120] Cheng Z, Yang P, Qu S, Zhou J, Yang J, Yang X, et al. Risk factors and management for early and late intrahepatic recurrence of solitary hepatocellular carcinoma after curative resection. Hpb. 2015;17(5):422–427. doi: 10.1111/hpb.12367 25421805 PMC4402053

[121] Kim JM, Yi NJ, Kwon CHD, Lee KW, Suh KS, Joh JW. Early disseminated recurrence after liver resection in solitary hepatocellular carcinoma. Annals of Surgical Treatment and Research. 2018;94(3):129–134. doi: 10.4174/astr.2018.94.3.129 29520346 PMC5842084

[122] Han SR, Kim JM, Choi GS, Park JB, Kwon CHD, Kim SJ, et al. Protrusion of hepatocellular carcinoma is a predictor of early recurrence in hepatectomy patients after spontaneous rupture. Annals of Surgical Treatment and Research. 2016;91(1):17–22. doi: 10.4174/astr.2016.91.1.17 27433460 PMC4942534

[123] Kato Y, Okamura Y, Sugiura T, Ito T, Yamamoto Y, Ashida R, et al. The impact of serosal invasion on prognosis after curative hepatectomy for hepatocellular carcinoma: invasion to adjacent organs and rupture of tumor were crucial tumor-related prognostic factors needed for survival. Digestive Surgery. 2018;35(2):155–163. doi: 10.1159/000477270 28787731

[124] Tokumitsu Y, Sakamoto K, Tokuhisa Y, Matsui H, Matsukuma S, Maeda Y, et al. A new prognostic model for hepatocellular carcinoma recurrence after curative hepatectomy. Oncology letters. 2018;15(4):4411–4422. doi: 10.3892/ol.2018.7821 29556288 PMC5844062

[125] Renzulli M, Brandi N, Pecorelli A, Pastore LV, Granito A, Martinese G, et al. Segmental Distribution of Hepatocellular Carcinoma in Cirrhotic Livers. Diagnostics. 2022;12(4):834. doi: 10.3390/diagnostics12040834 35453882 PMC9032124

[126] Li L, Xu L, Zhou S, Wang P, Zhang M, Li B. Tumour site is a risk factor for hepatocellular carcinoma after hepatectomy: a 1: 2 propensity score matching analysis. BMC surgery. 2022;22(1):1–8. doi: 10.1186/s12893-022-01564-5 35313888 PMC8935716

[127] Lai ZC, Liang JY, Chen LD, Wang Z, Ruan SM, Xie XY, et al. Do hepatocellular carcinomas located in subcapsular space or in proximity to vessels increase the rate of local tumor progression? A meta-analysis. Life sciences. 2018;207:381–385. doi: 10.1016/j.lfs.2018.06.016 29924963

[128] Qin W, Wang L, Hu B, Tian H, Xiao C, Luo H, et al. Anatomical sites (Takasaki’s segmentation) predicts the recurrence-free survival of hepatocellular carcinoma. BMC surgery. 2021;21(1):1–10. doi: 10.1186/s12893-021-01275-3 34082743 PMC8176619

[129] Mazzaferro V, Regalia E, Doci R, Andreola S, Pulvirenti A, Bozzetti F, et al. Liver transplantation for the treatment of small hepatocellular carcinomas in patients with cirrhosis. New England Journal of Medicine. 1996;334(11):693–700. doi: 10.1056/NEJM199603143341104 8594428

[130] Yamamoto J, Kosuge T, Takayama T, Shimada K, Yamasaki S, Ozaki H, et al. Recurrence of hepatocellular carcinoma after surgery. Journal of British Surgery. 1996;83(9):1219–1222. doi: 10.1046/j.1365-2168.1996.02342.x 8983610

[131] Ezaki T, Yukaya H, Ogawa Y, Chang Y, Nagasue N. Recurrent form of hepatocellular carcinoma after partial hepatic resection. Hepato-gastroenterology. 1989;36(3):164–167. 2546880

[132] Fan S, Poon R, Yeung C, Lam C, Lo C, Yuen W, et al. Outcome after partial hepatectomy for hepatocellular cancer within the Milan criteria. Journal of British Surgery. 2011;98(9):1292–1300. doi: 10.1002/bjs.7583 21656513

[133] Bruix J, Sherman M. Management of hepatocellular carcinoma: an update. Hepatology (Baltimore, Md). 2011;53(3):1020. doi: 10.1002/hep.24199 21374666 PMC3084991

[134] Kim Y, Stolarska MA, Othmer HG. The role of the microenvironment in tumor growth and invasion. Progress in biophysics and molecular biology. 2011;106(2):353–379. doi: 10.1016/j.pbiomolbio.2011.06.006 21736894 PMC3156881

[135] An C, Choi YA, Choi D, Paik YH, Ahn SH, Kim MJ, et al. Growth rate of early-stage hepatocellular carcinoma in patients with chronic liver disease. Clinical and molecular hepatology. 2015;21(3):279. doi: 10.3350/cmh.2015.21.3.279 26523271 PMC4612289

[136] Tomasetti C, Poling J, Roberts NJ, London NR Jr, Pittman ME, Haffner MC, et al. Cell division rates decrease with age, providing a potential explanation for the age-dependent deceleration in cancer incidence. Proceedings of the National Academy of Sciences. 2019;116(41):20482–20488. doi: 10.1073/pnas.1905722116 31548407 PMC6789572

[137] Michalopoulos GK, DeFrances MC. Liver Regeneration. Science. 1997;276(5309):60–66. doi: 10.1126/science.276.5309.60 9082986

[138] Alexandrino H, Rolo A, Tralhão JG, Castro e Sousa F, Palmeira C. Mitochondria in liver regeneration: energy metabolism and posthepatectomy liver dysfunction. In: Mitochondrial biology and experimental therapeutics. Springer; 2018. p. 127–152.

[139] Kaibori M, Matsui K, Yoshii K, Ishizaki M, Iwasaka J, Miyauchi T, et al. Perioperative exercise capacity in chronic liver injury patients with hepatocellular carcinoma undergoing hepatectomy. PloS one. 2019;14(8):e0221079. doi: 10.1371/journal.pone.0221079 31412075 PMC6693770

