# Supplementary material for: Physics-based tissue simulator to model multicellular systems: A study of liver regeneration and hepatocellular carcinoma recurrence
Source: PLoS Comput Biol. 2023 Mar 6;19(3):e1010920. doi: 10.1371/journal.pcbi.1010920 (PMC10019748; doi:10.1371/journal.pcbi.1010920)
Supplement: S1 Text — Extensive supplementary information including full mathematical model details, supporting literature and reference parameter values for main examples. (PDF) [file pcbi.1010920.s001.pdf]

Supplementary Material for: Physics-based tissue simulator to model multicellular systems: A study of liver regeneration and hepatocellular carcinoma recurrence

Luciana Melina Luque[1*], Carlos Manuel Carlevaro[1,2**], Camilo Julio Llamoza Torres[3], and Enrique Lomba[4]

[1]Instituto de Física de Líquidos y Sistemas Biológicos - CONICET. La Plata, Argentina.

[2]Departamento de Ingeniería Mecánica, Universidad Tecnológica Nacional, Facultad Regional La Plata, La Plata, Argentina.

[3]Hospital Clínico Universitario Virgen de la Arrixaca. Murcia, España

[4]Instituto de Química Física Rocasolano - CSIC. Madrid, España

* lluque@iflysib.unlp.edu.ar

** manuel@iflysib.unlp.edu.ar

Abstract

We present a multiagent-based model that captures the interactions between different types of cells with their microenvironment, and enables the analysis of the emergent global behavior during tissue regeneration and tumor development. Using this model, we are able to reproduce the temporal dynamics of regular healthy cells and cancer cells, as well as the evolution of their three-dimensional spatial distributions. By tuning the system with the characteristics of the individual patients, our model reproduces a variety of spatial patterns of tissue regeneration and tumor growth, resembling those found in clinical imaging or biopsies. In order to calibrate and validate our model we study the process of liver regeneration after surgical hepatectomy in different degrees. In the clinical context, our model is able to predict the recurrence of a hepatocellular carcinoma after a 70% partial hepatectomy. The outcomes of our simulations are in agreement with experimental and clinical observations. By fitting the model parameters to specific patient factors, it might well become a useful platform for hypotheses testing in treatments protocols.

Contents

1 Overall Program **5**

1.1 Input parameters . . . 8

1.2 Output parameters . . . 9

2 Biochemical microenvironment **9**

2.1 Domain discretization and notation . . . 10

2.2 Operator splitting . . . 12

2.3 Finite volume method . . . 12

2.4 Locally one-dimensional method . . . 13

2.5 Thomas algorithm . . . 14

2.6 Boundary conditions and Dirichlet nodes . . . 15

2.7 Test . . . 16

3 Cell **18**

3.1 Proliferation . . . 19

3.2 Cycle . . . 22

3.3 Volume and geometry . . . 23

3.4 Division . . . 26

3.5 Mechanics . . . 27

3.5.1 Numerical implementation . . . 34

3.6 Secretion and uptake . . . 36

3.7 Death . . . 37

3.7.1 Apoptosis . . . 37

3.7.2 Necrosis . . . 38

3.8 Cancer cell . . . 39

3.8.1 Cycle . . . 40

3.8.2 Secretion . . . 41

3.8.3 Death 41

3.8.4 Angiogenesis 41

4 Parameters for the main examples **43**

4.1 General 43

4.2 Liver regeneration 44

4.3 Tumor growth 44

5 References **45**

1 Overall Program

We have developed an off-lattice agent-based model to simulate tissue-scale features that emerge from basic biological and biophysical cell processes. Since it is programmed in the context of an object oriented approach, each cell is an agent implemented as a software object that acts independently, with motion governed by the balance of adhesive, repulsive and motile forces. Each cell also has an independent cell cycle state (including volume changes) and can also progress through apoptotic and necrotic death processes. Moreover, depending on the type of cell, it can also perform biased random migration based on a substrate gradient.

In our model, cell behavior is linked to the values and gradients of diffusing substrates, such as oxygen-dependent cell cycle entry, necrosis and chemotaxis towards signaling factors. To facilitate this, we have modeled a 3-D diffusion solver which solves 3-D diffusion equations for one or many diffusible factors. Cell agents can secrete or uptake from the chemical microenvironment, as well as sample the value or gradient of any or all substrates.

It is cross-platform compatible, *i.e.* it can be compiled and run on Linux and Windows with no modification at all. For computational efficiency we use thread parallelization to relevant loops. Finally, it is designed in a modular way so it allows further customization.

As shown in figure 1, we use three time steps to model (fast) diffusive biotransport processes, Δt_{diff}, cell mechanics, Δt_{mech}, and (relatively slow) cell processes, Δt_{cycle}. We use these time steps to set how frequently biotransport processes, cell movement processes, and cell phenotype processes are updated.

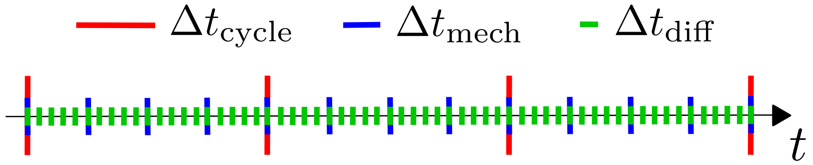

Figure 1: We use different time scales to update the different biological processes. The microenvironment is updated at the short green tick marks, corresponding to Δt_{diff}. Cell mechanics (including cell position) is less frequently updated at the medium blue tick marks (Δt_{mech}), and cell's volume and cycle/death models least frequently at the long red tick marks (Δt_{cycle}). Note that this is an schematic representation of the time steps, the default step sizes are given in section 4.

Mathematically, this time scale separation allows us to hold cell positions fixed when updating the partial differential equations (PDE) solutions, and then hold the chemical fields fixed when updating cell positions and phenotypes. These time steps can be adjusted to model different processes such as faster phenotypic processes and faster cell movement. Please find the time steps default values in section 4.

After initializing the microenvironment, the cells, and the current simulation time $t = 0$, our model tracks (internally) t_{mech} (the next time at which cell mechanics methods are run), t_{cycle} (the next time at which cell processes are run), and t_{save} (the simulation data output time), with output frequency Δt_{save}. Initially we set:

$$\begin{aligned} t_{\text{mech}} &= \Delta t_{\text{mech}} \\ t_{\text{cycle}} &= \Delta t_{\text{cycle}} \\ t_{\text{save}} &= 0 \end{aligned} \tag{1}$$

and repeat the following steps until reaching the maximum simulation time:

First we run the 3-D diffusion solver to update the biochemical microenvironment for cell-based secretions and uptake, and reaction-diffusion, for the current fixed cell positions. Then, if $t \geq t_{\text{mech}}$ we calculate the force-based cell velocities and, if the type of cell allows it, we add the contribution of motility. For each cell, we update the position using the Adams-Bashforth method and set $t_{\text{mech}} = t_{\text{mech}} + \Delta t_{\text{mech}}$.

Second, for the fixed cell positions and chemical substrate fields, if $t \geq t_{\text{cycle}}$, we run the cell processes on an individual cell basis. The first step will be to update cell parameters, such as transition and death rates, usually based on some chemical substrate (*i.e.* oxygen). Then we advance in the cell cycle or death cycle model. Based on the cycle model, we update cell's volume and geometry. Finally we set $t_{\text{cycle}} = t_{\text{cycle}} + \Delta t_{\text{cycle}}$ and move on.

If $t \geq t_{\text{save}}$ we save the simulation results and set $t_{\text{save}} = t_{\text{save}} + \Delta t_{\text{save}}$

Finally we update the current simulation time by $t = t + \Delta t_{\text{diff}}$ and start over. For a schematic view of the main loop flow, please check figure 2.

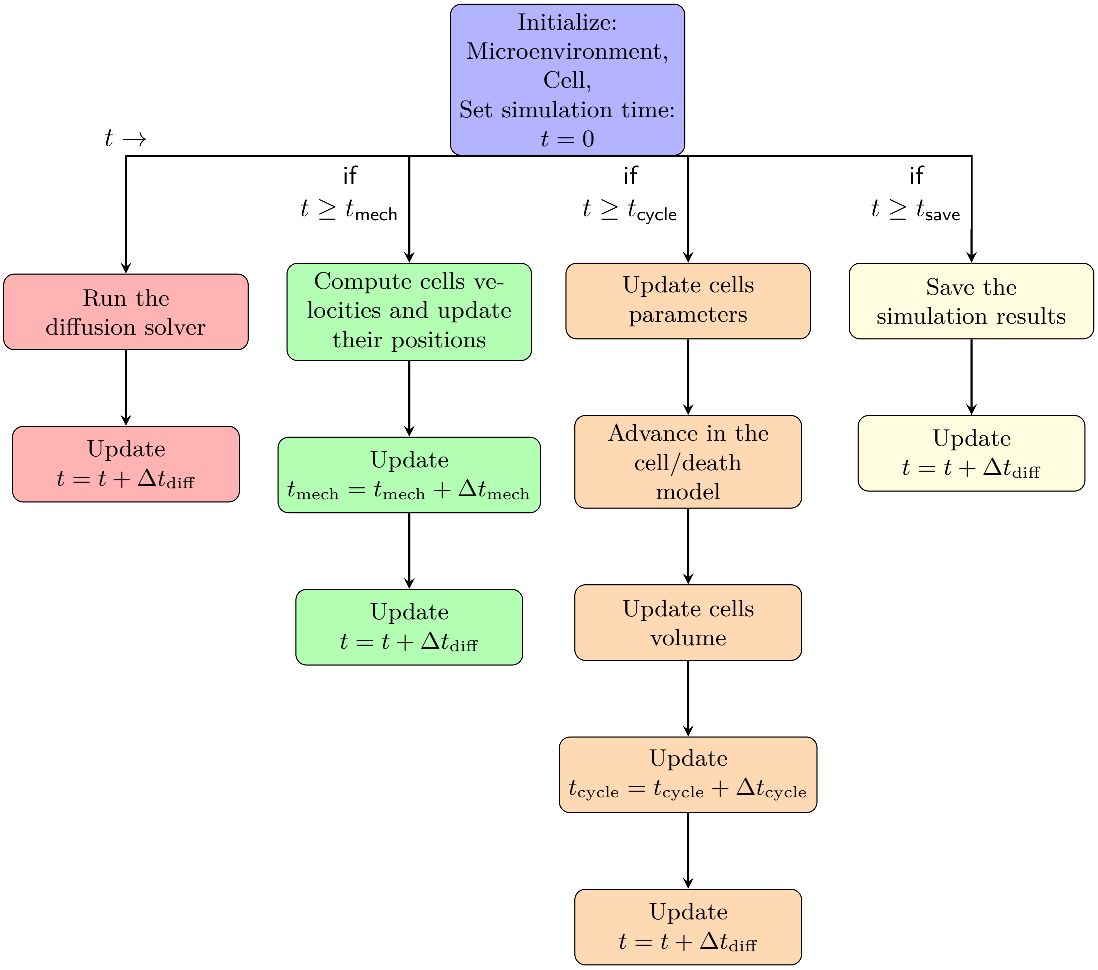

Figure 2: Main loop flow diagram. Blue box represents the start of the program. Red boxes represent the diffusion processes. Green boxes and orange boxes describe the cell mechanics and cycling processes respectively. Finally, yellow boxes represent the data saving process.

1.1 Input parameters

Parameter	Description	Units
Microenvironment Parameters		
$\Omega_x, \Omega_y, \Omega_z$	Domain size	μm
$\Delta x, \Delta y, \Delta z$	Microenvironment voxel size	μm
D	Substrates diffusion coefficients	μm^2/min
λ	Substrates decay rates	min^{-1}
BC	Set the boundary conditions	dimensionless
ρ_{BC}	BC substrates concentration	multiple
Cell Parameters		
V	Total cell volume	μm^3
V_N	Total nuclear volume	μm^3
f_{CN}	Target cytoplasmic:nuclear volume ratio	dimensionless
f_F	Target fluid fraction	dimensionless
ρ^*	Substrates saturation value	multiple
ρ_{thres}	Substrates threshold value	multiple
S	Substrates release rate	min^{-1}
U	Substrates uptake rate	min^{-1}
Cell Cycle Parameters		
T_{G1}	Duration of G_1 phase	hs
T_S	Duration of S phase	hs
T_{G2}	Duration of G_2 phase	hs
T_M	Duration of M phase	hs
div_{prob}	% of cells allowed to divide	dimensionless
Cell Mechanics Parameters		
n_x, n_y, n_z	Mechanics discretization voxel size	μm
R_A	Maximum cell adhesion distance	μm
$C_{ccr/a}$	Cell-cell "repulsive"/adhesion force constant	μm/min
$C_{cmr/a}$	Cell-membrane "repulsive"/adhesion force constant	μm/min
p	Division polarization	dimensionless

Table 1: Input parameters.

Parameter	Description	Units
Time Parameters		
T_{Tot}	Total simulation time	min
t_{save}	Saving time	min
Δt_{cycle}	Cell processes time step	min
Δt_{mech}	Cell mechanics time step	min
Δt_{diff}	Diffusion time step	min
t_v	Update voxel lists of particles time	min

Table 2: Time input parameters.

1.2 Output parameters

Every t_{save} the program saves the following information:

Parameter	Description	Units
Microenvironment Parameters		
Ω_m	Cell's microenvironment voxel	dimensionless
ρ_{O_2}	O_2 concentration in cell's voxel	mmHg
ρ_{Imm}	Immunostimulatory factor concentration in cell's voxel	dimensionless
ρ_{GF}	Growth factor concentration in cell's voxel	dimensionless
Cell Parameters		
ID	Cell's ID	dimensionless
Ω_{mech}	Cell's mechanical voxel	dimensionless
x, y, z	Cell's center position	dimensionless
r	Cell's radii	μm
op	Cell's oncoprotein expression	dimensionless
Type	Cell type, *i.e* cancer, hepatocyte, etc	dimensionless
Cycle	Cell or death cycle	dimensionless
Progenitor	Cell progenitor's ID	dimensionless

Table 3: Output parameters.

2 Biochemical microenvironment

Tissues are filled with various chemical compounds, including signaling and other factors that regulate how cells move, grow, and die, depending on the concentration and/or gradient of any and all of these compounds. In order for cells to survive and grow, they need to obtain oxygen and other nutrients released from blood vessels. In general, they need the interaction with the

stroma, the vascular network and the immune system that make up the microenvironment where autocrine and paracrine phenomena occur in a dynamic way, where changes in the phenotype can occur [1]. These chemical substances move through tissues by diffusion, and are uptaken by cells and reaction terms. These same transport processes can be used to model how chemotherapeutic drugs reach their intended targets: susceptible cancer cells, and try to understand the mechanisms of therapeutic resistance. By performing computational simulations of the movement of dozens of various chemical substances and of cellular chemical uptake and secretion rates, we can test hypotheses that can control the overall growth of cells and enable 3-D simulations of multicellular secretomics.

To simulate this motion we use a first-order, implicit (and stable) operator splitting, allowing us to create separate, optimized solvers for the diffusion-decay equation 2.

$$\frac{\partial \boldsymbol{\rho}}{\partial t} = \nabla \cdot (\mathbf{D} \circ \nabla \boldsymbol{\rho}) - \boldsymbol{\lambda} \circ \boldsymbol{\rho} \tag{2}$$

where $\boldsymbol{\rho}$ is the vector of densities, $\mathbf{D}$ are the diffusion coefficients, $\boldsymbol{\lambda}$ are the decay rates and $\circ$ denotes the Hadamard (termwise) product. Bold terms represent vectors.

Following [2], we solve the diffusion-decay terms using the Finite Volume Method (FVM) [3], further accelerated by an additional first-order splitting [4,5] into separate solutions in the $x-$, $y-$ and $z-$directions via the locally one-dimensional method (LOD) [5]. For each dimension, we solve the resulting tridiagonal linear systems with the Thomas Algorithm [6].

Next we will discuss the methods previously mentioned.

2.1 Domain discretization and notation

First of all we discretize the spatial simulation domain, Ω, into voxels (volumetric pixels) to solve the reaction-diffusion equation with the finite volume method. We use a Cartesian mesh, so the FVM reduces to a finite difference scheme. With that in mind let $\{\Omega_m\}_{m=0}^{N}$ be a set of voxels (Fig. 3) that constitutes Ω. We can write $\Omega = [x_L, x_U] \times [y_L, y_U] \times [z_L, z_U]$, where L stands for *Lower* and U stands for *Upper*. Step sizes in x, y and z directions are given by Δx, Δy and Δz

respectively.

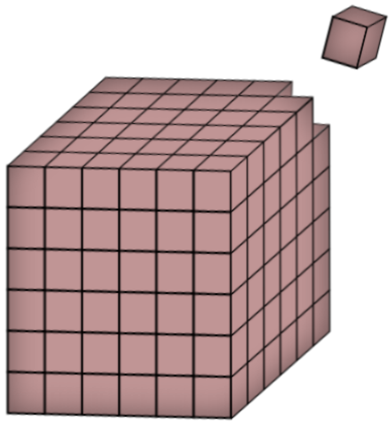

Figure 3: Domain discretization using a Cartesian mesh.

For each $(i, j, k) \in [0, N_x - 1] \times [0, N_y - 1] \times [0, N_z - 1]$ the voxels are labeled as:

$$m(i, j, k) = i + jN_x + kN_xN_y \tag{3}$$

where N_x, N_y and N_z are the nodes number in each direction. Then we can define

$$\Omega_m = \Omega_{m(i,j,k)} = \Omega_{i,j,k} = \mathbf{x}_m + \left[-\frac{\Delta x}{2}, \frac{\Delta x}{2}\right] \times \left[-\frac{\Delta y}{2}, \frac{\Delta y}{2}\right] \times \left[-\frac{\Delta z}{2}, \frac{\Delta z}{2}\right] \tag{4}$$

where $\mathbf{x}_m$ are the voxels centroids $\{\mathbf{x}_m\}_{m=0}^{N}$, given by

$$\mathbf{x}_m = \mathbf{x}_m(i, j, k) = \mathbf{x}_{i,j,k} = [x_i, y_j, z_k] \tag{5}$$

and each voxel volume will be given by

$$V_m = \Delta x \Delta y \Delta z. \tag{6}$$

Then, for a fixed time step, Δt, let $t_n = t_0 + n\Delta t$. So for any voxel Ω_m and any time $t \geq t_0$, we

can define:

$$\mathbf{u}_m^n = \int_{\Omega_m} \boldsymbol{\rho}^n(\mathbf{x}) dV \tag{7}$$

and denote the mean density $\boldsymbol{\rho}$ at time t_n in voxel Ω_m by

$$\boldsymbol{\rho}_m^n = \frac{\mathbf{u}_m^n}{V_m} = \frac{\int_{\Omega_m} \boldsymbol{\rho} dV}{V_m} \tag{8}$$

2.2 Operator splitting

Now that we have the mean density $\boldsymbol{\rho}$ at time t_n, we can solve eq. 2 by splitting the operator into simpler operators, each of which can individually be solved by tailored, optimized algorithms. Therefore, to advance the solution from $\boldsymbol{\rho}^n$ at time t_n to $\boldsymbol{\rho}^{n+1}$ at time $t_n + \Delta t$ we can use a first order splitting [4]:

$$\frac{\boldsymbol{\rho}^{n+1} - \boldsymbol{\rho}^n}{\Delta t} = \nabla \cdot \left(\mathbf{D} \circ \nabla \boldsymbol{\rho}^{n+1}\right) - \boldsymbol{\lambda} \circ \boldsymbol{\rho}^{n+1} \tag{9}$$

This uses a stable implicit time discretization.

2.3 Finite volume method

By using the Finite Volume Method (FVM) [3], volume integrals in a partial differential equation that contain a divergence term are converted to surface integrals, using the divergence theorem

$$\iint \vec{F} \cdot d\vec{S} = \iiint \nabla \vec{F} dV \tag{10}$$

we can write eq. 9 as

$$\frac{1}{\Delta t} \int_{\Omega_m} \left(\boldsymbol{\rho}^{n+1} - \boldsymbol{\rho}^n\right) dV = \int_{\partial\Omega_m} \left(\mathbf{D} \circ \nabla \boldsymbol{\rho}^{n+1}\right) \cdot \mathbf{n} dS - \int_{\Omega_m} \boldsymbol{\lambda} \circ \boldsymbol{\rho}^{n+1} dV \tag{11}$$

where $\mathbf{n_{mj}}$ is the outward normal vector from voxel m to voxel j, then

$$\frac{1}{\Delta t}\left(\boldsymbol{\rho}_m^{n+1}V_m - \mathbf{u}_m^n\right) \approx \sum_{j\in N_m} \mathbf{D}_{ij}\circ\left(\frac{\boldsymbol{\rho}_j^{n+1}-\boldsymbol{\rho}_m^{n+1}}{\Delta x_{mj}}\right)S_{mj} - \boldsymbol{\lambda}\circ\boldsymbol{\rho}_m^{n+1}V_m \tag{12}$$

Finally, we divide by V_m to obtain the implicit discretization of the FVM

$$\left(\mathbf{1}+\Delta t\boldsymbol{\lambda}+\Delta t\sum_{j\in N_m}\frac{S_{mj}}{\Delta x_{mj}V_m}\mathbf{D}_{mj}\right)\circ\boldsymbol{\rho}_m^{n+1} - \Delta t\sum_{j\in N_m}\frac{S_{mj}}{\Delta x_{mj}V_m}\mathbf{D}_{mj}\circ\boldsymbol{\rho}_m^{n+1} = \boldsymbol{\rho}_m^n \tag{13}$$

2.4 Locally one-dimensional method

We can use the locally one-dimensional (LOD) method [4,5] to split higher-dimensional PDE into a series of related one-dimensional PDEs that can be solved with fast matrix solvers. We do this by splitting the operator in eq. 2

$$\frac{\boldsymbol{\eta}-\boldsymbol{\rho}^n}{\Delta t} = \frac{\partial\left(\mathbf{D}\circ\frac{\partial\boldsymbol{\eta}}{\partial x}\right)}{\partial x} - \frac{1}{3}\boldsymbol{\lambda}\circ\boldsymbol{\eta} \tag{14}$$

$$\frac{\boldsymbol{\eta}^*-\boldsymbol{\eta}}{\Delta t} = \frac{\partial\left(\mathbf{D}\circ\frac{\partial\boldsymbol{\eta}^*}{\partial y}\right)}{\partial y} - \frac{1}{3}\boldsymbol{\lambda}\circ\boldsymbol{\eta}^* \tag{15}$$

$$\frac{\boldsymbol{\sigma}-\boldsymbol{\eta}^*}{\Delta t} = \frac{\partial\left(\mathbf{D}\circ\frac{\partial\boldsymbol{\sigma}}{\partial z}\right)}{\partial z} - \frac{1}{3}\boldsymbol{\lambda}\circ\boldsymbol{\sigma} \tag{16}$$

Applying the FVM and setting $\mathbf{D}$ constant, for each voxel i we have:

$$\left(\mathbf{1}+\frac{1}{3}\Delta t\boldsymbol{\lambda}+\frac{\Delta t\#N_i}{\Delta x^2}\mathbf{D}\right)\circ\boldsymbol{\eta}_i - \sum_{j\in N_i}\frac{\Delta t}{\Delta x^2}\mathbf{D}\circ\boldsymbol{\eta}_j = \boldsymbol{\rho}_i^n \tag{17}$$

For any fixed $0 \le j < N_y$ and $0 \le k < N_z$, and over the range $0 \le i < N_x$ (that is, for m from $m_L = m(0,j,k)$ to $m_U = m(N_x-1,j,k)$), we obtain the tridiagonal vector system:

$$\left(\mathbf{1}+\frac{1}{3}\Delta t\boldsymbol{\lambda}+\frac{\Delta t}{\Delta x^2}\mathbf{D}\right)\circ\boldsymbol{\eta}_{m_L} - \frac{\Delta t}{\Delta x^2}\mathbf{D}\circ\boldsymbol{\eta}_{m_L+1} = \boldsymbol{\rho}_{m_L}^n \tag{18}$$

$$-\frac{\Delta t}{\Delta x^2}\mathbf{D}\circ\boldsymbol{\eta}_{m-1}+\left(\mathbf{1}+\frac{1}{3}\Delta t\boldsymbol{\lambda}+2\frac{\Delta t}{\Delta x^2}\mathbf{D}\right)\circ\boldsymbol{\eta}_m-\frac{\Delta t}{\Delta x^2}\mathbf{D}\circ\boldsymbol{\eta}_{m+1}=\boldsymbol{\rho}_m^n \tag{19}$$

$$-\frac{\Delta t}{\Delta x^2}\mathbf{D}\circ\boldsymbol{\eta}_{m_U-1}+\left(\mathbf{1}+\frac{1}{3}\Delta t\boldsymbol{\lambda}+\frac{\Delta t}{\Delta x^2}\mathbf{D}\right)\circ\boldsymbol{\eta}_{m_U}=\boldsymbol{\rho}_{m_U}^n \tag{20}$$

Similar calculations in the $y-$ and $z-$ directions give additional tridiagonal linear systems to solve. This tridiagonal linear system that can be solved efficiently and directly by the Thomas algorithm [6].

2.5 Thomas algorithm

Thomas algorithm, also known as tridiagonal matrix algorithm, is a simplified form of Gaussian elimination that can be used to solve tridiagonal system of equations. A tridiagonal system for n unknowns may be written as

$$a_i x_{i-1}+b_i x_i+c_i x_{i+1}=d_i \tag{21}$$

where $a_1=0$ and $c_n=0$

$$\begin{bmatrix} b_1 & c_1 & & & 0 \\ a_2 & b_2 & c_2 & & \\ & a_3 & b_3 & \ddots & \\ & & \ddots & \ddots & c_{n-1} \\ 0 & & & a_n & b_n \end{bmatrix}\begin{bmatrix} x_1 \\ x_2 \\ x_3 \\ \vdots \\ x_n \end{bmatrix}=\begin{bmatrix} d_1 \\ d_2 \\ d_3 \\ \vdots \\ d_n \end{bmatrix} \tag{22}$$

For such systems, the solution can be obtained in $O(n)$ operations instead of $O(n^3)$ required by Gaussian elimination. A first sweep eliminates the $a_i's$, and then, an abbreviated backward substitution produces the solution.

The forward sweep consists of the computation of new coefficients as follows

$$\tilde{c}_i = \begin{cases} \frac{c_i}{b_i}; & i = 1 \\ \frac{c_i}{b_i - a_i \tilde{c}_{i-1}}; & i = 2, 3, ..., n-1 \end{cases}$$

$$\tilde{d}_i = \begin{cases} \frac{d_i}{b_i}; & i = 1 \\ \frac{d_i - a_i \tilde{d}_{i-1}}{b_i - a_i \tilde{c}_{i-1}}; & i = 2, 3, ..., n \end{cases}$$

The solution is then obtained by back substitution

$$x_n = \tilde{d}_n$$

$$x_i = \tilde{d}_i - \tilde{c}_i x_{i+1}; \ i = n-1, n-2, ..., 1$$

By applying this method to equations 18, 19 and 20 we not only have a computational efficiency of $\mathcal{O}((n-1)2 + (n-1)4)$ but, due to the fact that each x strip of voxels is independent, we can distribute and run many instances of the Thomas solver across the processor cores allowing us to easily parallelize the x diffusion problem. We solve along each of the y and z dimensions similarly.

2.6 Boundary conditions and Dirichlet nodes

In its first stage, a tumor has no vascular system of its own, so it must rely upon the host vasculature in the nearby stroma for crucial oxygen, nutrients, and growth factors (we refer to these generically as "substrates"). Substrates diffuse from the surrounding vascularized tissue, enter the tumor, and are uptaken by the cells. This motion of substrates from external sources (the host vasculature) to internal sinks (the metabolically active tumor cells) causes substrate gradients to form within the tumor. To model this we use Dirichlet boundary condition which gives the boundary value of the substrate, to be applied uniformly to each simulation boundaries. We can also turn the substrate flux off by applying a Neuman (no flux) condition for that substrate on the

simulation boundary. It is important to mention that any individual boundary can have a separate activated/deactivated state and boundary condition. Moreover the activation of each substrate can be set on an individual basis. For example we can use Dirichlet condition on oxygen but deactivate the flux of growth factor.

Another important characteristic is that we can approximate Dirichlet conditions on one or more selected voxels (*Dirichlet nodes*) by overwriting the data stored in any voxel with a prescribed vector of values. Again, we can perform that on an substrate individual basis. The code performs this step after each operator of of the LOD algorithm (i.e., after x-diffusion, after y-diffusion, and after z-diffusion; see section 2.4 above). It is very useful to model blood vessels.

2.7 Test

To validate our diffusion solver, we tested it's convergence against a 1-D problem with a known analytical solution and compared our results with those obtained by [2].

We have considered

$$\frac{\partial \rho}{\partial t} = D\frac{\partial^2 \rho}{\partial x^2} \tag{23}$$

and

$$\frac{\partial \rho}{\partial x}(x,t) = 0 \tag{24}$$

for $-L_0 < x < L_0$ and $t > 0$. With initial value

$$\rho(x,0) = 1 + \cos\left(\frac{\pi}{L_0}x\right),\ -L_0 \leq x \leq L_0 \tag{25}$$

and exact solution

$$\rho(x,t) = 1 + \exp -\beta t \cos\left(\frac{\pi}{L_0}x\right) \tag{26}$$

where $\beta = \frac{\pi^2 D}{L_0^2}$. We set the same parameters values as in [2]: $L_0 = 500\mu\text{m}$ and $D = 105\mu\text{m}^2/\text{min}$.

Our solution is plotted for several times in figure 4, which is in great agreement with [2].

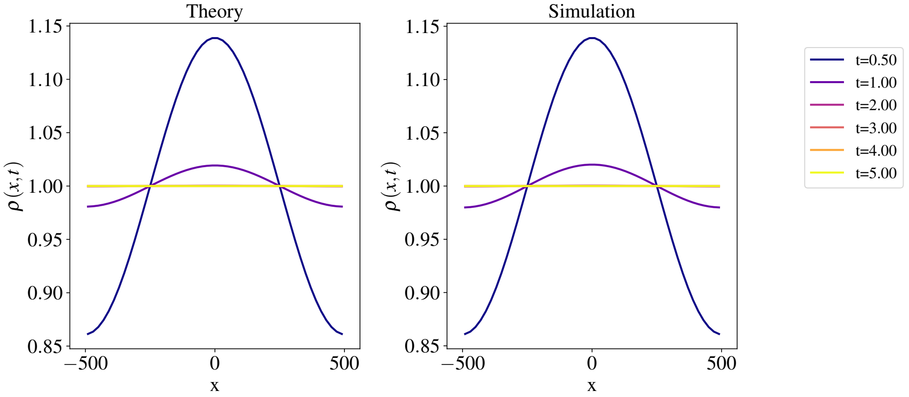

Figure 4: Solution for 1-D diffusion problem, plotted at different times.

We have also checked the convergence in space and time. We took $\rho_{\Delta t,\Delta x}$ as the numerical solution simulated with time step size Δt and spatial step size Δx. Then, for any given norm, the error for the algorithm should take the form

$$\text{Err}(\Delta t, \Delta x) = ||\rho(\mathbf{x}, t) - \rho_{\Delta t,\Delta x}((\mathbf{x}, t))|| \sim A\Delta t^m + B\Delta x^n \tag{27}$$

Therefore, to test for the convergence in Δt, we must choose Δx sufficiently small that $B\Delta x^n \ll A\Delta t^m$. In that case,

$$\text{Err} \sim A\Delta t^m \tag{28}$$

then

$$\log(\text{Err}) \sim \log(A) + m\log(\Delta t) \tag{29}$$

Thus, we calculate the order of convergence as the slope of the linear least squares fit of $\log(\text{Err})$ versus $\log(\Delta t)$.

We perform convergence testing with $\Delta x = 5\mu\text{m}$, with $\Delta t \in \{10^{-5}, 10^{-4}, 10^{-3}, 10^{-2}, 10^{-1}, 1\}$ min. The error values are plotted in figure 5 (left). We see first-order convergence in time at several

solution times (showing good accuracy on both short and long time scales). It is important to note that for any fixed Δt, the errors decrease over time; this shows that the solutions demonstrate better accuracy as solutions approach steady state.

The errors show evidence of saturating for very small Δt, where the error from the spatial discretization is likely to dominate, and so $B\Delta x^n \ll A\Delta t^m$ no longer holds for the convergence calculation. The error for $\Delta t = 1$min (an unlikely choice of time step size in most applications) is comparatively large, giving a large drop in error between $\Delta t = 1$min and $\Delta t = 0.1$min; this large drop may unduly increase the computed convergence rate.

To check the convergence in space we used $\Delta t = 10^{-5}$min to ensure $\mathrm{B}\Delta x^n \ll A\Delta t^m$ as in equation 27. Then, similarly to before

$$\log(\mathrm{Err}) \sim \log(B) + n\log(\Delta x) \tag{30}$$

and the order of convergence can be calculated as the slope of the linear least squares fit of $\log(\mathrm{Err})$ versus $\log(\Delta x)$. This errors are plotted in figure 5 (right). At all times, the solutions demonstrate approximately second-order convergence. Moreover, for any fixed Δx, the accuracy improves over time as the solution approaches steady state.

3 Cell

As it was mentioned earlier, since we are implementing an agent-based model programmed in the context of an object oriented approach, cells are agents implemented as software objects that act independently. They progress through a cell cycle (section 3.2) when preparing to divide (section 3.4), can control their entry into and exit from the cycle, and can self-terminate (apoptosis) when they detect irreparable DNA errors or other damage (section 3.7). Their behavior is governed by a signaling network that integrates genetic and proteomic information with extracellular signals received through membrane-bound receptors (section 3.8.2). In certain situations leading to another perturbations of extracellular or intracellular homeostasis or microenvironment, *i.e.* lack of oxygen, cells can respond through a variety of mechanisms, or can succumb to any kind of necrosis (i.e.

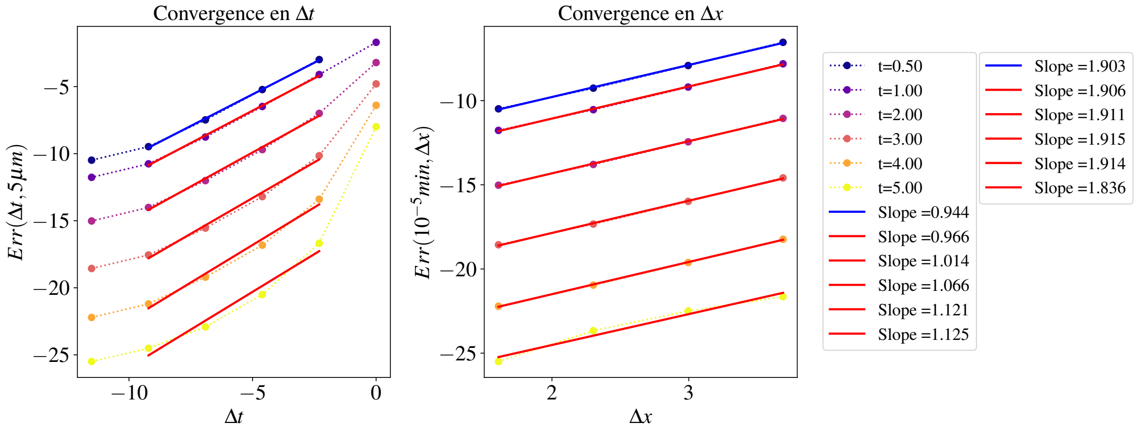

Figure 5: Left: Convergence in Δt with $\Delta x = 5\mu$m. Each curve gives the error across Δt at a different time (from top to bottom: $t = 0.5, 1, 2, 3, 4,$ and 5 min). Notice that for each fixed resolution Δt, the error improves in time as the solution approaches steady state. The linear least-squares fits are plotted for $10^{-4} \leq \Delta t \leq 10^{-1}$; the slope of each line, showed in left legend box, gives the order of convergence at that time. Right: Convergence in Δx with $\Delta t = 10^{-5}$min. Each curve gives the error across Δx at a different time. Notice that, as it happened in time convergence, for each fixed resolution Δx, the error improves in time as the solution approaches steady state. The slope of each linear least squares fit, shown in the right legend box, gives the order of convergence at that time.

necroptosis, ferroptosis, pyroptosis, etc) [7]. In pathologic conditions leading to hypoxia, *i.e.* lack of oxygen, cells can respond through a variety of mechanisms, or can succumb to necrosis (section 3.7).

In the next sections we will describe the main features of the cell.

3.1 Proliferation

Cell division is regulated by a highly regimented series of stages known as the cell cycle (Fig. 6). It has different phases in which the cell perform different actions. In the first stage we have the $G1$ (gap 1) phase, in which the cell physically grows. Proteins are synthesized, new organelles are constructed, and the cell prepares for DNA replication. The next phase is the S (synthesis) phase. Here the DNA is replicated. Next, the $G2$ (gap 2) phase is a period of protein synthesis and rapid cell growth to prepare the cell for mitosis. Final preparations are made within the cell nucleus for the division of the cell. In the final M (mitosis) phase, the two copies of the DNA are separated

and incorporated into two nuclei (mitosis), and the cytoplasm and the organelles are divided into two daughter cells (cytokinesis).

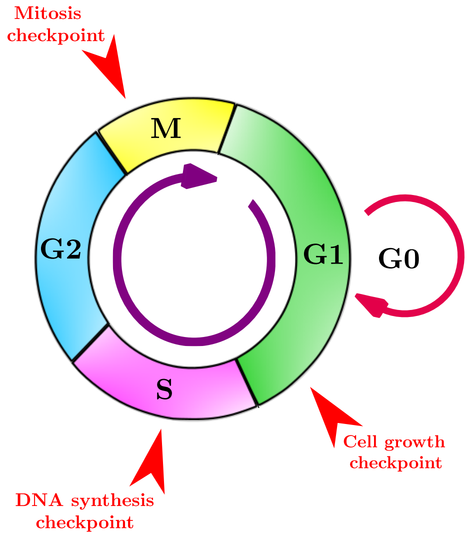

Figure 6: Cell cycle

The cell cycle contains numerous checkpoints [8] that monitor and regulate its progress. One of them is the cell growth checkpoint. It occurs toward the end of the growth phase $G1$ and checks whether the cell is big enough and has made the proper proteins for the S phase. If the cell is not ready it exits the cell cycle and enters the $G0$ quiescent state [9, 10]. Most noncancerous somatic cells stay in this quiescent to keep the tissue homeostasis. There are numerous checkpoints in the S and $G2$ phases to detect and repair DNA damage, for example the DNA synthesis checkpoint which, as its name states, checks whether the DNA has been replicated correctly. If cells overcome this checkpoint they undergo mitosis. The last checkpoint, the mitosis checkpoint, checks whether mitosis is complete. If so, cell divides and the cycle repeats. On the other hand, if cells fail to repair their DNA damage at such checkpoints induce apoptosis [11]. In the process, “executioner” proteins in the cytoplasm break down the organelles, degrade the cytoskeleton, and fragment the DNA. The cell shrinks, and the degraded cell contents are released as harmless (*i.e.*, chemically inert) vesicles known as apoptotic bodies, which are ingested (phagocytosed) by specialized immune cells as well as neighboring cells [12, 13].

The speed of the cell cycle progression is regulated by the production and balance of internal chemical signals, principally cyclins and cyclin-dependent kinases (CDKs). Surface receptors help control gene expression levels through complex signaling pathways. The gene expression pattern, in turn, determines the production and balance of proteins (including cyclins and CDKs). Hence, cell cycle progression is regulated by a complex interaction between the cells internal biomachinery and its surrounding environment [11].

As we have mentioned before, most noncancerous somatic cells stay quiescent (*i.e.* in the $G0$ phase) to keep the tissue homeostasis, but when a differentiated cell dies, a somatic stem cell (*i.e.* undifferentiated cells that reside among differentiated cells in a tissue or organ) may divide either symmetrically into two new stem cells or asymmetrically into a stem cell and a progenitor cell. The progenitor cell either further divides or terminally differentiates into the desired cell type, migrates or is pushed to the correct position, and assumes its function. This process is tightly regulated by intercellular communication via biochemical signals (growth factors) and mechanics. Each cells response to the microenvironment is governed by surface receptors that interact with an internal signaling network. In some cases such as liver regeneration, cells not only proliferate but undergo into hyperplasia process. The liver has an extraordinary capacity to regenerate from various types of injuries [14, 15]. It consists of various cells types but most of all hepatocytes, which carry out most of the metabolic and synthetic functions of the liver. In severely damaged liver with impaired hepatocyte proliferation, facultative liver stem cells proliferate and are assumed to contribute to regeneration, but after a surgical resection of a portion of the liver (partial hepatectomy, PHx), regeneration does not require such stem cells. The remnant tissue undergoes hyperplasia to recover the original liver mass. As it was discussed in [16–18] this complex process depends on the hepatectomy. It was shown that after a 70% PH no cell division was observed in more than 40% of hepatocytes and the average number of cell divisions two weeks after was estimated as 0.7 times per hepatocyte. But in the case of a 30% PH, hepatocytes did not undergo cell division at all, instead they did undergo hyperplasia.

3.2 Cycle

Based on what we have mentioned before, we model the cell cycle as a directed graph in which the nodes are the phases and the edges are the transition rates (Fig. 7)

Phase transitions times can either be deterministic i.e transition occur at a fixed time, or stochastic. The probability of transitioning from phase P_i to phase P_j is given by:

$$\text{Prob}\left(P_i(t) + P_j(t+\Delta t)\right) = 1 - \exp\left(-r_{ij}\Delta t\right) \approx r_{ij}\Delta t; \tag{31}$$

where r_{ij} are the transition rates given by $1/t_i$ in which t_i represents the time that takes to the cell to go through the phase i.

It is important to point out that we can adjust the graph to model different types of cell cycles depending on the information we have. For example, when studying liver regeneration, cell proliferation is characterized by the expression of a proliferation protein called Ki-67 [17]. This protein expresses when the cell is proliferative, *i.e.* in the S, $G2$, and M phases [19], and to a lesser extent in the $G1$ phase [20]. Ki-67 is seen in post-mitotic daughter cells [21], but it is not produced in these cells [19]. Instead, any remaining Ki-67 protein in post-mitotic cells is degraded quickly, with a half-life of $60 - 90$ minutes [19].

We can relate both cycles by setting the phase "Ki-67+ pre-mitotic" ($K+_{\text{pre}}$) to be the combined duration of S, $G2$, and M phases, which are relatively fixed compared to the duration of $G0/G1$ [22, 23]. As we have mentioned before, after mitosis, Ki-67 protein is degraded quickly, so we set the second phase "Ki-67+ post-mitotic" ($K+_{\text{post}}$) that takes place at the $G1/G0$ phase, to be on the order of two Ki-67 half-lives (we use an intermediate estimate of a 75 minutes half-life). Then, the remaining $G1/G0$ phase will be the "Ki-67-" ($K-$) phase. The calibration of the cell cycles times will depend on the types of cell under study. Please refer to section 4 for more information.

One important aspect is that the transition rates can change based upon microenvironmental conditions (e.g. it may increase with oxygenation). For example, consider the Ki-67 cell cycle (fig. 8). Phases ($K+_{\text{pre}}$) and ($K+_{\text{post}}$) have stochastic durations, which means that the cell will spent a mean of $t_{K+_{\text{pre}}}$ and $t_{K+_{\text{post}}}$ in each one respectively, but phase $K-$ will depend on the oxygenation

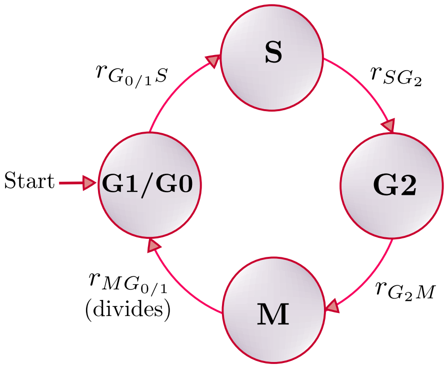

Figure 7: Schematic representation of the cell cycle

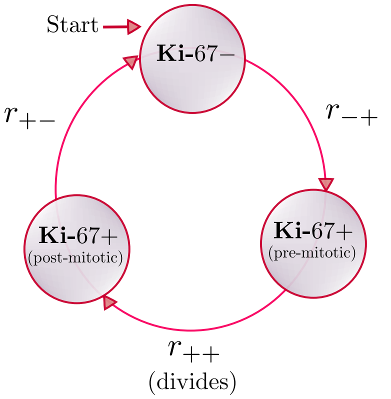

Figure 8: Cell cycle based on the expression of the protein $Ki - 67$

of the tissue. Then we can model the transition from Ki-67− to Ki-67+ pre-mitotic as:

$$r_{-+} = \frac{1}{t_{K-}} \max \left\{ \left(\frac{O_2 - O_{2,prol}}{O_2^* - O_{2,prol}} \right), 0 \right\} \tag{32}$$

where O_2 is the current oxygen value in the cell's voxel $O_{2,prol}$ is the proliferation threshold, *i.e.* the oxygen value below which the proliferation ceases and O_2^* is the proliferation saturation value, above which the proliferation rate is maximized. No further oxygenation benefits the cell. In other words, cells will spend a mean time of t_{K-} if $O_2 \geq O_2^*$. Numerical values of those parameters can be found at section 4.

3.3 Volume and geometry

To model cell volume variation, each cell tracks V (total volume), V_F (total fluid volume), V_S (total solid volume), V_{NS} (nuclear solid volume), V_{CS} (cytoplasmic solid volume), V_N (total nuclear volume), and V_C (total cytoplasmic volume). Key parameters include nuclear solid, cytoplasmic solid, and fluid rate change parameters (r_N, r_C, and r_F), the cells "target" fluid fraction f_F, target solid volume V_{NS}^*, and target cytoplasmic to nuclear volume ratio f_{CN}. For each cell, these volumes are modeled with a system of ordinary differential equations (ODEs):

$$\frac{dV_F}{dt} = r_F\left(V_F^* - V_F(t)\right), \tag{33}$$

$$\frac{dV_{NS}}{dt} = r_N\left(V_{NS}^* - V_{NS}(t)\right), \tag{34}$$

$$\frac{dV_{CS}}{dt} = r_C\left(V_{CS}^* - V_{CS}(t)\right), \tag{35}$$

where r_F , r_N, and r_C are variation rate constants, and V_F^* , V_{NS}^* and V_{CS}^* are the target volumes that cells must reach in each phase. The remaining volumes can be calculated by

$$V_{CS} = \left(1 - \frac{V_F}{V}\right) V_C; \tag{36}$$

$$V_{NS} = \left(1 - \frac{V_F}{V}\right) V_N; \tag{37}$$

$$V_S = V_{CS} + V_{NS}; \tag{38}$$

using

$$V_{CS}^*(t) = f_{CN} V_{NS}^*(t), \tag{39}$$

$$V_F^*(t) = f_F V(t), \tag{40}$$

where, $f_{CN} = 1/f_{NC}$ is the target cytoplasmic to nuclear volume ratio and f_F is the cells target water fraction.

Target volumes and transition rates will depend on the cell type and its corresponding cell cycle, but we can estimate those parameters to any type of cell as follows:

First we estimate the water fraction f_F. For that we consider that a typical cell water mass

fraction f_m is 70% [24,25]. Now let ρ be the mass density of water, ρ_S be the relative mass density of the solid cell fraction and V_S be the total solid cell volume, so that $V = F_F + V_S$. Since typically $\rho_S \approx 1.3\rho$ [26,27], we can solve for V_S:

$$f_m = \frac{m_F}{m_F + m_S} = \frac{\rho V_F}{\rho V_F + \rho_S V_S} = \frac{\rho V_F}{\rho V_F + 1.3\rho V_S} \tag{41}$$

where m_F and m_S are the cell's total fluid and solid masses respectively. Then we get

$$V_S = \frac{1 - f_m}{1.3 f_m} V_F \tag{42}$$

Next,

$$f_F = \frac{V_F}{V_F + V_S} = \frac{1}{1 + \frac{V_S}{V_F}} = \frac{1}{1 + \frac{1-f_m}{1.3 f_m}} \tag{43}$$

Taking $f_m = 0.70$ then $f_F \approx 0.75$. With that in mind and knowing the nuclear volume of the type of cells we are working with, we can compute $V_{NS}^* = (1 - f_F)V_N$.

By knowing the cell cycle times, we can compute the rates parameters as follows. We set r_N so that 95% of the nuclear solid volume doubles in the S-phase time, $T_{\text{S-Phase}}$. Then the analytical solution for eq. 34, with $V_{NS}^* = 2$ and $V_{NS}(0) = 1$, gives:

$$1 + 0.95 = 2 - (2 - 1)\exp\left(-r_N t_{\text{S-Phase}}\right) \longrightarrow r_N \approx -\frac{\ln(0.05)}{t_{\text{S-Phase}}} \tag{44}$$

To estimate the cytoplasmic solid biomass creation rate r_C we set it sufficiently large to ensure that the biomass is 95% doubled within a short cell cycle. Moreover, because cytoplasmic biomass creation lags nuclear biomass creation in this model (which happens primarily in the S phase), we set r_C fast enough to ensure that 95% of the biomass is created within the last half of the S-phase until the first part of $G1$ phase. In the Ki-67 cell cycle, this process would happen during the positive phases $K+_{\text{pre}}$ and $K+_{\text{post}}$. If t_C denotes this time scale, the analytical solution to equation 35 (with target volume $V_{CS}^* = 2$ and initial volume $V_{CS}(0) = 1$) gives

$$1 + 0.95 = 2 - (2 - 1)\exp(-r_C t_C) \longrightarrow r_C \approx -\frac{\ln(0.05)}{t_C} \tag{45}$$

Finally, the water transport parameter, r_F, must be set sufficiently large to match the biomass creation, with a time scale t_F. So we estimate t_F to be one order of magnitude faster than t_C. Assuming that the 95% of the targeted water flux occurs within the time scale t_F we have

$$1 + 0.95 = 2 - (2 - 1)\exp(-r_F t_F) \longrightarrow r_F \approx -\frac{\ln(0.05)}{t_F} \tag{46}$$

The numerical values of these volume variation rates depends on the cell cycle times. They can be found in section 4 for each specific type of cell.

3.4 Division

When a cell divides, we halve all it's sub-volumes and copy all state and parameter values into it's daughter. To decide in which position the daughter will born we let $0 \leq p \leq 1$ be the degree of polarization (fully polarized if $p = 1$ and completely random if $p = 0$), and $\boldsymbol{\theta}$ be the cell's unit orientation vector (directed from cell base to cell apex). Then we define

$$\mathbf{d} = \frac{\mathbf{r} - (\mathbf{r}\cdot\boldsymbol{\theta})\boldsymbol{\theta} + ((1-p)(\mathbf{r}\cdot\boldsymbol{\theta}))\boldsymbol{\theta}}{||\mathbf{r} - (\mathbf{r}\cdot\boldsymbol{\theta})\boldsymbol{\theta} + ((1-p)(\mathbf{r}\cdot\boldsymbol{\theta}))\boldsymbol{\theta}||} \tag{47}$$

If V is the volume of the parent cell at the time of division, with equivalent radius R, the daughter cells will have equivalent radius $\frac{1}{\sqrt[3]{2}}R$. Now, if $\mathbf{x}_{\text{parent}}$ is the center of the parent cell, we can place the two daughter cells at

$$\mathbf{x}_{\text{daughters}} = \mathbf{x}_{\text{parent}} \pm \left(R - \frac{1}{\sqrt[3]{2}}R\right)\mathbf{d}. \tag{48}$$

By combining the last three subsections, we can see in Fig. 9 how cell volume and sub-volumes changes as it goes through the cell cycle and divides.

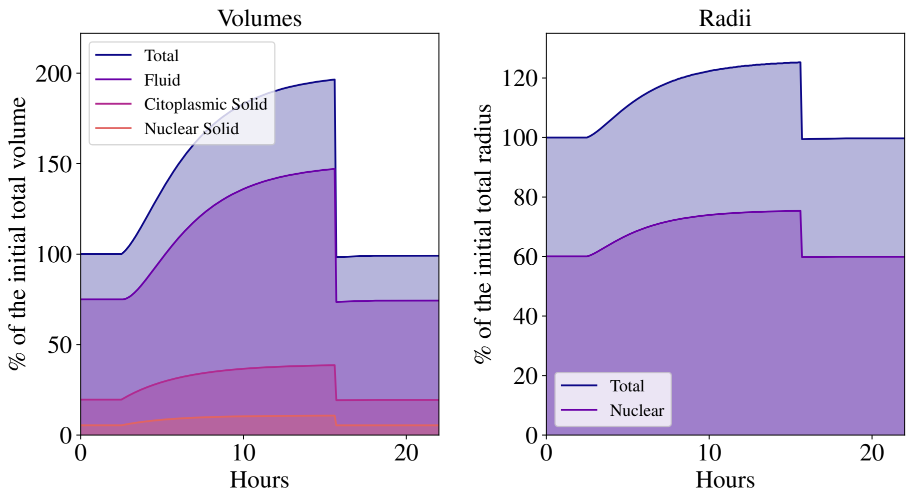

Figure 9: Cell's sub volumes and radii variation

3.5 Mechanics

Adhesion between cells happens when receptors on a cells surface bond with the adhesive ligands present on nearby cells. The closer the cells are, the more surface area and therefore, receptorligand pairs, get into direct contact. Hence, the adhesive force between the cells increases as the cells are drawn more closely together [28]. On the other hand cells resist compression by other cells due to the structure of their cytoskeletons [29, 30]. In that sense, the repulsive force between cells is zero when cells are just touching, and increases rapidly when cells are pressed together.

To reproduce that behavior, we have consider different forces models, such as the linear spring model [31, 32] and the Johnson-Kendall-Roberts (JKR) model [33]. In the first case the force is assumed to be directly proportional to the distance between cell midpoints, vanishing at the rest length. While this behavior may be reasonable for small distances, it results in unphysically strong long-range interactions if extended to distances larger than the rest length. At the same time, a linear force does not result in very large repulsive forces when cells are very close, *i.e.* cells are highly compressible. This can lead to the problem of collapsing volumes, where a cell population collapses on itself under strong compression [34]. Additionally, the linear force will feature a large discontinuity at the maximum interaction range which can lead to further numerical difficulties. The

JKR force model, has been confirmed experimentally to be valid for biological cells under certain conditions, however, it turned out to be computationally very expensive since it requires the solution of an implicit equation in order to recover the force for a given center-center distance [35]. We have then decided to implement the piecewise polynomial force model. This model is constructed as the sum of a positive adhesive and a negative repulsive polynomial force contributions. To compute these forces it uses adhesive and repulsive interaction potentials functions that depend upon each cell's size, maximum adhesion distance, adhesion and repulsion parameters and distance to other cells. It solves the issues of the linear spring model and strikes an attractive balance between numerical efficiency and the capability to reproduce cell mechanics and biophysical measurements. This force model has been tested and validated in numerous works [36–39]. Please refer to ref. [40] for further description on these interaction forces models.

In order to compute the piecewise polynomial forces we use adhesive (Φ), and repulsive (Ψ), interaction potentials functions defined as follows

$$\nabla \Phi_{\text{c-c}} = \begin{cases} \left(1 - \frac{|\mathbf{x_j} - \mathbf{x_i}|}{R_{i,A} + R_{j,A}}\right)^{n_{\text{cca}}+1} \frac{\mathbf{x_j} - \mathbf{x_i}}{|\mathbf{x_j} - \mathbf{x_i}|} & \text{if } |\mathbf{x_j} - \mathbf{x_i}| \leq R_{i,A} + R_{j,A} \\ \mathbf{0} & \text{otherwise} \end{cases} \tag{49}$$

$$\nabla \Psi_{\text{c-c}} = \begin{cases} -\left(1 - \frac{|\mathbf{x_j} - \mathbf{x_i}|}{R_i + R_j}\right)^{n_{\text{ccr}}+1} \frac{\mathbf{x_j} - \mathbf{x_i}}{|\mathbf{x_j} - \mathbf{x_i}|} & \text{if } |\mathbf{x_j} - \mathbf{x_i}| \leq R_i + Rj \\ \mathbf{0} & \text{otherwise} \end{cases} \tag{50}$$

where n_{cca} and n_{ccr} are integer powers chosen for the smoothness of the force's behavior at the edge of interaction. R_i, R_j and $R_{i,A}$, $R_{j,A}$ are cells i, j radii and maximum adhesive interaction distances respectively, and $\mathbf{x_i}$, $\mathbf{x_j}$ are the cells centers positions.

Our model also supports cell-basement membrane adhesive and repulsive interaction, so for cell-basement membrane forces we use

$$\nabla \Phi_{\text{c-bm}} = \begin{cases} \left(1 - \frac{|\mathbf{d}|}{R_{i,A}}\right)^{n_{\text{c-bma}}+1} \frac{\mathbf{d}}{|\mathbf{d}|} & \text{if } |\mathbf{d}| \leq R_{i,A} \\ \mathbf{0} & \text{otherwise} \end{cases} \tag{51}$$

$$\nabla \Psi_{\text{c-bm}} = \begin{cases} -\left(1 - \frac{|\mathbf{d}|}{R_i}\right)^{n_{\text{c-bmr}}+1} \frac{\mathbf{d}}{|\mathbf{d}|} & \text{if } |\mathbf{d}| \leq R_i \\ \mathbf{0} & \text{otherwise} \end{cases} \tag{52}$$

where $\mathbf{d}$ is the distance from i cell center to the basement membrane.

Now that we have the interaction potentials we can compute the forces as follows, for cell-cell interaction:

$$\mathbf{F}^{ij}_{\text{cca}} = -C_{\text{cca}} \nabla \Phi_{\text{c-c}} \tag{53}$$

$$\mathbf{F}^{ij}_{\text{ccr}} = -C_{\text{ccr}} \nabla \Psi_{\text{c-c}} \tag{54}$$

where C_{cca} and C_{ccr} are the cell-cell adhesion and repulsion parameters respectively that takes into account the deformability of the cells, so to compute their value we need to know how confluent the tissue is. In cell culture biology, cell confluence is defined as the percentage of a culture dish or a flask occupied by any type of adherent mammalian cells [41]. For example, 50 percent confluence means roughly half of the surface is covered, while 100 percent confluence means the surface is completely covered by the cells, and no more room is left for the cells to grow as a monolayer. Although we must point out that tumor cells have altered the phenomenon of contact inhibition and show multilayer growth [42]. So we can estimate the mean cell-cell equilibrium distance, s, for a tissue with confluence f, where $0 \leq f \leq 1$. Due to the mechanics the cells obey, they will tend to form a hexagonal closed-packed structure as shown in figures 10 and 11.

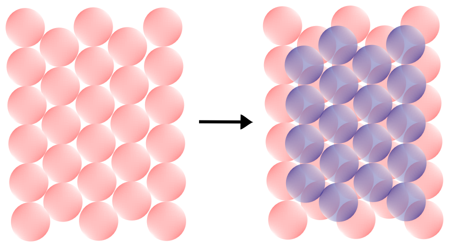

Figure 10: Hexagonal closed-packed tissue.

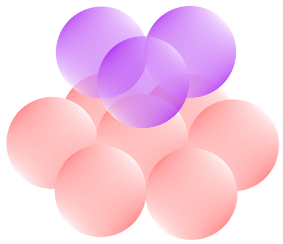

Figure 11: Closer look at the hexagonal closed-packed tissue.

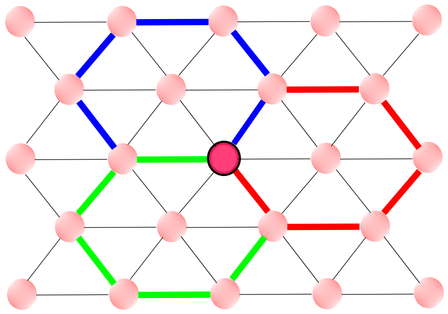

Figure 12: Shared hexagons of the darker cell.

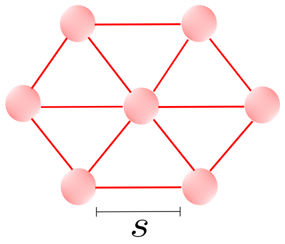

Figure 13: Equilibrium distance, s, between cells

Keeping in mind that each cell shares three hexagons as shown in figures 10 and 12, we can compute the density ρ as

$$\rho = \frac{3f}{A_{Hex}} \tag{55}$$

where A_{Hex} is the hexagon area given by

$$A_{Hex} = \frac{3\sqrt{3}s^2}{2} \tag{56}$$

where s is the equilibrium distance as shown in figure 13, then

$$\rho = \frac{3f}{\frac{3\sqrt{3}s^2}{2}} \tag{57}$$

so the the equilibrium distance s will be given by

$$s = \sqrt{\frac{2f}{\sqrt{3}\rho}} \tag{58}$$

Next, for two cells, i, j, at the equilibrium distance s, adhesive and repulsive forces are equal, $F^{ij}_{\text{cca}} = F^{ij}_{\text{ccr}} = 0$, so:

$$
\begin{aligned}
F^{ij}_{\text{cca}} &= F^{ij}_{\text{ccr}} \\
C_{\text{cca}} \nabla \Phi_{\text{c-c}} &= C_{\text{ccr}} \nabla \Psi_{\text{c-c}} \\
C_{\text{cca}} \left(1 - \frac{|\mathbf{s}|}{R_{i,A} + R_{j,A}}\right)^{n_{\text{c-bma}}+1} \frac{\mathbf{s}}{|\mathbf{s}|} &= C_{\text{ccr}} \left(1 - \frac{|\mathbf{s}|}{R_i + R_j}\right)^{n_{\text{c-bmr}}+1} \frac{\mathbf{s}}{|\mathbf{s}|} \\
C_{\text{cca}} &= C_{\text{ccr}} \frac{\left(1 - \frac{|\mathbf{s}|}{R_i + R_j}\right)^2}{\left(1 - \frac{|\mathbf{s}|}{R_{i,A} + R_{j,A}}\right)^2}
\end{aligned}
\tag{59}
$$

Consequently, the piecewise quadratic function has one free parameter, C_{ccr}.

For the cell-basement membrane forces we proceed as with the cell-cell forces, hence

$$
\mathbf{F}^i_{\text{cmba}} = -C_{\text{cmba}} \nabla \Phi_{\text{c-mb}} \tag{60}
$$

$$
\mathbf{F}^i_{\text{cmbr}} = -C_{\text{cmbr}} \nabla \Psi_{\text{c-mb}} \tag{61}
$$

Now we can update cells positions by using Newton's second law

$$
m_i \frac{d\mathbf{v_i}}{dt} = \sum \mathbf{F} \tag{62}
$$

We assume that three types of forces act on each cell. First we have a drag force, which represents dissipative, drag-like forces such as fluid drag and cell-extra cellular matrix adhesion. Cells friction with the viscous environment is directed against the cells direction of motion so

$$
\mathbf{F}^i_{\text{drag}} = -\nu \mathbf{v_i} \tag{63}
$$

where ν is the drag coefficient.

Secondly, as we have mentioned before, neighboring cells exert mechanical forces on the cell. In the simplest case these involve repulsive forces due to limited cell compressibility, but they usually also include cell-cell adhesion. Interactions are assumed to be pairwise and symmetric, *i.e.*

$$\mathbf{F}^{ij}_{\text{cells}} = \sum_{j \neq i} \mathbf{F}_{ij} \tag{64}$$

where the sum runs over all neighbors, excluding the cell itself. What is consider a cell neighbor will be discussed later on in section 3.5.1.

Finally, the third force acting on the cells is the cell-basement membrane, such that

$$\mathbf{F}^{i}_{\text{c-bm}} = \sum_{i} \mathbf{F}_{i} \tag{65}$$

So the resulting governing equation for cell mechanics reads

$$m_i \frac{d\mathbf{v_i}}{dt} = \sum \mathbf{F}^{ij}_{\text{cca}} + \mathbf{F}^{ij}_{\text{ccr}} + \mathbf{F}^{i}_{\text{cmba}} + \mathbf{F}^{i}_{\text{cmbr}} + \mathbf{F}^{i}_{\text{drag}} \tag{66}$$

This is a system of second-order ordinary differential equations governing the cell center positions, with one equation for each degree of freedom.

It is important to note that the world of microscopic organisms involves fluids that are highly viscous in nature and dominate any inertial forces that may be present [43]. Given these conditions, the physics that governs the movements of these organisms at a microscopic level can be described by a low Reynolds Number [44,45]. The Reynolds Number is a dimensionless quantity that is based on the Navier-Stokes equation, which describes the motion of an incompressible Newtonian fluid. It represents the ratio of inertia to viscosity of a fluid [46] and it allows for a qualitative description of the flow regime from the Navier-Stokes equation [45]. In this context, biological cells are highly dissipative objects, for which viscous forces greatly exceed inertial forces. Thus, the motion of cell deformations due to the force acting on the cell membrane is usually assumed to be overdamped, with inertial terms being negligible when compared to dissipative terms. This is commonly known as the inertialess assumption, $m_i \frac{d\mathbf{v_i}}{dt} \approx \mathbf{0}$, and assumes that forces equilibrate at relatively fast time scales in contrast to the ones of cell cycling, death cycling and volume variation. Then, equation 66 reduces to

$$\mathbf{v_i} = \frac{1}{\nu_i}\left(\sum \mathbf{F}_{\text{cca}}^{ij} + \mathbf{F}_{\text{ccr}}^{ij} + \mathbf{F}_{\text{cmba}}^{i} + \mathbf{F}_{\text{cmbr}}^{i}\right) \tag{67}$$

Now we can use numerical methods to solve this equation.

For an initial value problem stated as

$$\frac{d\mathbf{x}}{dt} = f(t, \mathbf{x}) \tag{68}$$

with

$$\mathbf{x}(t_0) = \mathbf{x_0} \tag{69}$$

a numerical scheme provides an approximation for function values $\mathbf{x_n} \approx \mathbf{x}(t_n)$ at discrete time points $t_n, n = 1, ..., N$. The simplest numerical scheme is the forward Euler method. It calculates the next function value by taking a step in the direction of the current gradient [47]. This method is a first-order scheme, meaning that as long as the time step, Δt, is sufficiently small, the local error in one single time step is proportional to Δt^2 and the global approximation error is proportional to Δt. That means that if one halves the step size for a first-order scheme makes the solution twice as accurate.

Higher order schemes such as the midpoint rule [48], improve the convergence rate at the cost of additional function evaluations. This is a second order method which means that halving the step size divides the error by four. Hence, less steps are needed to achieve a given accuracy compared to the forward Euler method, although each single step will be more costly.

Both methods are one-step methods, meaning that they calculate the function value at the next time point based on only the current function value. Multi-step methods additionally take past function values into account. One of the simplest two-step methods is the Adams-Bashforth method [47],

$$\mathbf{x_i}(t + \Delta t) = \mathbf{x_i}(t) + \frac{1}{2}\Delta t\left(3\mathbf{v_i}(t) - \mathbf{v_i}(t - \Delta t)\right) \tag{70}$$

In addition to the order of the scheme, the schemes stability is an important characteristic, a numerically unstable solution will oscillate and grow without bounds, even though the true solution does not. In this case the step size needs to be reduced to recover a stable, bounded solution. The stability region depends on both the numerical scheme and on the ODE problem to be solved and this imposes an upper bound on Δt for which the scheme can be used. The forward Euler method has very poor stability properties and may hence require very small time steps. For all the previously mentioned, we use the Adams-Bashforth method to compute the cell position.

3.5.1 Numerical implementation

When working with cell interactions, the efficiency of the program is mainly determined by the efficiency of the computation of the forces which act upon those cells [49]. For example, lets assume a simulation with $N = 1000$ cells. In each time step all possible pairs of cells have to be considered with respect to their interaction force, hence, $N(N-1)/2 \approx 500000$ force computations are needed. In our model we work with short-range interactions, meaning that most of those force evaluations are unnecessary because the corresponding cells are located far away from each other. Moreover, for approximately equal-sized cells each single particle can be in contact with not more than about 12 other cells (Fig. 11), hence, only about $6N = 6000$ force computations are necessary. This number is notably smaller than the 500000 computed before and allows us save a lot of computer time. Therefore, we need a method to reduce the number of pair interactions which are considered at each time step.

As we have mentioned before, the forces we are considering are of short range so the force computation can be restricted to pairs of cells which are nearest neighbors. But to decide which cells are nearest neighbors is not trivial at all since every pair of close cells have to be considered. The intuitive solution would be to simply check whether all pairs of cells are neighbors or not, but this will lead to about 500000 computations again. To avoid it we have implemented the so called Link cell algorithm. Here the simulation domain of size $N_x \times N_y \times N_z$ is discretized into $n_x \times n_y \times n_z$ identical rectangular voxels. It is very important to notice that the discretization of the domain for the mechanics computations is not the same discretization that we have used for the diffusion in the

biochemical microenvironment. The voxel size is bigger in the mechanical discretization that the one in the diffusion discretization (section 4). But the most significative difference is the neighbor lists. For the microenvironment discretization we have used a Von Neumann neighborhood (Fig. 14) in which each voxel has 6 neighbors connected by their faces. While in the mechanics discretization we have used the Moore neighborhood (Fig. 15), in which each voxel has 26 neighbors, that is, every voxel in contact with the mean voxel.

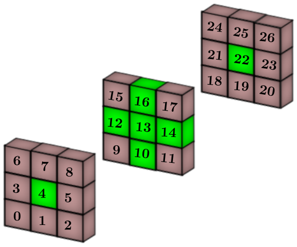

Figure 14: Von Neumann neighborhood (green voxels).

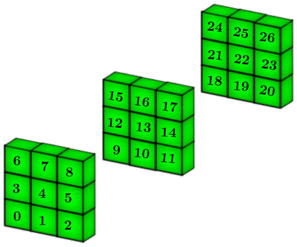

Figure 15: Moore neighborhood (green voxels).

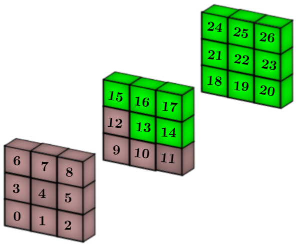

Figure 16: Moore neighborhood restricted by the commutativity relation.

Each cell is assigned to the voxel which contains its center so each voxel stores a list of cells that reside within it. These lists are used to reduce the number of force evaluations. It is worth mentioning that since these lists are used to identify collision partners, the voxel size must exceed the diameter of the largest cell. Then, for example, any cell belonging to voxel 13 (Fig. 15) can then interact only with cells from the same voxel or from the neighbor voxels $0-26$. Using commutativity of the neighborhood relation (if i is a neighbor of j then j is a neighbor of i too), it is sufficient to restrict the search to the voxels $13-26$ (Fig. 16).

To efficiently determine the possible collision partners of cells, at the beginning of the simulation each voxel is assigned a list of neighboring voxels which stays invariant during the simulation. Those list will depend on how we take the boundary conditions. In our model we can chose between periodic boundary conditions, free boundary conditions (walls) or both. Finally the cell interaction forces can be computed using the list of cells stored in each voxel and the ones stored in the adjacent voxels. In our model the interaction forces with all other cells located in the same voxel are first

computed, then we proceed with the interaction with the cells located in the neighboring voxels.

Because neighborhood relations between particles change only slowly, *i.e.*, two particles which are close to each other at a given time will stay close, at least in the following few time steps, we update the voxel lists of particles every t_v (numerical value in section 4). But even if the lists were constructed in each simulation time step this operation is not too time consuming since it scales as $\mathcal{O}(N)$.

3.6 Secretion and uptake

This is one of the most important parts of the cell's data structure because it links it to the microenvironment. We add the cell supply/uptake term to equation 2 such that for each cell k, Ω_{mk} be the voxel containing the cell center *i.e.* $\mathbf{x}_k \in \Omega_{mk}$

$$\frac{\partial \boldsymbol{\rho}}{\partial t} = \nabla \cdot (\mathbf{D} \circ \nabla \boldsymbol{\rho}) - \boldsymbol{\lambda} \circ \boldsymbol{\rho} + \sum_{cells} W_k \delta(\mathbf{x} - \mathbf{x}_k) \left[\mathbf{S}_k \circ \left(\boldsymbol{\rho}_k^* - \boldsymbol{\rho}^{n+1}\right) - \mathbf{U}_k \circ \boldsymbol{\rho}^{n+1}\right] \tag{71}$$

where W_k is the cell's volume, $\mathbf{x}$, and $\mathbf{x}_k$ are voxel's and cell's centers, $\mathbf{U}_k$ are the cell's uptake rates, $\mathbf{S}_k$ are the cell's secretion rates and $\boldsymbol{\rho}^*$ are the saturation densities. $\delta(\mathbf{x} - \mathbf{x}_k)$ is the Dirac delta function, which we approximate by

$$\delta(\mathbf{x} - \mathbf{x}_k) \approx \begin{cases} \frac{1}{V_{ik}} & \text{if } \mathbf{x} \in \Omega_{ik} \\ 0 & \text{elsewhere} \end{cases}$$

We solve for ρ^{n+1} with the implicit time discretization as we did in section 2 with the microenvironment diffusion solver

$$\frac{\boldsymbol{\rho}^{n+1} - \boldsymbol{\sigma}}{\Delta t} = \sum_{cells} W_k \delta(\mathbf{x} - \mathbf{x}_k) \left[\mathbf{S}_k \circ \left(\boldsymbol{\rho}_k^* - \boldsymbol{\rho}^{n+1}\right) - \mathbf{U}_k \circ \boldsymbol{\rho}^{n+1}\right] \tag{72}$$

Finally, we iterate over all cells k with

$$\boldsymbol{\rho}_{ik}^{n+1} = \left(\boldsymbol{\sigma}_{mk} + \Delta t \frac{W_k}{V_{mk}} \mathbf{S}_k \circ \boldsymbol{\rho}_k^*\right) \oslash \left(\mathbf{1} + \Delta t \frac{W_k}{V_{mk}} \left(\mathbf{S}_k + \mathbf{U}_k\right)\right) \tag{73}$$

where $/\!\!/$ represents termwise division. These operations can be safely parallelized across the processor cores.

3.7 Death

At a cell scale death is not an instantaneous event but a process, which means that the cell is dying, not death. In fact it is using energy to degrade. Cell death has traditionally been described in morphological terms (apoptosis, autophagy, necrosis) but since 2005 the Nomenclature Committee on Cell Death (NCDD) presented a series of details in the terminology of regulated cell death (Intrinsic apoptosis, Extrinsic apoptosis, Necroptosis, Ferroptosis, Pyroptosis, Pantharnos, Entotic cell death, NETotic cell death, etc) outlining the phenomena that initiate it and differentiating the different molecular mechanisms involved but having as a starting point the initial morphological classification [7]. We model the death process in a similar manner as we did with the cell cycle, so we have death models. At any time, each agent has one or more death rates which can be continually updated. For any death rate r_i and any time interval $[t, t+\Delta t]$, the cell has a probability of entering the corresponding death state given by

$$1 - \exp\left(r_i \Delta t\right) \approx r_i \Delta t \tag{74}$$

We model two types of deaths: *Apoptosis* and *Necrosis*.

3.7.1 Apoptosis

As we have mentioned before, if cells fail to repair their DNA damage they induce apoptosis. Apoptosis is a form of programmed cell death that occurs in multicellular organisms [50]. Biochemical events lead to characteristic cell changes (morphology) and death. These changes include blebbing, cell shrinkage, nuclear fragmentation, chromatin condensation, DNA fragmentation, and mRNA decay. It is a highly regulated and controlled process that confers advantages during an organism's life cycle. Apoptosis can be initiated through one of two pathways. In the intrinsic pathway the cell kills itself because it senses cell stress, while in the extrinsic pathway the cell kills

itself because of signals from other cells. Weak external signals may also activate the intrinsic pathway of apoptosis [51]. Both pathways induce cell death by activating caspases, which are proteases, or enzymes that degrade proteins. The two pathways both activate initiator caspases, which then activate executioner caspases, which then kill the cell by degrading proteins indiscriminately.

Upon entering the apoptosis death model, we set the target nuclear solid volume $V_{NS}^* = 0$ to simulate the degradation of the nucleus, $f_{CN} = 0$ to simulate the shrinking and blebbing of the cytoplasm, and $f_F = 0$ to simulate the active elimination of water from the cell. The rate parameters r_N, r_C, and r_F are reset with apoptosis-specific rates to reflect the timescales of nuclear degradation, water loss, and cytoplasmic blebbing. Water loss occurs relatively quickly as cytoplasmic blebbing in contrast to the nucleus degradation. Please check the parameters values in section 4. When the cell completes the apoptosis death cycle time, it is removed from the simulation.

3.7.2 Necrosis

Sustained hypoxia, *i.e.* when the oxygen concentration is not enough to keep the cells alive, such as that encountered in ischemic tissue [52–54] and in larger tumors [55, 56], can lead to ATP depletion and consequently cell death. This unplanned cell death is referred to as *necrosis*.

When a cell becomes necrotic, its surface ion pumps cease to function, resulting in osmosis of water into the cell, cell swelling, and subsequent bursting [57]. This differs from apoptosis, where the volume loss is orderly and the intracellular contents are contained in apoptotic bodies [57]. In necrotic cells, the remaining solid cell fraction is generally not phagocytosed by surrounding cells, as they themselves are typically also necrotic. In some cancers (e.g., breast cancer [58], liver cancer [59], ovarian cancer [60], and lymphoma [61, 62]) and other pathologic conditions (e.g., tuberculosis [63] and abscesses [64, 65]), necrotic tissue can undergo calcification: the solid cell components are replaced by calcium phosphate and/or calcium oxalate molecules that bond together to form calcite crystals that grow into hard microcalcifications [66]. But it should be noted that there are several regulated pathways that also trigger necrosis cell death (Necroptosis, Ferroptosis, Pyroptosis, etc) [7].

To model necrosis we assume that any cell (in any cell cycle model) at any time interval $[t, t+\Delta t]$,

can enter the necrotic state, with rate r_{necrosis}. The necrosis death rate parameter is set dynamically according to the microenvironmental conditions (oxygen concentration) as shown in equation

$$r_{\text{necrosis}} = \begin{cases} r_{\text{necrosis}} & \text{if } O_2 < O_{2,\text{nec\_max}} \\ r_{\text{necrosis}} \left(\frac{O_{2,\text{nec\_limit}} - O_2}{O_{2,\text{nec\_limit}} - O_{2,\text{nec\_max}}} \right) & \text{if } O_{2,\text{nec\_limit}} \leq O_2 \leq O_{2,\text{nec\_limit}} \\ 0 & \text{if } O_{2,\text{nec\_limit}} < O_2 \end{cases}$$

where O_2 is the current oxygen concentration at the cell's voxel, $O_{2,\text{nec\_limit}}$ is the oxygen limit value at which necrosis starts and $O_{2,\text{nec\_max}}$ is the oxygen value at which necrosis reaches its maximum rate.

When a cell becomes necrotic it initially swell by oncosis (cell death-related swelling), ruptures (lysis), and slowly degrade. So when entering the necrotic death model we set $f_{CN} = V^*_{NS} = 0$ to model cytoplasmic and nuclear degradation, $f_F = 1$ to model oncosis and $f_F = 0$ to model lysis. The rate parameters r_F, r_N, and r_C are set to match expected time scales throughout necrosis. Please check section 4 for numerical values.

3.8 Cancer cell

The correct interpretation of growth and inhibitory signals is key to maintaining healthy tissues. If the cell receives both growth-promoting and -inhibiting signals, its behavior is determined by the balance of the signals and the resulting gene expression pattern. Two types of genes are particularly relevant to regulating cell proliferation. Oncogenes respond to or create growth signals and promote cell cycle progression. Tumor suppressor genes (TSGs) respond to inhibitory signals, retard or halt the cell cycle, ensure proper DNA repair, and may trigger apoptosis under certain circumstances. Cancer initiation, or carcinogenesis, starts with the malfunction of one or more of these types of genes [67]. Genetic mutations can cause overactivity in oncogenes and impair the function of tumor suppressor genes. Sometimes, a single uncorrected point mutation is sufficient to affect the function of an oncogene [68] or functionally neutralize a tumor suppressor gene [69]. In other cases, cell division errors (e.g., during M phase) can create a mutant fusion oncogene, where the

protein coding portion of an oncogene is mistakenly fused with the triggering portion of another, frequently expressed gene. As a result, signals are "misrouted" to the oncogene, thus boosting its activity. Because normal cells possess two copies of each tumor suppressor gene, both copies must be damaged for a total loss of function of the gene. While the probability of independent mutations in both copies of the TSG is ordinarily small, loss of heterozygosity (two damaged copies of the TSG are passed to a daughter cell) can significantly accelerate the process [70]. Furthermore, the loss of just one TSG copy can significantly impair its activity and increase the probability of completing a multi-step carcinogenesis pathway [71].

Next we will discuss the main differences between cancer cells and healthy cells. Our discussion primarily focuses upon carcinomas (cancers arising from epithelial cells).

3.8.1 Cycle

As we have mentioned before, cancer occurs when defective genes cause cells to malfunction and interact with the body in an aberrant, hyperproliferative manner (either by increased cell proliferation or reduced cell apoptosis). Thus, in contrast to healthy cells, cancer cells grow and divide in an uncontrolled manner, invading normal tissues and organs and eventually spreading throughout the body. To model this behavior, we use the same cell cycles as we did with healthy cells but cancer cells do not enter in the quiescent state after a given number of divisions. Therefore cancer cells proliferate indefinitely.

Another difference between healthy cells and cancer cells is the presence of a mutant oncoprotein, o, in the last. This mutant oncoprotein is assumed to increase immunogenicity proportionally to o, similarly to mutant tumor-associated epitopes being presented on MHCs (major histocompatibility complexes) [72, 73]. This is meant to model intratumoral heterogeneity. So, we assign each cell a random expression of this oncoprotein such that $0 \leq o \leq 2$, with a normal distribution with mean 1 and standard deviation of 0.25 approximately. Then we adjust the model to set the rate of the stochastic cell cycle entry to scale proportionally not only to oxygen but to the oncoprotein. Then, eq. 32 becomes:

$$r_{-+} = o\left[\frac{1}{t_{K-}}\max\left\{\left(\frac{O_2 - O_{2,prol}}{O_2^* - O_{2,prol}}\right), 0\right\}\right]. \tag{75}$$

Therefore an increased oncoprotein increases the rate of cell proliferation.

3.8.2 Secretion

In addition to the oxygen consumption that healthy cells also present, in our model, cancer cells secrete an immunostimulatory factor. This substrate models a chemokine such as basic fibroblast growth factor (bFGF) [74], which diffuses according to the differential equations we have previously introduced in sections 2 and 3.6.

3.8.3 Death

In our model cancer cells can also undergo two types of cell deaths: *Necrosis* and *Apoptosis*. The first one will be induced by the lack of nutrients, particularly oxygen, in the tumor microenvironment. The second one will be induced, for example, by lymphocytes when immunotherapies are applied [75]. Please refer to sections 3.7.2 and 3.7.1 respectively to learn more about those death models.

3.8.4 Angiogenesis

As we have mentioned before, in its first stage, the tumor has no vascular system of its own, and so it must rely upon the host vasculature in the nearby stroma for crucial oxygen, nutrients, and growth factors. This substrates diffuse from the surrounding vascularized tissue, enter the tumor, and are uptaken by tumor cells. The motion of substrates from external sources (the host vasculature) to internal sinks (the metabolically active tumor cells) causes substrate gradients to form within the tumor.

Of particular importance is oxygen, which generally diffuses on the order of $100 - 200\mu$m into tissue before dropping to levels insufficient for cellular metabolism [55, 56, 76]. Interior tumor cells experience hypoxia and respond to their harsher microenvironment in a variety of ways. Deeper

within the tumor, oxygen drops to critically low levels that cause the tumor cells to necrose. These dynamics are manifested as an outer tumor viable rim of proliferating cells, an interior band of hypoxic cells, and a central necrotic core. Once the cells reach this state, the next stage in cancer development, that can be viewed as a response to hypoxia, is angiogenesis. Angiogenesis is the formation of new blood vessels, in which the tumor induces endothelial cells to form a new vasculature that directly supplies the tumor with the nutrients, enabling further expansion. Some of the same mechanisms responsible for angiogenesis play a role in metastasis, the spread of tumor cells to distant locations.

We approximate this phenomena by using the Dirichlet nodes. They allow us to overwrite the substrates values so that there is a direct supply of oxygen and other nutrients within the tumor. With this fresh supply of nutrients, the tumor can now begin a new stage of rapid growth into the surrounding tissue.

Future improvements of our work will include excluded volume on the Dirichlet nodes, so the cancer cells would not step on the blood vessels.

4 Parameters for the main examples

4.1 General

Parameter	Value	Units	Reference
Microenvironment Parameters			
$\Omega_x \times \Omega_y \times \Omega_z$	$1000 \times 1000 \times 1000$	μm	-
$\Delta x \times \Delta y \times \Delta z$	$20 \times 20 \times 20$	μm	-
D_{O_2}	10^5	$\mu m^2/min$	[77]
λ_{O_2}	0.1	min^{-1}	[37]
D_{GF}	10^6	$\mu m^2/min$	-
λ_{GF}	0.5	min^{-1}	-
BC	No flux at the boundaries	-	-
$\rho_{vessels,O_2}$	38	mmHg	[78]
Cell Mechanics Parameters			
n_x, n_y, n_z	$30 \times 30 \times 30$	μm	-
R_A	$1.25R_{cell}$	μm	[40]
C_{ccr}	10.0ν	μm/min	[79]
C_{cmr}	C_{ccr}	μm/min	-
p	0	dimensionless	-

Table 4: Numerical values of the input parameters used in all the examples.

Time Parameters			
Parameter	Value	Units	Reference
t_{Tot}	43200	min	-
t_{save}	1440	min	-
Δt_{cycle}	6	min	[36]
Δt_{mech}	0.1	min	[36]
Δt_{diff}	0.01	min	[36]
t_v	$20\Delta t_{mech}$	min	-

Table 5: Times used in all the examples.

4.2 Liver regeneration

Parameter	Value	Units	Reference
Cell Parameters			
V	8000	μm^3	[80]
V_N	268	μm^3	[81]
O_2^*	38	mmHg	-
$O_{2,prol}$	10	mmHg	-
GF_{prol}	0.25	dimensionless	-
S_{O_2}	0	min^{-1}	-
U_{O_2}	10	min^{-1}	-
Cell Cycle Parameters			
t_{G1}	21.6	hs	[82]
t_S	7.0	hs	[82]
t_{G2}	3.4	hs	[82]
t_M	1.6	hs	[82]
n_{div}	1	dimensionless	[16]
div_{prob}	0.7	dimensionless	[16]

Table 6: Numerical values used for liver regeneration

4.3 Tumor growth

Parameter	Value	Units	Reference
Cell Parameters			
V	8000	μm^3	[80]
V_N	268	μm^3	[81]
O_2^*	38	mmHg	-
$O_{2,prol}$	10	mmHg	-
S_{O_2}	0	min^{-1}	-
U_{O_2}	10	min^{-1}	-
Cell Cycle Parameters			
t_{G1}	20.4	hs	[82]
t_S	13.6	hs	[82]
t_{G2}	3.0	hs	[82]
t_M	1.6	hs	[82]
n_{div}	∞	dimensionless	-

Table 7: Numerical values used for Hepatocellular carcinoma recurrence

5 References

1. Hoffmann K, Nagel AJ, Tanabe K, Fuchs J, Dehlke K, Ghamarnejad O, et al. Markers of liver regenerationthe role of growth factors and cytokines: a systematic review. BMC surgery. 2020;20(1):1–15. doi:10.1186/s12893-019-0664-8.

2. Ghaffarizadeh A, Friedman SH, Macklin P. BioFVM: an efficient, parallelized diffusive transport solver for 3-D biological simulations. Bioinformatics. 2015;32(8):1256–1258. doi:10.1093/bioinformatics/btv730.

3. Eymard R, Gallouët T, Herbin R. Finite Volume Methods. In: Lions JL, Ciarlet P, editors. Solution of Equation in ℝn (Part 3), Techniques of Scientific Computing (Part 3). vol. 7 of Handbook of Numerical Analysis. Elsevier; 2000. p. 713–1020. Available from: https://hal.archives-ouvertes.fr/hal-02100732.

4. Marchuk GI. Splitting and alternating direction methods. vol. 1 of Handbook of Numerical Analysis. Elsevier; 1990. p. 197–462. Available from: https://www.sciencedirect.com/science/article/pii/S1570865905800353.

5. Yanenko NN. In: Holt M, editor. Simple Schemes in Fractional Steps for the Integration of Parabolic Equations. Berlin, Heidelberg: Springer Berlin Heidelberg; 1971. p. 17–41. Available from: 10.1007/978-3-642-65108-3_2.

6. Thomas LH. Elliptic problems in linear difference equations over a network. Watson Sci Comput Lab Rept, Columbia University, New York. 1949;1:71.

7. Galluzzi L, Vitale I, Abrams J, Alnemri E, Baehrecke E, Blagosklonny M, et al. Molecular definitions of cell death subroutines: recommendations of the Nomenclature Committee on Cell Death 2012. Cell Death & Differentiation. 2012;19(1):107–120. doi:10.1038/s41418-017-0012-4.

8. Elledge SJ. Cell cycle checkpoints: preventing an identity crisis. Science. 1996;274(5293):1664–1672. doi:10.1126/science.274.5293.1664.

9. Zetterberg A, Larsson O, Wiman KG. What is the restriction point? Current Opinion in Cell Biology. 1995;7(6):835–842. doi:10.1016/0955-0674(95)80067-0.

10. Blagosklonny MV, Pardee AB. The restriction point of the cell cycle. Cell cycle. 2002;1(2):102–109. doi:10.4161/cc.1.2.108.

11. Clyde RG, Bown JL, Hupp TR, Zhelev N, Crawford JW. The role of modelling in identifying drug targets for diseases of the cell cycle. Journal of The Royal Society Interface. 2006;3(10):617–627. doi:10.1098/rsif.2006.0146.

12. Kerr JF, Winterford CM, Harmon BV. Apoptosis. Its significance in cancer and cancer therapy. Cancer. 1994;73(8):2013–2026. doi:10.1002/1097-0142(19940415)73:8¡2013::AID-CNCR2820730802¿3.0.CO;2-J.

13. Krysko DV, Berghe TV, DHerde K, Vandenabeele P. Apoptosis and necrosis: detection, discrimination and phagocytosis. Methods. 2008;44(3):205–221. doi:10.1016/j.ymeth.2007.12.001.

14. Palmes D, Spiegel HU. Animal models of liver regeneration. Biomaterials. 2004;25(9):1601–1611. doi:10.1016/S0142-9612(03)00508-8.

15. Alison M, Islam S, Lim S. Stem cells in liver regeneration, fibrosis and cancer: the good, the bad and the ugly. The Journal of Pathology. 2009;217(2):282–298. doi:10.1002/path.2453.

16. Miyaoka Y, Miyajima A. To divide or not to divide: revisiting liver regeneration. Cell division. 2013;8(1):1–12. doi:10.1186/1747-1028-8-8.

17. Miyaoka Y, Ebato K, Kato H, Arakawa S, Shimizu S, Miyajima A. Hypertrophy and Unconventional Cell Division of Hepatocytes Underlie Liver Regeneration. Current Biology. 2012;22(13):1166–1175. doi:10.1016/j.cub.2012.05.016.

18. Cienfuegos JA, Rotellar F, Baixauli J, Martínez-Regueira F, Pardo F, Hernández-Lizoáin JL. Regeneración hepática; el secreto mejor guardado: Una forma de respuesta al daño

tisular. Revista Española de Enfermedades Digestivas. 2014;106(3):171–194. doi:1130-0108/2014/106/3/171-194.

19. Bruno S, Darzynkiewicz Z. Cell cycle dependent expression and stability of the nuclear protein detected by Ki-67 antibody in HL-60 cells. Cell Proliferation. 1992;25(1):31–40. doi:10.1111/j.1365-2184.1992.tb01435.x.

20. Urruticoechea A, Smith IE, Dowsett M. Proliferation marker Ki-67 in early breast cancer. Journal of clinical oncology. 2005;23(28):7212–7220. doi:10.1200/JCO.2005.07.501.

21. Aoyagi M, Yamamoto M, Wakimoto H, Azuma H, Hirakawa K, Yamamoto K. Immunohistochemical detection of Ki-67 in replicative smooth muscle cells of rabbit carotid arteries after balloon denudation. Stroke. 1995;26(12):2328–2332. doi:10.1161/01.STR.26.12.2328.

22. Larsson S, Ryden T, Holst U, Oredsson S, Johansson M. Estimating the variation in S phase duration from flow cytometric histograms. Mathematical Biosciences. 2008;213(1):40–49. doi:10.1016/j.mbs.2008.01.009.

23. Lin W, Arthur G. Phospholipids are synthesized in the G2/M phase of the cell cycle. The International Journal of Biochemistry & Cell Biology. 2007;39(3):597–605. doi:10.1016/j.biocel.2006.10.011.

24. Albe KR, Butler MH, Wright BE. Cellular concentrations of enzymes and their substrates. Journal of theoretical biology. 1990;143(2):163–195. doi:10.1016/S0022-5193(05)80266-8.

25. Banks MA, Porter DW, Pailes WH, Schwegler-Berry D, Martin WG, Castranova V. Taurine content of isolated rat alveolar type I cells. Comparative biochemistry and physiology B, Comparative biochemistry. 1991;100(4):795–799. doi:10.1016/0305-0491(91)90292-l.

26. Howard J. Mechanics of motor proteins and the cytoskeleton. Sinauer associates Sunderland, MA; 2001.

27. Milo R, Phillips R. In: What is the density of cells? Garland Science; 2015.Available from: http://book.bionumbers.org/what-is-the-density-of-cells/.

28. Ahmad Khalili A, Ahmad MR. A review of cell adhesion studies for biomedical and biological applications. International journal of molecular sciences. 2015;16(8):18149–18184. doi:10.3390/ijms160818149.

29. Fletcher DA, Mullins RD. Cell mechanics and the cytoskeleton. Nature. 2010;463(7280):485–492. doi:10.1038/nature08908.

30. Pegoraro AF, Janmey P, Weitz DA. Mechanical properties of the cytoskeleton and cells. Cold Spring Harbor perspectives in biology. 2017;9(11):a022038. doi:10.1101/cshperspect.a022038.

31. Drasdo D. Buckling Instabilities of One-Layered Growing Tissues. Phys Rev Lett. 2000;84:4244–4247. doi:10.1103/PhysRevLett.84.4244.

32. Drasdo D, Loeffler M. Individual-based models to growth and folding in one-layered tissues: intestinal crypts and early development. Nonlinear Analysis: Theory, Methods & Applications. 2001;47(1):245–256. doi:10.1016/S0362-546X(01)00173-0.

33. Chu YS, Dufour S, Thiery JP, Perez E, Pincet F. Johnson-Kendall-Roberts theory applied to living cells. Physical review letters. 2005;94(2):028102. doi:10.1103/PhysRevLett.94.028102.

34. Pathmanathan P, Cooper J, Fletcher A, Mirams G, Murray P, Osborne J, et al. A computational study of discrete mechanical tissue models. Physical biology. 2009;6(3):036001. doi:10.1088/1478-3975/6/3/036001.

35. Byrne H, Drasdo D. Individual-based and continuum models of growing cell populations: a comparison. Journal of mathematical biology. 2009;58(4):657–687. doi:10.1007/s00285-008-0212-0.

36. Ghaffarizadeh A, Heiland R, Friedman SH, Mumenthaler SM, Macklin P. PhysiCell: An open source physics-based cell simulator for 3-D multicellular systems. PLOS Computational Biology. 2018;14(2):1–31. doi:10.1371/journal.pcbi.1005991.

37. Macklin P, Edgerton ME, Thompson AM, Cristini V. Patient-calibrated agent-based modelling of ductal carcinoma in situ (DCIS): From microscopic measurements to macro-

scopic predictions of clinical progression. Journal of Theoretical Biology. 2012;301:122–140. doi:10.1016/j.jtbi.2012.02.002.

38. DAntonio G, Macklin P, Preziosi L. An agent-based model for elasto-plastic mechanical interactions between cells, basement membrane and extracellular matrix. Mathematical biosciences and engineering: MBE. 2013;10(1):75. doi:10.3934/mbe.2013.10.75.

39. Jenner AL, Smalley M, Goldman D, Goins WF, Cobbs CS, Puchalski RB, et al. Agent-based computational modeling of glioblastoma predicts that stromal density is central to oncolytic virus efficacy. iScience. 2022;25(6). doi:10.1016/j.isci.2022.104395.

40. Mathias S, Coulier A, Bouchnita A, Hellander A. Impact of Force Function Formulations on the Numerical Simulation of Centre-Based Models. Bulletin of Mathematical Biology. 2020;82:1–43. doi:10.1007/s11538-020-00810-2.

41. Chiu CH, Leu JD, Lin TT, Su PH, Li WC, Lee YJ, et al. Systematic Quantification of Cell Confluence in Human Normal Oral Fibroblasts. Applied Sciences. 2020;10(24):9146. doi:10.3390/app10249146.

42. Lv D, Hu Z, Lu L, Lu H, Xu X. Three-dimensional cell culture: A powerful tool in tumor research and drug discovery. Oncology letters. 2017;14(6):6999–7010. doi:10.3892/ol.2017.7134.

43. Happel J, Brenner H. Low Reynolds number hydrodynamics: with special applications to particulate media. vol. 1. Springer Science & Business Media; 1991.

44. Purcell EM. Life at low Reynolds number. American Journal of Physics. 2000;45:3–11. doi:10.1103/PhysRevLett.84.4244.

45. Lauga E, Powers TR. The hydrodynamics of swimming microorganisms. Reports on progress in physics. 2009;72(9):096601. doi:10.1088/0034-4885/72/9/096601.

46. Wolgemuth CW. Collective swimming and the dynamics of bacterial turbulence. Biophysical journal. 2008;95(4):1564–1574. doi:10.1529/biophysj.107.118257.

47. Griffiths D, Higham DJ. Numerical Methods for Ordinary Differential Equations: Initial Value Problems. Springer Undergraduate Mathematics Series. Springer-Verlag; 2010.

48. Chapra SC. Applied Numerical Methods with MATLAB for Engineers and Scientists. 4th ed. McGraw-Hill Education; 2017.

49. Pöschel T, Schwager T. Computational Granular Dynamics: Models and Algorithms. 1st ed. Springer-Verlag; 2005.

50. Green DR. Means to an end: apoptosis and other cell death mechanisms. Cold Spring Harbor Laboratory Press; 2011.

51. Raychaudhuri S. A minimal model of signaling network elucidates cell-to-cell stochastic variability in apoptosis. PloS one. 2010;5(8):e11930. doi:10.1371/journal.pone.0011930.

52. Kloner R, Jennings R. Consequences of brief ischemia: stunning, preconditioning, and their clinical implications: part 1. Circulation. 2001;104 24:2981–9. doi:10.1161/hc4801.100038.

53. Kloner RA, Jennings RB. Consequences of brief ischemia: stunning, preconditioning, and their clinical implications: part 2. Circulation. 2001;104(25):3158–3167. doi:10.2174/138161206777947614.

54. Sairanen T, Szepesi R, Karjalainen-Lindsberg ML, Saksi J, Paetau A, Lindsberg PJ. Neuronal caspase-3 and PARP-1 correlate differentially with apoptosis and necrosis in ischemic human stroke. Acta neuropathologica. 2009;118(4):541–552. doi:10.1007/s00401-009-0559-3.

55. Carmeliet P, Jain RK. Angiogenesis in cancer and other diseases. nature. 2000;407(6801):249–257. doi:10.1038/35025220.

56. Fischer I, Gagner JP, Law M, Newcomb EW, Zagzag D. Angiogenesis in Gliomas: Biology and Molecular Pathophysiology. Brain Pathology. 2005;15(4):297–310. doi:10.1111/j.1750-3639.2005.tb00115.x.

57. Barros LF, Hermosilla T, Castro J. Necrotic volume increase and the early physiology of necrosis. Comparative Biochemistry and Physiology Part A: Molecular & Integrative Physiology. 2001;130(3):401–409. doi:10.1016/S1095-6433(01)00438-X.

58. Stomper PC, Margolin FR. Ductal carcinoma in situ: the mammographer's perspective. AJR American journal of roentgenology. 1994;162(3):585–591. doi:10.2214/ajr.162.3.8109501.

59. Hayat M. Methods of cancer diagnosis, therapy, and prognosis: liver cancer. vol. 5. Springer Science & Business Media; 2009.

60. Sohaib S, Reznek R. MR imaging in ovarian cancer. Cancer Imaging. 2007;7(Special issue A):S119. doi:10.1102/1470-7330.2007.9046.

61. Ishikawa T, Kobayashi Y, Omoto A, Adachi Y, Nakagawa S, Kaneko T, et al. Calcification in untreated non-Hodgkins lymphoma of the jejunum. Acta haematologica. 1999;102(4):185–189. doi:10.1159/000041012.

62. Choi JH, Jeong YY, Shin SS, Lim HS, Kang HK. Primary Calcified T-cell lymphoma of the urinary bladder: a case report. Korean journal of radiology. 2003;4(4):252–254. doi:10.3348/kjr.2003.4.4.252.

63. Basaraba RJ, Bielefeldt-Ohmann H, Eschelbach EK, Reisenhauer C, Tolnay AE, Taraba LC, et al. Increased expression of host iron-binding proteins precedes iron accumulation and calcification of primary lung lesions in experimental tuberculosis in the guinea pig. Tuberculosis. 2008;88(1):69–79. doi:10.1016/j.tube.2007.09.002.

64. Lamb MA CR, Kleine DVM LJ, McMillan MC. Diagnosis of calcification on abdominal radiographs. Veterinary radiology. 1991;32(5):211–220. doi:10.1111/j.1740-8261.1991.tb00109.x.

65. Wolf EK, Smidt AC, Laumann AE. Topical Sodium Thiosulfate Therapy for Leg Ulcers With Dystrophic Calcification. Archives of Dermatology. 2008;144(12):1560–1562. doi:10.1001/archderm.144.12.1560.

66. Majno G, Joris I. Cells, tissues, and disease: principles of general pathology. Oxford University Press; 2004.

67. Hanahan D, Weinberg RA. The hallmarks of cancer. cell. 2000;100(1):57–70. doi:10.1016/S0092-8674(00)81683-9.

68. Malumbres M, Barbacid M. RAS oncogenes: the first 30 years. Nature Reviews Cancer. 2003;3(6):459–465. doi:10.1038/nrc1097.

69. Horowitz JM, Yandell DW, Park SH, Canning S, Whyte P, Buchkovich K, et al. Point mutational inactivation of the retinoblastoma antioncogene. Science. 1989;243(4893):937–940. doi:10.1126/science.2521957.

70. Nowak MA, Komarova NL, Sengupta A, Jallepalli PV, Shih IM, Vogelstein B, et al. The role of chromosomal instability in tumor initiation. Proceedings of the National Academy of Sciences. 2002;99(25):16226–16231. doi:10.1073/pnas.202617399.

71. Quon KC, Berns A. Haplo-insufficiency? Let me count the ways. Genes & development. 2001;15(22):2917–2921. doi:10.1101/gad.949001.

72. Khanna R. Tumour surveillance: missing peptides and MHC molecules. Immunology and cell biology. 1998;76(1):20–26. doi:10.1046/j.1440-1711.1998.00717.x.

73. Comber JD, Philip R. MHC class I antigen presentation and implications for developing a new generation of therapeutic vaccines. Therapeutic advances in vaccines. 2014;2(3):77–89. doi:10.1177/2051013614525375.

74. Ichim CV. Revisiting immunosurveillance and immunostimulation: Implications for cancer immunotherapy. Journal of translational medicine. 2005;3(1):1–13. doi:10.1186/1479-5876-3-8.

75. Yu J, Green MD, Li S, Sun Y, Journey SN, Choi JE, et al. Liver metastasis restrains immunotherapy efficacy via macrophage-mediated T cell elimination. Nature Medicine. 2021;27(1):152–164. doi:10.1038/s41591-020-1131-x.

76. Cristini V, Lowengrub J, Nie Q. Nonlinear simulation of tumor growth. Journal of mathematical biology. 2003;46(3):191–224. doi:10.1007/s00285-002-0174-6.

77. Grote J, Süsskind R, Vaupel P. Oxygen diffusivity in tumor tissue (DS-carcinosarcoma) under temperature conditions within the range of 20–40 C. Pflügers Archiv. 1977;372(1):37–42. doi:10.1007/BF00582204.

78. McKeown S. Defining normoxia, physoxia and hypoxia in tumoursimplications for treatment response. The British journal of radiology. 2014;87(1035):20130676. doi:10.1259/bjr.20130676.

79. Matthews BD, Overby DR, Mannix R, Ingber DE. Cellular adaptation to mechanical stress: role of integrins, Rho, cytoskeletal tension and mechanosensitive ion channels. Journal of Cell Science. 2006;119(3):508–518. doi:10.1242/jcs.02760.

80. Morales-Navarrete H, Segovia-Miranda F, Klukowski P, Meyer K, Nonaka H, Marsico G, et al. A versatile pipeline for the multi-scale digital reconstruction and quantitative analysis of 3D tissue architecture. Elife. 2015;4:e11214. doi:10.7554/eLife.11214.

81. Peters R. Nucleo-cytoplasmic flux and intracellular mobility in single hepatocytes measured by fluorescence microphotolysis. The EMBO journal. 1984;3(8):1831–1836. doi:10.1002/j.1460-2075.1984.tb02055.x.

82. Rotstein J, Macdonald PDM, Rabes HM, Farber E. Cell Cycle Kinetics of Rat Hepatocytes in Early Putative Preneoplastic Lesions in Hepatocarcinogenesis. Cancer Research. 1984;44(7):2913–2917.
